# Gastrointestinal adverse events associated with GLP-1 RA in non-diabetic patients with overweight or obesity: a systematic review and network meta-analysis

**DOI:** 10.1038/s41366-025-01859-6

**Published:** 2025-08-13

**Authors:** Abdulrahman Ismaiel, Giuseppe Guido Maria Scarlata, Irina Boitos, Daniel-Corneliu Leucuta, Stefan-Lucian Popa, Nahlah Al Srouji, Ludovico Abenavoli, Dan L. Dumitrascu

**Affiliations:** 1 2nd Department of Internal Medicine, “Iuliu Hatieganu” University of Medicine and Pharmacy, Cluj-Napoca, Romania; 2 Department of Health Sciences, University “Magna Graecia”, Catanzaro, Italy; 3 Faculty of Medicine, “Iuliu Hatieganu” University of Medicine and Pharmacy, Cluj-Napoca, Romania; 4 Department of Medical Informatics and Biostatistics, “Iuliu Hatieganu” University of Medicine and Pharmacy, Cluj-Napoca, Romania

**Keywords:** Obesity, Obesity, Weight management

## Abstract

**Introduction:**

Overweight and obesity are major global health issues, increasing disease risk and straining healthcare systems. Glucagon-like peptide-1 receptor agonists (GLP-1 RAs) are effective for weight loss but cause gastrointestinal side effects, affecting adherence. Research often focuses on diabetics, leaving a gap in understanding their effects on non-diabetic individuals with overweight or obesity. This systematic review and dose-response network meta-analysis addresses this gap, analyzing gastrointestinal adverse events from GLP-1 RAs in non-diabetic subjects with overweight or obesity.

**Methods:**

We evaluated available evidence by searching PubMed and EMBASE databases, according to specific inclusion and exclusion eligibility criteria to evaluate gastrointestinal adverse events associated with GLP-1 RAs in non-diabetic individuals with overweight or obesity. Quality assessment of included studies was conducted using Cochrane Collaboration’s tool.

**Results:**

Thirty-nine articles were included in the review showing a total number of 33,354 individuals. Nausea, vomiting, diarrhea, and constipation were the most common gastrointestinal adverse effects. All evaluated GLP-1 RAs led to a significant increase in nausea risk, with orforglipron showing the highest risk, followed by exenatide, tirzepatide, semaglutide, and liraglutide. Additionally, liraglutide, orforglipron, semaglutide, and tirzepatide were associated with increased vomiting risk, while cagrilinitide and exenatide showed no significant increase. Exenatide, cagrilinitide, orforglipron were not associated with diarrhea risk. Finally, semaglutide and liraglutide were associated to increased constipation risk, while cagrilinitide and exenatide showed no significant increase.

**Conclusions:**

GLP-1 RAs showed several adverse gastrointestinal effects in non-diabetic patients with overweight or obesity. Understanding the different risk profiles of GLP-1 RAs helps clinicians make informed treatment decisions by balancing therapeutic benefits with potential side effects.

## Introduction

Overweight and obesity have emerged as pressing public health concerns worldwide, with escalating prevalence rates over the past few decades. These conditions not only pose significant challenges to individual health but also impose substantial burdens on healthcare systems globally [1]. According to recent epidemiological studies, the prevalence of overweight and obesity has reached alarming levels, affecting individuals across all age groups and socioeconomic strata. The multifaceted nature of overweight and obesity intertwines with various comorbidities, including cardiovascular diseases, type 2 diabetes mellitus (T2DM), musculoskeletal disorders, and certain cancers, thereby underscoring the imperative for effective management strategies [2].

Current guidelines advocate for a multifaceted approach to weight management, integrating lifestyle modifications, dietary interventions, and pharmacotherapy [3]. However, achieving sustainable weight loss remains a formidable challenge for many individuals. While behavioral modifications and dietary restrictions constitute cornerstone interventions, pharmacological agents play a pivotal role, particularly in cases where lifestyle interventions alone prove insufficient [4].

Among the pharmacological agents, glucagon-like peptide-1 receptor agonists (GLP-1 RAs) have gained prominence for their efficacy in facilitating weight loss in individuals with overweight or obesity. GLP-1 RAs exert their therapeutic effects through multiple mechanisms, primarily by stimulating the GLP-1 receptor, which in turn enhances insulin secretion, suppresses glucagon release, delays gastric emptying, and promotes satiety. Consequently, these agents not only aid in glycemic control but also exhibit pronounced effects on body weight [5].

A wide range of GLP-1 receptor agonists are currently approved or in clinical development for weight loss or glycemic control. These include short-acting agents (e.g., exenatide BID), long-acting formulations (e.g., liraglutide, semaglutide, dulaglutide), dual agonists such as tirzepatide (GIP/GLP-1 RA), and novel oral agents like orforglipron and danuglipron. Additionally, long-acting amylin analogues like cagrilintide are being investigated in combination with GLP-1 RAs for enhanced weight loss efficacy. A complete overview of these agents, their mechanisms, and development phases is provided in Table 1.

**Table 1:** Overview of GLP-1 receptor agonists and related agents in clinical use or development for weight loss or glycemic control.

Agent	Mechanism	Class	Route	Dosing frequency	FDA status	Clinical phase	Notes
Exenatide (Byetta)	GLP-1 RA	Short-acting GLP-1 RA	Subcutaneous	Twice daily	Approved (2005)	Marketed	First GLP-1 RA on the market
Exenatide ER (Bydureon)	GLP-1 RA	Long-acting GLP-1 RA	Subcutaneous	Once weekly	Approved (2012)	Marketed	Extended-release formulation
Liraglutide (Saxenda/Victoza)	GLP-1 RA	Long-acting GLP-1 RA	Subcutaneous	Once daily	Approved (2010/2014)	Marketed	Saxenda approved for obesity
Semaglutide (Ozempic/Wegovy/Rybelsus)	GLP-1 RA	Long-acting GLP-1 RA	Subcutaneous (Wegovy/Ozempic), Oral (Rybelsus)	Weekly (SC), Daily (PO)	Approved (2017–2021)	Marketed	Oral semaglutide is the first oral GLP-1 RA
Dulaglutide (Trulicity)	GLP-1 RA	Long-acting GLP-1 RA	Subcutaneous	Once weekly	Approved (2014)	Marketed	Less GI side effects compared to others
Albiglutide (Tanzeum)	GLP-1 RA	Long-acting GLP-1 RA	Subcutaneous	Once weekly	Withdrawn	Withdrawn	Voluntarily withdrawn for commercial reasons
Tirzepatide (Mounjaro/Zepbound)	Dual GIP + GLP-1 RA	Dual incretin receptor agonist	Subcutaneous	Once weekly	Approved (2022–2023)	Marketed	Dual agonist with greater weight loss efficacy
Orforglipron	GLP-1 RA (non-peptide, oral)	Oral small molecule GLP-1 RA	Oral	Once daily	Investigational	Phase 3	First oral non-peptide GLP-1 RA
Danuglipron	GLP-1 RA (non-peptide, oral)	Oral small molecule GLP-1 RA	Oral	Twice daily	Investigational	Phase 2	GI tolerability issues noted in early trials
Cagrilintide	Long-acting amylin analogue	Amylin analogue	Subcutaneous	Once weekly	Investigational	Phase 3 (in combination)	Combined with semaglutide in ongoing obesity trials
Efinopegdutide	GLP-1/FGF21 dual agonist	Dual agonist	Subcutaneous	Once weekly	Investigational	Phase 2	Novel mechanism under investigation

Despite their therapeutic benefits, GLP-1 RAs are associated with a spectrum of adverse events, with gastrointestinal disturbances being among the most commonly reported. Gastrointestinal adverse events represent a notable concern in individuals receiving GLP-1 RAs, potentially impacting treatment adherence and patient satisfaction. These adverse events encompass a range of symptoms, including nausea, vomiting, diarrhea, and abdominal discomfort, which may vary in severity and duration.

While the exact mechanisms underlying these gastrointestinal disturbances remain incompletely elucidated, they are believed to stem from the actions of GLP-1 RAs on gastrointestinal motility and secretion [6]. Despite the burgeoning literature on the safety and efficacy of GLP-1 RAs, existing systematic reviews and meta-analyses predominantly focus on populations with T2DM, where gastrointestinal symptoms may be confounded by the underlying disease pathology [7].

Consequently, there is a paucity of comprehensive evidence elucidating the incidence and impact of gastrointestinal adverse events specifically in non-diabetic patients with overweight and obesity receiving GLP-1 RAs for weight loss purposes. Thus, the primary objective of this systematic review and dose-response network meta-analysis is to comprehensively evaluate the diverse gastrointestinal adverse events associated with GLP-1 RAs in this distinct patient population. Through rigorous synthesis and analysis of available data, we aimed to provide valuable insights into the safety profile of GLP-1 RAs in non-diabetic individuals with overweight or obesity, thereby informing clinical decision-making and optimizing patient care in this vulnerable population.

## Materials and methods

This systematic review and meta-analysis was written according to the 2020 Preferred Reporting Items for Systematic Reviews and Meta-Analyses (PRISMA) guidelines [8].

### Data sources and search strategy

Our aim was to evaluate available evidence by searching the PubMed and EMBASE databases, employing the research strategy outlined in Supplementary Material 1. Additionally, we manually screened the references of included articles to minimize the risk of overlooking relevant studies. The search encompassed articles published from inception to December 20, 2023, with no limitations on timeframe, geographical location, or language. Screening involved initial assessment of titles and abstracts for relevance, followed by a thorough examination of full texts based on predetermined inclusion and exclusion criteria. Study eligibility was independently assessed by two authors, with data extraction carried out by two other authors, and any discrepancies resolved through consensus.

### Eligibility criteria

Inclusion criteria for original articles encompassed: (1) interventional studies (clinical trials, randomized controlled trials) evaluating gastrointestinal adverse events associated with GLP-1 RAs in non-diabetic patients with overweight or obesity; (2) human studies; and (3) articles published in English, French, German, Romanian, or Italian. (4) Although our primary focus was on GLP-1 RAs, we included trials evaluating agents with overlapping mechanisms, such as tirzepatide (dual GIP/GLP-1 RA) and cagrilintide (long-acting amylin analogue), due to their emerging relevance in obesity treatment.

Exclusion criteria comprised: (1) individuals with T2DM; (2) editorial, letter, case report, conference abstract, systematic review, guideline, commentary, or abstract-only publications; and (3) experimental studies.

### Risk of bias assessment in individual studies

The Cochrane Collaboration’s tool was employed to evaluate the risk of bias in randomized controlled trials [9]. This assessment focused on various factors including randomized sequence generation, treatment allocation concealment, blinding, completeness of outcome data, as well as selective outcome reporting and other potential sources of bias.

We applied these assessment criteria consistently to gauge bias risk and internal validity within each study. Two authors (G.G.M.S and I.B.) independently assessed the risk of bias in individual studies. Any discrepancies were resolved through discussion to reach a consensus.

### Summary measures and synthesis of results

The principal summary outcome was the relative risk (RR) of several GI adverse events in people with overweight or obesity receiving treatment with GLP-1 RA, including nausea, vomiting, diarrhea, constipation, abdominal distension, upper abdominal pain, abdominal pain, abdominal discomfort, gastroesophageal reflux disease, (GERD), eructation, flatulence, decreased appetite, cholelithiasis, gallstone-related, cholecystitis, acute cholecystitis, acute pancreatitis, viral gastroenteritis, hard feces, and infrequent bowel movements. For the summary outcomes, we computed the estimates of the random effects using restricted maximum likelihood to estimate the heterogeneity variance, since we assumed clinical variability between the studies. We conducted data analyses within R environment for statistical computing and graphics (R Foundation for Statistical Computing, Vienna, Austria), version 4.1.2, using the netmeta R package [10]. Between-study heterogeneity was evaluated using the χ2 based Q-test and I^2^. Firstly, we used a frequentist approach to network meta-analysis. Network graphs were used to describe the comparisons between drugs. The direct and indirect proportion for each comparison was plotted. Data were reported from each study within forest plots as the estimated RR with a 95% confidence interval (CI). The ranking of the GI adverse events was computed using P-scores and presented graphically and as the order of the interventions in text. Next, we carried out a dose-response network meta-analysis using the MBNMAdose R package version 0.4.2. Network dose-response meta-analysis charts for adverse effects were plotted, illustrating the relationships and comparisons between different doses and treatments. Network dose-response meta-analysis estimates by dose, and nonparametric monotonic increasing model plots were employed to assess the trends in adverse effects with increasing doses. Network meta-analysis forest plots and cumulative ranking plots are shown too. A statistically significant *p* value was considered when <0.05. The analyses were conducted if two or more studies evaluated similar groups and reported the same outcome.

## Results

### General results

**Fig. 1:**
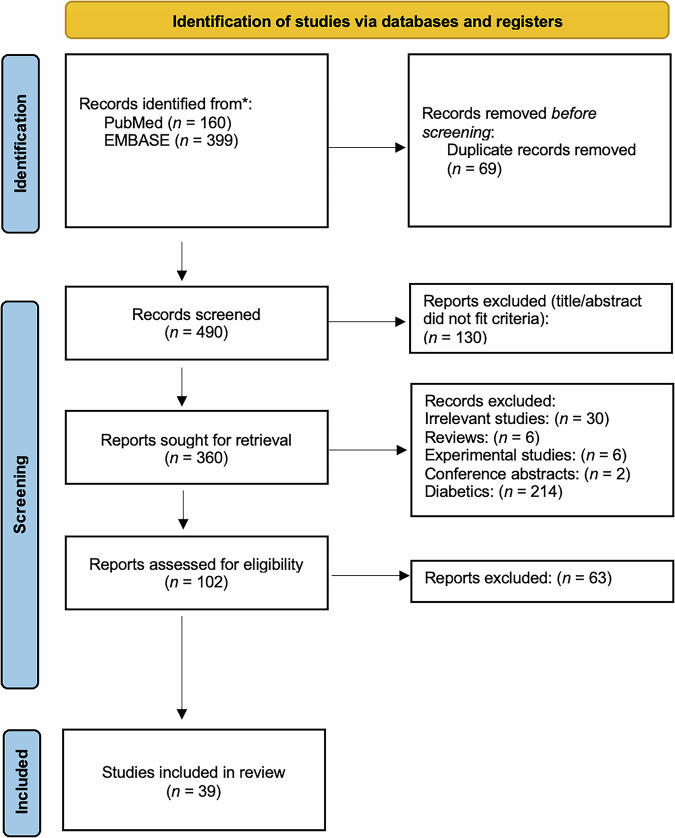
PRISMA flow diagram. The PRISMA flow diagram outlining the stages of identification, screening, and inclusion.

Figure 1 represents the PRISMA flow diagram, illustrating the search strategy employed. Initially, a total of 559 articles were retrieved (PubMed *n* = 160, EMBASE *n* = 399). Subsequently, 69 duplicate articles were identified and removed. Subsequently, 490 articles underwent preliminary screening based on title and abstract for inclusion/exclusion criteria assessment. During this phase, 130 articles were excluded. A total of 360 articles were considered for full-text retrieval, but 258 articles were further excluded upon reading. A comprehensive assessment of the full texts of the remaining 102 articles was performed for further eligibility evaluation. Of these, 63 articles were excluded based on various reasons, as demonstrated in Supplementary Material 2. Consequently, 39 articles were included in the systematic review and dose-response network meta-analysis [11–49].

### Study characteristics

The main characteristics of included studies are summarized in Supplementary Table 1. This systematic review and meta-analysis included a total number of 33,354 individuals. Thirteen studies were performed in the United States [11, 15–17, 20, 22, 35, 36, 40, 43–46], 9 in Denmark [12, 13, 18, 19, 21, 24, 28, 31, 34], 4 both in China [23, 33, 48, 49] and Slovenia [25–27, 39], 3 in Canada [14, 29, 47], 2 in Italy [37, 38], 1 in United Kingdom [30], Sweden [32], Australia [41], and Netherlands [42], respectively.

### Network meta-analysis of gastrointestinal adverse effects

Table 2 and Supplementary Table 2 summarize the obtained network meta-analysis results for the assessed GI adverse events associated with GLP-1 RA in people with overweight and obesity.

**Table 2:** Summary of relative risks (RR) for common gastrointestinal adverse events associated with GLP-1 and related agents in non-diabetic individuals with overweight or obesity.

Drug	Nausea RR (95% CI)	Vomiting RR (95% CI)	Diarrhea RR (95% CI)	Constipation RR (95% CI)	Abdominal pain RR (95% CI)
*Semaglutide*	2.95 (2.61–3.32)	4.21 (3.58–4.95)	1.77 (1.47–2.14)	2.10 (1.67–2.63)	2.34 (1.41–3.89)
*Liraglutide*	3.09 (2.73–3.51)	3.87 (3.15–4.76)	1.82 (1.48–2.25)	2.24 (1.74–2.87)	2.08 (1.06–4.07)
*Tirzepatide*	2.90 (2.00–4.19)	13.23 (4.85–36.09)	3.35 (1.92–5.85)	3.36 (1.70–6.63)	4.36 (1.29–14.78)
*Cagrilinitide*	2.30 (1.69–3.13)	1.37 (0.81–2.32)	1.20 (0.68–2.10)	1.28 (0.70–2.35)	NA
*Orforglipron*	4.77 (2.02–11.31)	4.43 (1.45–13.56)	2.30 (0.89–5.93)	4.05 (1.19–13.78)	1.46 (0.26–8.18)
*Exenatide*	2.66 (1.40–5.09)	4.52 (0.25–82.77)	0.18 (0.02–1.34)	5.00 (0.24–105.07)	0.33 (0.01–7.95)

### Nausea

**Fig. 2:**
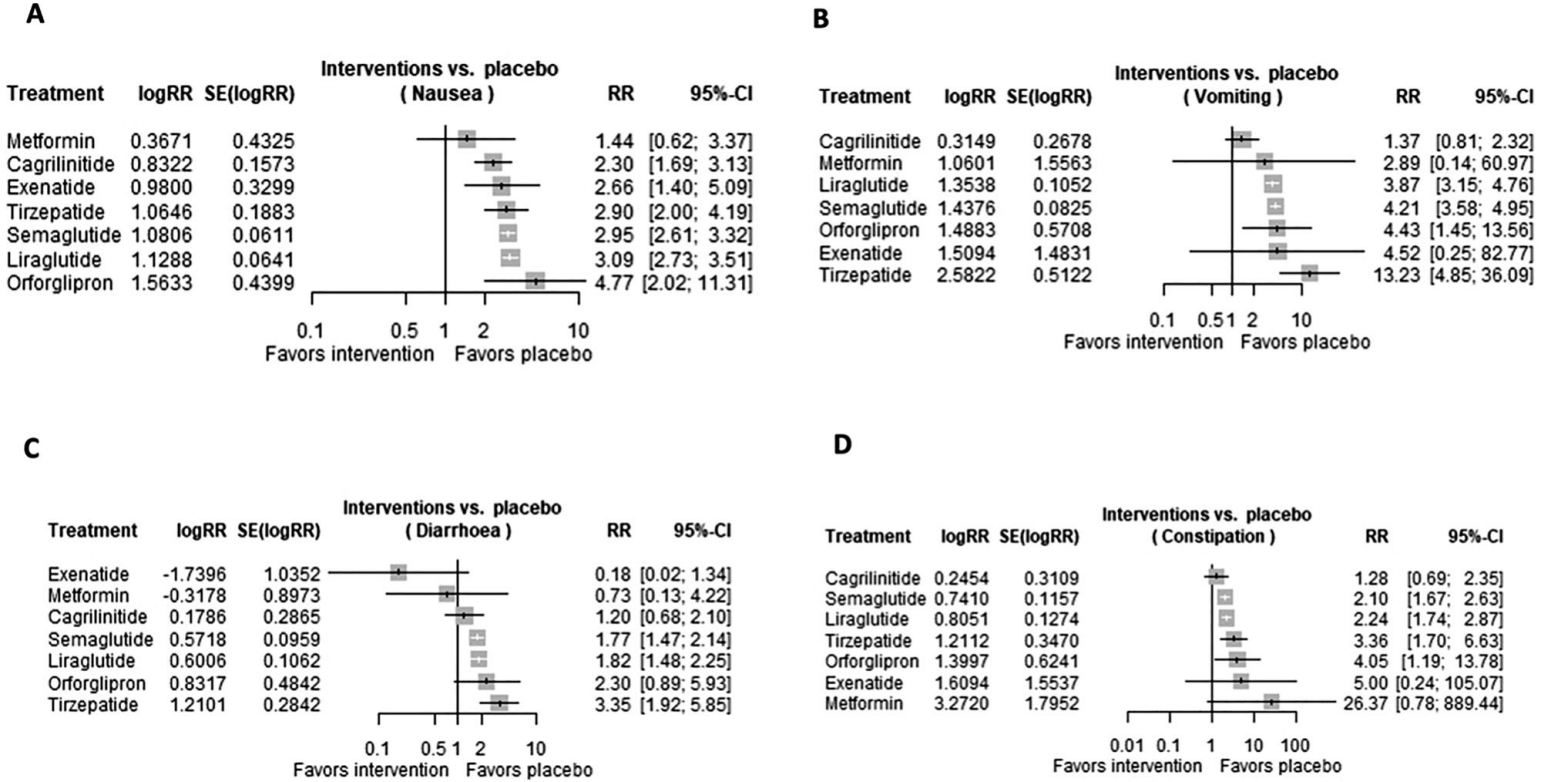
Risk of nausea, vomiting, diarrhea, and constipation in GLP-1 RAs. Forest plots regarding the risk of (**A**) nausea, **B** vomiting, **C** diarrhea, and **D** constipation in patients receiving GLP-1 RAs.

A total of 29 trials were evaluated regarding the risk of nausea in patients receiving GLP-1 RAs. All evaluated GLP-1 RAs were associated with a significant increased risk of nausea, in the following order: cagrilinitide (RR 2.2983 [95% CI 1.6884, 3.1285; *p*-value < 0.0001]), exenatide (RR 2.6645 [95% CI 1.3956, 5.0870; *p*-value 0.0030]), tirzepatide (RR 2.8997 [95% CI 2.0048, 4.1939; *p*-value < 0.0001]), semaglutide (RR 2.9464 [95% CI 2.6138, 3.3213; *p*-value < 0.0001]), liraglutide (RR 3.0919 [95% CI 2.7271, 3.5056; *p*-value < 0.0001]), orforglipron (RR 4.7748 [95% CI 2.0161, 11.3085; *p*-value 0.0004], as shown in Fig. 2A.

### Vomiting

A total of 23 trials were evaluated regarding the risk of vomiting in patients receiving GLP-1 RAs. Some of the evaluated GLP-1 RAs were associated with significant increased risk of vomiting, in the following order: liraglutide (RR 3.8722 [95% CI 3.1510, 4.7585; *p*-value < 0.0001]), orforglipron (RR 3.8722 [95% CI 1.4471, 13.5578; *p*-value 0.0091]), semaglutide (RR 4.2108 [95% CI 3.5822, 4.9497; *p*-value < 0.0001]), tirzepatide (RR 13.2265 [95% CI 4.8471, 36.0917; *p*-value < 0.0001]). On the contrary, the following GLP-1 RAs were not associated with a significant increased risk of vomiting: cagrilinitide (RR 1.3701 [95% CI 0.8106, 2.3158; *p*-value 0.2397]), exenatide (RR 4.5238 [95% CI 0.2472, 82.7745; *p*-value 0.3088]), as shown in Fig. 2B.

### Diarrhea

A total of 24 trials were evaluated regarding the risk of diarrhea in patients receiving GLP-1 RAs. Some of the evaluated GLP-1 RAs were associated with significant increased risk of diarrhea, in the following order: semaglutide (RR 1.7714 [95% CI 1.4679, 2.1377; *p*-value < 0.0001]), liraglutide (RR 1.8231 [95% CI 1.4804, 2.2452; *p*-value < 0.0001]), tirzepatide (RR 3.3537 [95% CI 1.9216, 5.8533; *p*-value < 0.0001]). On the contrary, the following GLP-1 RAs were not associated with a significant increased risk of diarrhea: exenatide (RR 0.1756 [95% CI 0.0231, 1.3355; *p*-value 0.0929]), cagrilinitide (RR 1.1956 [95% CI 0.6819, 2.0961; *p*-value 0.5329]), orforglipron (RR 2.2973 [95% CI 0.8893, 5.9347; *p*-value 0.0859]), as shown in Fig. 2C.

### Constipation

A total of 24 trials were evaluated regarding the risk of constipation in patients receiving GLP-1 RAs. Some of the evaluated GLP-1 RAs were associated with a significant increased risk of constipation, in the following order: semaglutide (RR 2.0979 [95% CI 1.6722, 2.6321; *p*-value < 0.0001]), liraglutide (RR 2.2368 [95% CI 1.7424, 2.8715; *p*-value < 0.0001]), tirzepatide (RR 3.3575 [95% CI 1.7007, 6.6282; *p*-value 0.0005]). On the contrary, the following GLP-1 RAs were not associated with significant increased risk of constipation: cagrilinitide (RR 1.2782 [95% CI 0.6950, 2.3508; *p*-value 0.4299]), exenatide (RR 5.0000 [95% CI 0.2379, 105.0709; *p*-value 0.3003]), orforglipron (RR 4.0541 [95% CI 1.1930, 13.7766; *p*-value 0.0249]), as shown in Fig. 2D.

### Abdominal distention

**Fig. 3:**
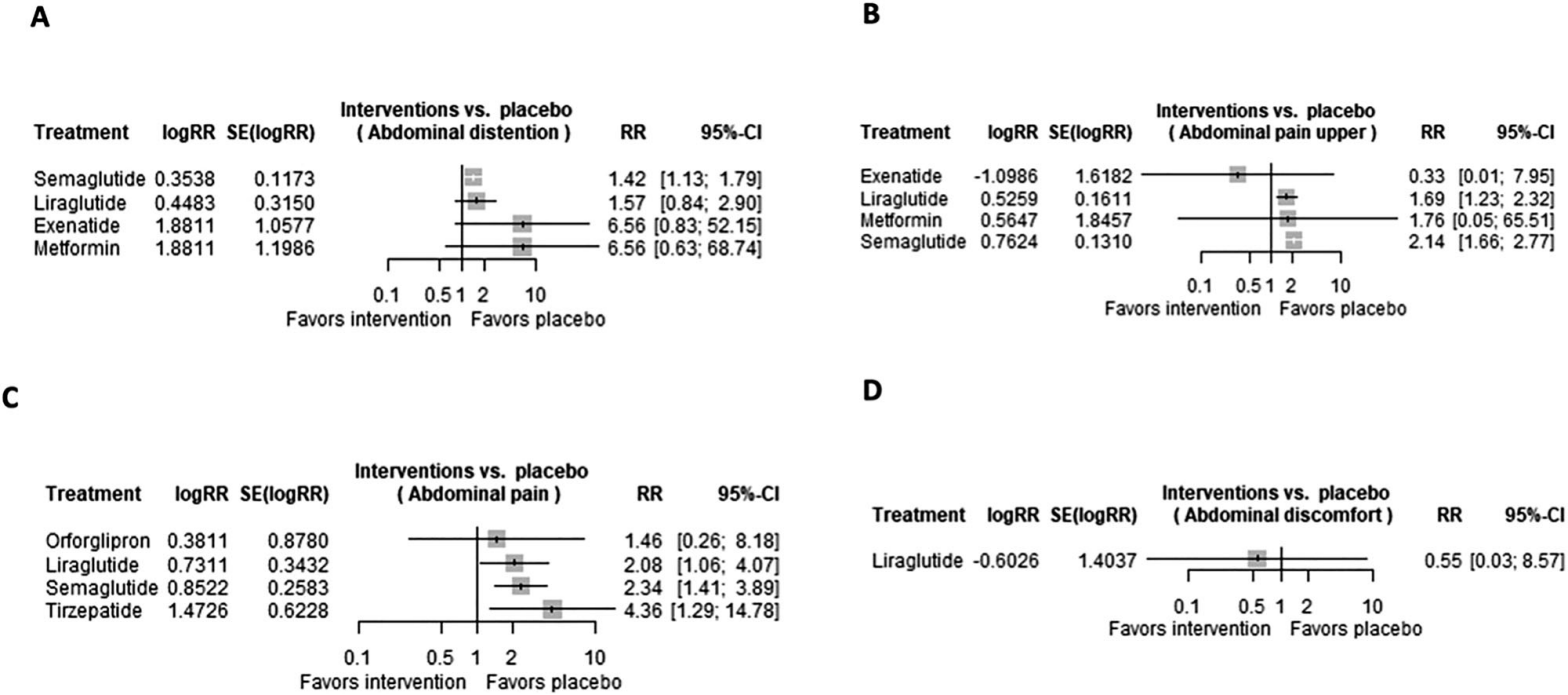
Risk of abdominal symptoms in GLP-1 RAs. Forest plots regarding the risk of (**A**) abdominal distention, **B** abdominal pain upper, **C** abdominal pain, and **D** abdominal discomfort in patients receiving GLP-1 RAs.

A total of 11 trials were evaluated regarding the risk of abdominal distention in patients receiving GLP-1 RAs. Only semaglutide (RR 1.4245 [95% CI 1.1320, 1.7925; *p*-value 0.0026] was associated with a significantly increased risk of abdominal distention, while liraglutide (RR 1.5656 [95% CI 0.8445, 2.9025; *p*-value 0.1547]), and exenatide (RR 6.5609 [95% CI 0.8253, 52.1544; *p*-value 0.0753]), were not associated with a significantly increased risk of abdominal distention, as shown in Fig. 3A.

### Upper abdominal pain

A total of 13 trials were evaluated regarding the risk of upper abdominal pain in patients receiving GLP-1 RAs. Liraglutide (RR 1.6919 [95% CI 1.2339, 2.3199; *p*-value 0.0011]), and semaglutide (RR 2.1435 [95% CI 1.6582, 2.7709; *p*-value < 0.0001]) were associated with significant increased risk of upper abdominal pain, while exenatide (RR 0.3333, [95% CI 0.0140, 7.9482; *p*-value 0.4972]) was not associated with significant increased risk of upper abdominal pain, as shown in Fig. 3B.

### Abdominal pain

A total of 12 trials were evaluated regarding the risk of abdominal pain in patients receiving GLP-1 RAs. Some of the evaluated GLP-1 RAs were associated with a significant increased risk of abdominal pain, in the following order: liraglutide (RR 2.077 [95% CI 1.0602, 4.0703; *p*-value 0.0331]), semaglutide (RR 2.3447 [95% CI 1.4134, 3.8898; *p*-value 0.0010]), tirzepatide (RR 4.3604 [95% CI 1.2866, 14.7780; *p*-value 0.0181]). On the contrary, orforglipron (RR 1.4640 [95% CI 0.2619, 8.1832; *p*-value 0.6642]) was not associated with significant increased risk of abdominal pain, as shown in Fig. 3C.

### Abdominal discomfort

Only one trial was evaluated regarding the risk of abdominal discomfort in patients receiving GLP-1 RAs. In this regard, liraglutide (RR 0.5474 [95% CI 0.0350, 8.5718; *p*-value 0.6677]) was not associated with a significant increased risk of abdominal discomfort, as shown in Fig. 3D.

### Gastroesophageal reflux disease

**Fig. 4:**
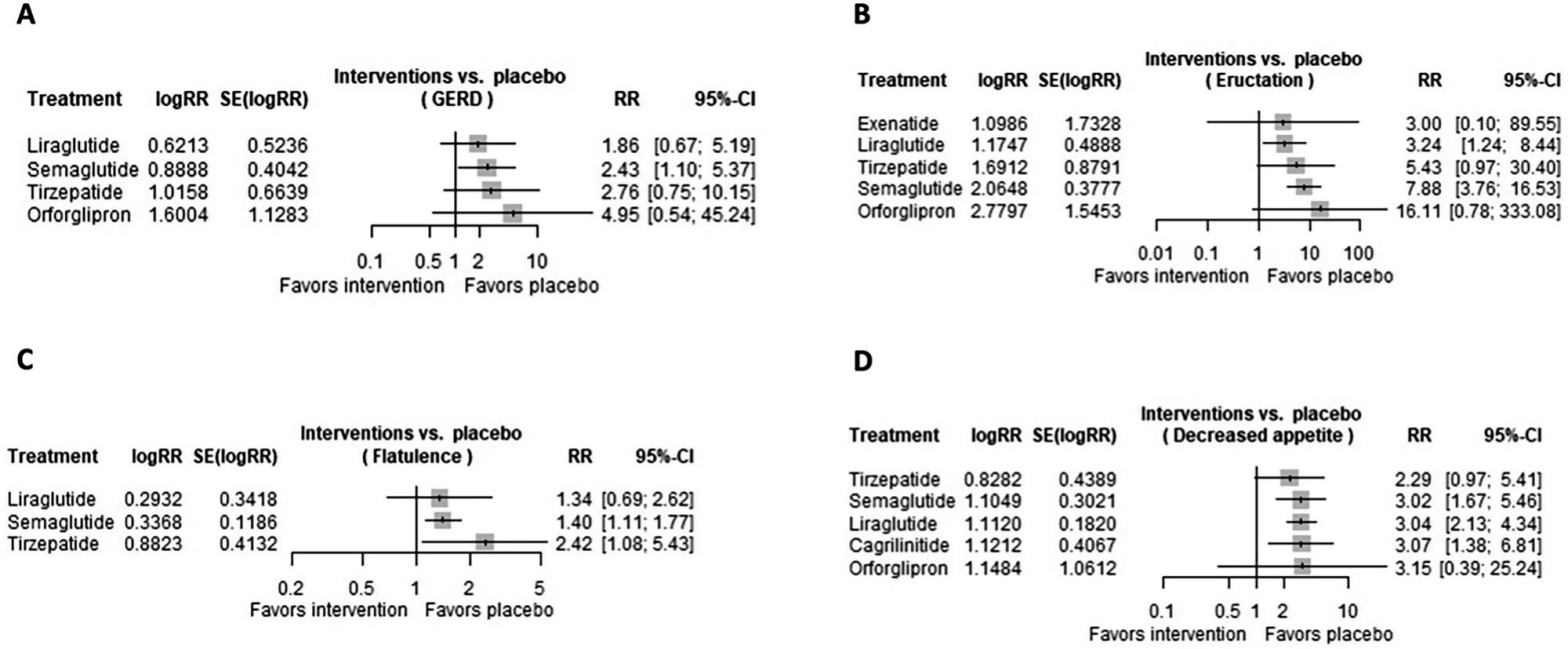
Risk of GERD, eructation, flatulence, and decreased appetite in GLP-1 RAs. Forest plots regarding the risk of (**A**) GERD, **B** eructation, **C** flatulence, and **D** decreased appetite in patients receiving GLP-1 RAs.

A total of 8 trials were evaluated regarding the risk of GERD in patients receiving GLP-1 RAs. Only semaglutide (RR 2.4321 [95% CI 1.1013, 5.3710; *p*-value 0.0279] was associated with significant increased risk of GERD. On the contrary, the following GLP-1 RAs were not associated with significant increased risk of GERD: liraglutide (RR 1.8614 [95% CI 0.6671, 5.1939; *p*-value 0.2354]), tirzepatide (RR 2.7616 [95% CI 0.7517, 10.1459; *p*-value 0.1260]), orforglipron (RR 4.9550 [95% CI 0.5427, 45.2367; *p*-value 0.1561]), as shown in Fig. 4A.

### Eructation

A total of 11 trials were evaluated regarding the risk of eructation in patients receiving GLP-1 RAs. Specifically, liraglutide (RR 3.2373 [95% CI 1.2419, 8.4388; *p*-value 0.0163]) and semaglutide (RR 7.8838 [95% CI 3.7606, 16.5278; *p*-value < 0.0001]) were associated with significant increased risk of eructation. On the contrary, exenatide (RR 3.0000 [95% CI 0.1005, 89.5452; *p*-value 0.5261]), tirzepatide (RR 5.4262 [95% CI 0.9687, 30.3963; *p*-value 0.0544]), and orforglipron (RR 16.1146 [95% CI 0.7796, 333.0789; *p*-value 0.0720]) were not associated with significant increased risk of eructation, as shown in Fig. 4B.

### Flatulence

A total of 8 trials were evaluated regarding the risk of flatulence in patients receiving GLP-1 RAs. Specifically, semaglutide (RR 1.4005 [95% CI 1.1100, 1.7671; *p*-value 0.0045]) and tirzepatide (RR 2.4164 [95% CI 1.0751, 5.4309; *p*-value 0.0327]) were associated with significant increased risk of flatulence. Only liraglutide (RR 1.3407 [95% CI 0.6862, 2.6198; *p*-value 0.3909]) was not associated with significant increased risk of flatulence, as shown in Fig. 4C.

### Decreased appetite

A total of 6 trials were evaluated regarding the risk of decreased appetite in patients receiving GLP-1 RAs. Specifically, semaglutide (RR 3.0190 [95% CI 1.6699, 5.4580; *p*-value 0.0003]), cagrilinitide (RR 3.0684 [95% CI 1.3826, 6.8099; *p*-value 0.0058]), and liraglutide (RR 3.0404 [95% CI 2.1283, 4.3434; *p*-value < 0.0001]) were associated with significant increased risk of decreased appetite. On the other hand, tirzepatide (RR 2.2892 [95% CI 0.9685, 5.4110; *p*-value 0.0592]), and orforglipron (RR 3.1532 [95% CI 0.9685, 5.4110; *p*-value 0.0592]) were not associated with a significant increased risk of decreased appetite, as shown in Fig. 4D.

### Cholelithiasis

**Fig. 5:**
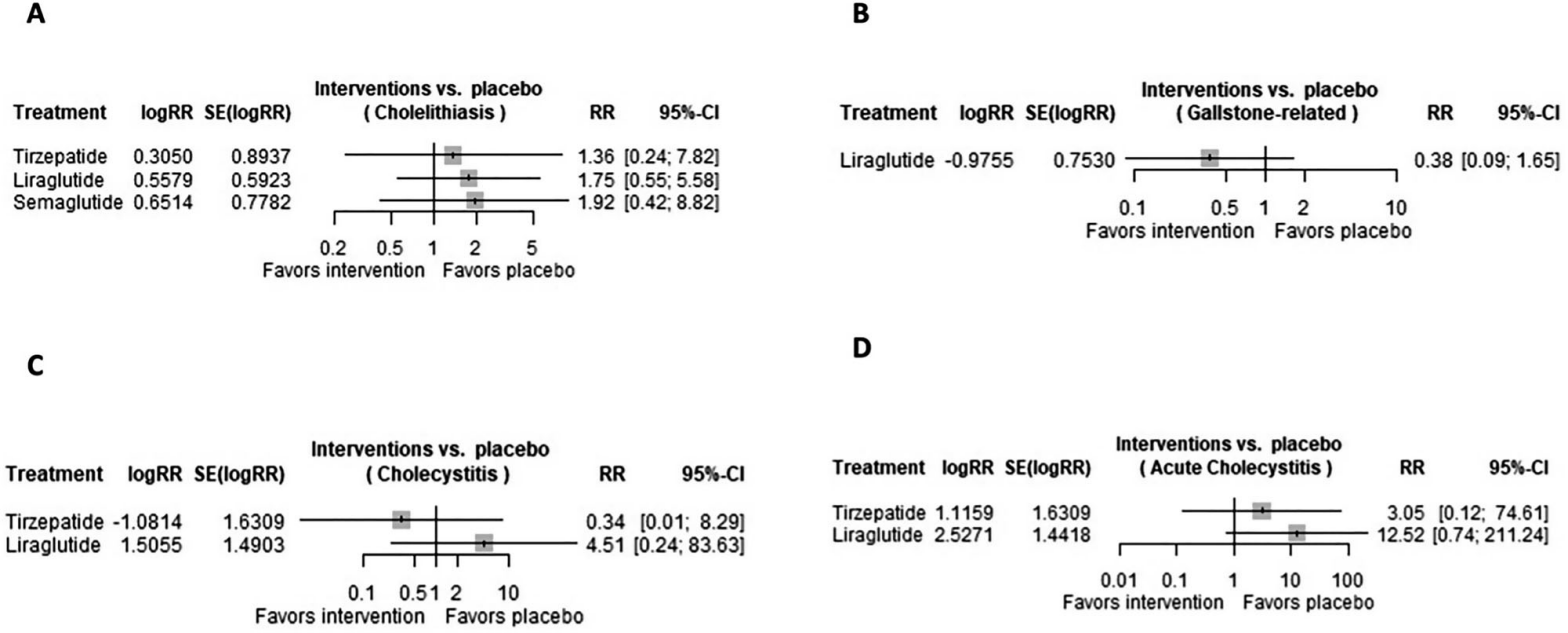
Risk of gallbladder disease in GLP-1 RAs. Forest plots regarding the risk of (**A**) cholelithiasis, **B** gallstone-related, **C** cholecystitis, and **D** acute cholecystitis in patients receiving GLP-1 RAs.

A total of 4 trials were evaluated regarding the risk of cholelithiasis in patients receiving GLP-1 RAs. All evaluated GLP-1 RAs were not associated with significant increased risk of cholelithiasis, in the following order: tirzepatide (RR 1.3566 [95% CI 0.2354, 7.8184; *p*-value 0.7329]), liraglutide (RR 1.7470 [95% CI 0.5471, 5.5783; *p*-value 0.3462]), and semaglutide (RR 1.9181 [95% CI 0.2354, 7.8184; *p*-value 0.7329]), as shown in Fig. 5A.

### Gallstone-related

A total of 2 trials were evaluated regarding the risk of gallstone-related in patients receiving GLP-1 RAs. Only liraglutide (RR 0.3770 [95% CI 0.0862, 1.6494; *p*-value 0.1952]) has been evaluated, showing no significant increased risk of gallstone-related, as reported in Fig. 5B.

### Cholecystitis

A total of 2 trials were evaluated regarding the risk of cholecystitis in patients receiving GLP-1 RAs. Both tirzepatide (RR 0.3391 [95% CI 0.0139, 8.2904; *p*-value 0.5073]) and liraglutide (RR 4.5063 [95% CI 0.2428, 83.6327; *p*-value 0.3124]) were not associated with significant increased risk of cholecystitis, as shown in Fig. 5C.

### Acute cholecystitis

A total of 2 trials were evaluated regarding the risk of acute cholecystitis in patients receiving GLP-1 RAs. Both tirzepatide (RR 3.0522 [95% CI 0.1249, 74.6134; *p*-value 0.4938]) and liraglutide (RR 12.5176 [95% CI 0.7418, 211.2448; *p*-value 0.0796]) were not associated with significant increased risk of acute cholecystitis, as shown in Fig. 5D.

### Acute pancreatitis

**Fig. 6:**
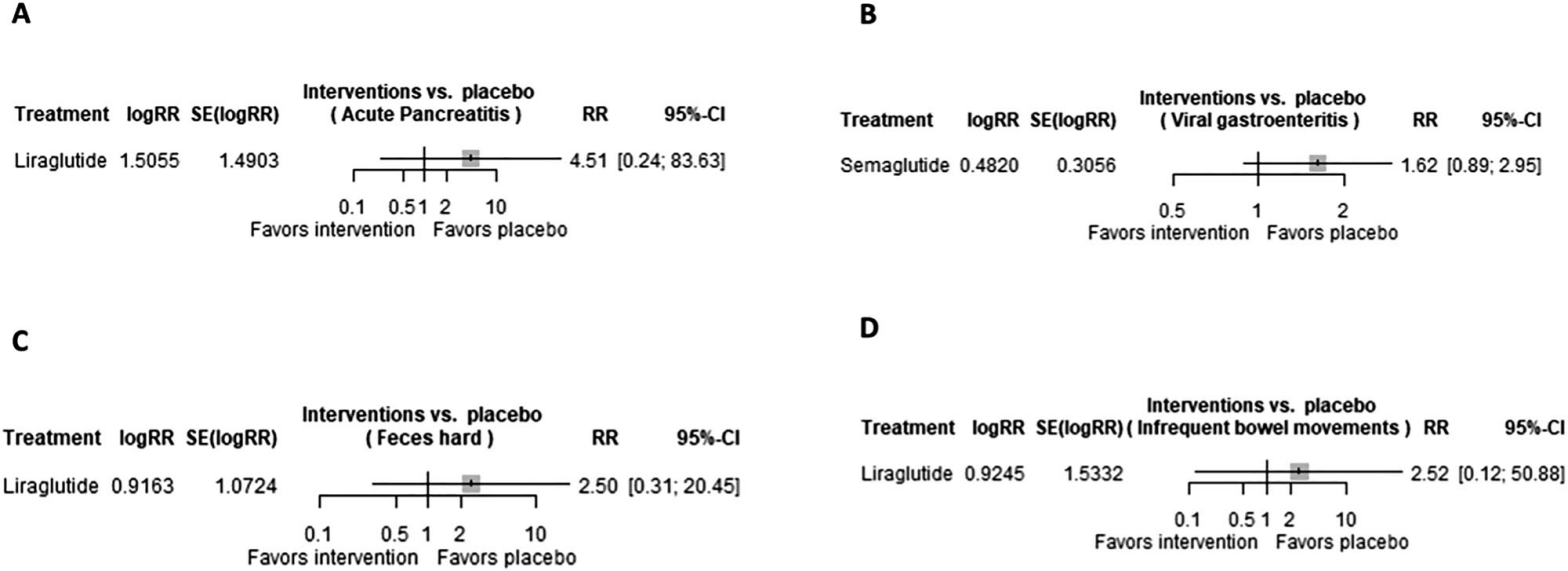
Risk of pancreatitis, gastroenteritis, and bowel movement disorders in GLP-1 RAs. Forest plot regarding the risk of (**A**) acute pancreatitis, **B** viral gastroenteritis, **C** feces hard, and **D** infrequent bowel movements in patients receiving GLP-1 RAs.

Only one trial was evaluated regarding the risk of acute pancreatitis in patients receiving GLP-1 RAs. In this regard, liraglutide (RR 4.5063 [95% CI 0.2428, 83.6327; *p*-value 0.3124]) was not associated with a significant increased risk of acute pancreatitis, as shown in Fig. 6A.

### Viral gastroenteritis

Only one trial was evaluated regarding the risk of viral gastroenteritis in patients receiving GLP-1 RAs. In this regard, semaglutide (RR 1.6194 [95% CI 0.8897, 2.9474; *p*-value 0.1147]) was not associated with a significant increased risk of viral gastroenteritis, as shown in Fig. 6B.

### Hard feces

Only one trial was evaluated regarding the risk of hard feces in patients receiving GLP-1 RAs. In this way, liraglutide (RR 2.5000 [95% CI 0.3056, 20.4528; *p*-value 0.3929]) was not associated with significant increased risk of feces hard, as shown in Fig. 6C.

### Infrequent bowel movements

Only one trial was evaluated regarding the risk of infrequent bowel movements in patients receiving GLP-1 RAs. In this way, liraglutide (RR 2.5207 [95% CI 0.1249, 50.8829; *p*-value 0.5465]) was not associated with a significant increased risk of infrequent bowel movements, as shown in Fig. 6D.

### Dose-response network meta-analysis

We proceeded to analyze the effects of dose on adverse events risk, through a dose-response network meta-analysis. All analyses performed have been included in Supplementary Figs. 1–10.

### Nausea

For all GLP-1 RAs, increasing dose was associated with increased risk of nausea. The risk of nausea was increasing for the lowest dosages at a faster rate, followed by a tendency to plateauing at highest dosages.

### Vomiting

A similar trend was observed for vomiting for most GLP-1 RAs, except for Exenatide, for which the risk increased linearly and at very high values. The relative risk values were higher compared to those observed for nausea. The most precise estimates were for liraglutide and semaglutide, where the confidence intervals were narrower.

### Diarrhea

For cagrilinitide and semagludide the risk of diarrhea was increasing for the lowest dosages at a faster rate, followed by a tendency to plateauing at highest dosages. For other GLP-1 RAs, the pattern seemed more like a linear increase with the dosage (liraglutide, orforglipron). The relative risk values were lower compared to those observed for nausea.

### Constipation

The risk of constipation was increasing for the lowest dosages at a faster rate, followed by a tendency to plateauing at highest dosages. The most precise estimates were for liraglutide and semaglutide, where the confidence intervals were narrower.

### Decreased appetite

For cagrilinitide and semagludide the risk of decreased appetite was increasing for the lowest dosages at a faster rate, followed by a tendency to plateauing at highest dosages. For other GLP-1 RAs, the pattern seemed more like a linear increase with the dosage (liraglutide, semaglutide FE). The most precise estimates were for semaglutide, where the confidence intervals were narrower.

### Quality assessment

All included articles were evaluated using the Cochrane Collaboration’s tool [11–49], as outlined in Supplementary Table 3. Several issues were reported regarding bias in the assessed articles. Overall, thirteen articles presented a low risk of bias for each domain [12–14, 17, 18, 20–22, 29, 34, 35, 43, 45]. Moreover, fourteen articles had an unclear risk of selection bias for random sequence generation and allocation concealment [11, 15, 19, 24, 26, 27, 30, 32, 39–42, 46, 47], thirteen articles presented unclear risk of bias for blinding [15, 19, 24, 28, 31, 32, 36–38, 41, 44, 46, 47], while three articles showed an unclear risk for attrition bias, reporting bias and other sources of bias [23, 27, 31]. Regarding the blinding within performance and detection bias, we found a high risk of bias in ten studies [16, 23, 25–27, 33, 39, 41, 48, 49].

## Discussion

The escalating prevalence of overweight and obesity globally has prompted the exploration of various management strategies, including pharmacotherapy, to address this pressing public health concern. GLP-1 RAs have garnered attention for their efficacy in facilitating weight loss in individuals with overweight or obesity [50]. However, their use is not without adverse effects, particularly gastrointestinal disturbances, which can impact treatment adherence and patient satisfaction [6]. Our systematic review and dose-response network meta-analysis aimed to comprehensively evaluate the gastrointestinal adverse events associated with the use of GLP-1 RAs in non-diabetic patients with overweight or obesity. Through rigorous synthesis and analysis of available data, we aimed to provide valuable insights into the safety profile of GLP-1 RAs in this distinct patient population.

Our findings reveal that all evaluated GLP-1 RAs were associated with a significant increased risk of nausea. Notably, orforglipron exhibited the highest risk, followed by exenatide, tirzepatide, semaglutide, and liraglutide. Nausea was the most frequently reported symptom, besides vomiting and diarrhea, findings consistent with prior research in diabetic populations [51–56].

The risk of vomiting varied, with liraglutide, orforglipron, semaglutide, and tirzepatide showing significant increases. However, cagrilinitide and exenatide were not associated with a significant increased risk of vomiting. A recent meta-analysis performed on patients with T2DM showed that all GLP-1 dose groups had a significantly higher incidence of vomiting compared to both placebo and conventional therapy, especially in individuals treated with exenatide [51]. Likewise, compared to placebo, several GLP-1 RAs including orforglipron, tirzepatide, semaglutide, liraglutide, and exenatide were associated with a significantly increased risk of vomiting [53].

Diarrhea was significantly more common with semaglutide, liraglutide, and tirzepatide, while cagrilinitide, exenatide, and orforglipron were not associated with increased risk. According to recent studies, tirzepatide, orforglipron, semaglutide, liraglutide, and exenatide were positively associated with diarrhea when compared with placebo, while liraglutide was associated with diarrhea when compared with exenatide and placebo [51, 53, 56]. Constipation was more frequent with semaglutide, liraglutide, and tirzepatide. However, cagrilinitide, exenatide, and orforglipron did not exhibit a significant increase in constipation risk. As recently reported, these symptoms were significantly higher in diabetic patients treated with high-dose of GLP-1 RAs compared to dipeptidyl peptidase 4 inhibitors [54].

Among less frequently assessed adverse events, semaglutide was uniquely associated with increased GERD risk. This gastrointestinal adverse effect was reported in 7% of diabetic patients treated with semaglutide but without performing an evaluation of the relative risk [57]. Eructation risk was significant for liraglutide and semaglutide, while exenatide, tirzepatide, and orforglipron did not show a significant increase. Additionally, flatulence risk was significantly higher after semaglutide and tirzepatide use. These findings are in line with recent reports highlighting semaglutide’s association with multiple GI disturbances [58].

Abdominal symptoms such as distention, pain, and discomfort varied across agents. However, certain receptor agonists did not exhibit a significant increase in these symptoms. At the same time, no significant increase in risk was observed for acute pancreatitis. On the contrary, a recent evaluation showed a significant risk of pancreatitis after semaglutide and exenatide use [59]. However, abdominal symptoms are often related to acute pancreatis, but extensive cardiovascular outcome trials have not demonstrated an elevated risk of pancreatitis associated with GLP-1RAs [60]. The risk of gallstone-related complications, cholelithiasis, cholecystitis, and acute cholecystitis did not significantly increase with GLP-1 RAs use. On the contrary, Monami et al. found that GLP-1RAs significantly increase the risk of gallstone disease in patients with T2DM [61].

Finally, viral gastroenteritis, feces hardness, or infrequent bowel movements showed no significant increased risk for adverse gastrointestinal events. These gastrointestinal adverse events are poorly documented in the literature due to their low frequency [6]. Despite the limited data available, these results contribute to our understanding of the broader gastrointestinal effects profile of GLP-1 RAs.

The dose-response network meta-analysis showed that increasing doses of GLP-1 RAs heightened the risk of nausea, vomiting, diarrhea, constipation, and decreased appetite. The most common pattern was that the risks of adverse effects rose quickly at lower doses before plateauing, or sometimes an increasing risk linear trend was observed.

The potential impact of diabetes status on the risk of gastrointestinal adverse events during GLP-1 RA therapy is an important clinical consideration. Some mechanistic hypotheses suggest that diabetic patients, due to altered gastric emptying, autonomic neuropathy, or baseline gastrointestinal dysmotility, may be more susceptible to the GI side effects associated with GLP-1 RAs. However, the evidence from our systematic review and network meta-analysis does not allow for a direct comparison, as most included studies enrolled exclusively non-diabetic populations, without providing stratified data. This limits our ability to determine whether diabetes itself constitutes an independent risk factor for adverse gastrointestinal outcomes with these agents. Future trials should report age- and disease-stratified data to better clarify whether patient-specific factors, such as diabetes, influence tolerability and safety profiles.

The results of this network meta-analysis support a more personalized approach when selecting GLP-1 RAs for weight management in non-diabetic individuals. The clinical relevance of gastrointestinal adverse events associated with GLP-1 RAs depends not only on their incidence but also on their severity, duration, and impact on patient quality of life. Even mild but persistent symptoms can lead to treatment discontinuation, particularly in non-diabetic individuals using these agents primarily for weight management, who may have lower thresholds for tolerability than diabetic patients. Reported discontinuation rates due to gastrointestinal side effects in diabetic populations range from 10–20%, and similar or higher rates may be expected in non-diabetic settings. These findings emphasize the need for a personalized approach in clinical decision-making, balancing efficacy goals with tolerability to optimize adherence and long-term outcomes. For patients with concerns about gastrointestinal discomfort, agents with lower associated risks such as dulaglutide, cagrilintide, or oral semaglutide may be preferable. Conversely, patients seeking maximal weight loss who can tolerate more adverse effects may benefit from agents like subcutaneous semaglutide or tirzepatide. Shared decision-making, gradual dose titration, patient education, and close monitoring are key strategies to mitigate adverse effects and improve satisfaction. Clinicians should also account for individual lifestyle factors, comorbidities, and treatment preferences. Future studies should aim to define tolerance thresholds and identify predictors of adherence, while future guidelines should incorporate tolerability data alongside efficacy in recommending GLP-1 RAs for this expanding patient population.

The main limitations of our study include a significant heterogeneity in terms of study design, patient populations, GLP-1 RA dosage regimens, and duration of follow-up. This variability could have impacted the consistency of the results and the overall interpretation of gastrointestinal adverse events. Furthermore, the quality of evidence across studies varied, with some trials having a high risk of bias due to issues such as inadequate blinding, incomplete outcome data, and selective reporting. These factors could influence the reliability of their findings. Some gastrointestinal adverse events like viral gastroenteritis, feces hardness, and infrequent bowel movements were reported in a limited number of trials, restricting the ability to perform robust statistical analyses and draw definitive conclusions for those specific outcomes. Although we focused on non-diabetic individuals with overweight and obesity, potential confounding factors such as concomitant medications, baseline gastrointestinal conditions, and varying lifestyle factors could have influenced the incidence and severity of reported adverse events. Due to lack of stratified data by time points across studies, we were unable to perform a subgroup analysis based on duration of treatment, which limited our ability to evaluate time-dependent risk trends of gastrointestinal adverse events. The clinical relevance of age-related differences in gastrointestinal adverse events is an important consideration. However, all included studies enrolled adult participants, precluding analyses in pediatric or adolescent populations. Additionally, the majority of studies did not report adverse events stratified by age within adults, thus preventing subgroup analyses comparing adult and elderly subjects. Future research should consider age-stratified data to better elucidate potential differential risks. At the same time, the majority of included studies were conducted in specific geographic regions (e.g., United States, Denmark, China), which may not represent the global population. Indeed, the generalizability of findings to other regions and ethnic groups remains uncertain. Finally, differences in the reporting and classification of gastrointestinal adverse events across studies could introduce inconsistencies in the data synthesis and interpretation.

The strengths of our investigation lie in the methodological rigor and the high number of studies examined. Indeed, this research underscores the benefits of performing an extensive literature review, which greatly reduces the likelihood of overlooking crucial studies and guarantees a robust data set for analysis. Employing an advanced analytical method, network meta-analysis and dose response network meta-analysis, the research offers broader comparative insights than traditional techniques that analyze treatments individually. Furthermore, the study carefully selects trials that focus exclusively on the GI adverse effects of GLP-1 RA in RCTs involving adults with overweight or obesity. This ensures the findings are highly relevant and directly applicable to this particular patient population. This targeted approach enhances the clarity and relevance of the results, providing a solid foundation for the conclusions. Additionally, to our knowledge, this is the first network meta-analysis concerning the association between GLP-1 RAs and gastrointestinal adverse events in the population with overweight or obesity, as these evaluations have been predominantly conducted in diabetic patients.

## Conclusions and future directions

This study provides a comprehensive evaluation of gastrointestinal adverse events associated with GLP-1 RAs in non-diabetic individuals with overweight or obesity. Nausea, vomiting, diarrhea, and constipation emerged as the most common adverse effects. All studied agents significantly increased the risk of nausea, with orforglipron showing the highest risk, followed by exenatide, tirzepatide, semaglutide, and liraglutide. Moreover, liraglutide, orforglipron, semaglutide, and tirzepatide also increased the risk of vomiting, while cagrilinitide and exenatide did not. Furthermore, exenatide, cagrilinitide, and orforglipron were not associated with an increased risk of diarrhea, and constipation was primarily linked to semaglutide and liraglutide. The dose-response meta-analysis showed that higher doses of GLP-1 RAs were associated with increased risks of nausea, vomiting, diarrhea, constipation, and decreased appetite. Most adverse effects rose sharply at lower doses and plateaued at higher doses.

These findings offer critical insights for clinicians prescribing GLP-1 RAs in non-diabetic patients, highlighting the need to weigh adverse effect profiles alongside therapeutic benefits. Personalized treatment strategies, guided by both efficacy and tolerability, are essential to optimize outcomes in this growing patient population. Further research should explore the underlying mechanisms of these adverse events, develop mitigation strategies, and assess differences across demographic groups. Trials with stratified data by age, disease status, and treatment duration will be instrumental in refining patient-centered prescribing practices.

## Supplementary information

Supplementary Material 1

Supplementary Material 2

Studies evaluating GI adverse events with GLP-1 agonists in subjects with overweight and obesity

Network meta-analysis summary for the assessed gastrointestinal adverse events associated with GLP-1 receptor agonists in subjects with overweight and obesity

Supplementary Table 3

Supplementary figures

## Data Availability

The analyzed data was extracted from the cited original articles as outlined in Supplementary Table 1.
