# Supplementary material for: Gastrointestinal adverse events associated with GLP-1 RA in non-diabetic patients with overweight or obesity: a systematic review and network meta-analysis
Source: Int J Obes (Lond). 2025 Aug 13;49(10):1946–57. doi: 10.1038/s41366-025-01859-6 (PMC12532569; doi:10.1038/s41366-025-01859-6)
Supplement: Supplementary file 1 — Supplementary Material 1 [file 41366_2025_1859_MOESM1_ESM.docx]

**Supplementary Material 1**

**20.12.2023**

**PubMed:** 167 articles

(("Glucagon-Like Peptide-1 Receptor Agonists"[Mesh]) OR ("Glucagon-Like Peptide-1 Receptor Agonists"[All Fields]) OR ("Exenatide"[Mesh]) OR ("Exenatide"[All Fields]) OR ("Liraglutide"[Mesh]) OR ("Liraglutide"[All Fields]) OR ("rGLP-1 protein" [Supplementary Concept]) OR ("taspoglutide" [Supplementary Concept]) OR ("lixisenatide" [Supplementary Concept]) OR ("semaglutide" [Supplementary Concept]) OR ("dulaglutide" [Supplementary Concept])) AND (("Randomized Controlled Trial" [Publication Type])) AND (("Weight Loss"[Mesh]) OR ("Weight Loss"[All Fields])) AND (("Obesity"[Mesh]) OR ("Obesity"[All Fields]))

**EMBASE:** 399 articles

('glucagon-like peptide-1 receptor agonists' OR 'exenatide'/exp OR 'exenatide' OR 'liraglutide'/exp OR 'liraglutide' OR 'rglp-1 protein' OR 'taspoglutide' OR 'lixisenatide' OR 'semaglutide' OR 'dulaglutide') AND 'randomized controlled trial' AND ('weight loss'/exp OR 'weight loss') AND ('obesity'/exp OR 'obesity') AND ('clinical article'/de OR 'clinical trial'/de OR 'clinical trial topic'/de OR 'controlled clinical trial topic'/de OR 'intervention study'/de OR 'multicenter study'/de OR 'multicenter study topic'/de OR 'randomized controlled trial'/de OR 'randomized controlled trial topic'/de) AND 'article'/it
