# Supplementary material for: Gastrointestinal adverse events associated with GLP-1 RA in non-diabetic patients with overweight or obesity: a systematic review and network meta-analysis
Source: Int J Obes (Lond). 2025 Aug 13;49(10):1946–57. doi: 10.1038/s41366-025-01859-6 (PMC12532569; doi:10.1038/s41366-025-01859-6)
Supplement: Supplementary file 2 — Supplementary Material 2 [file 41366_2025_1859_MOESM2_ESM.docx]

**Supplementary Material 2**

List of excluded articles following full-text assessment:

Irrelevant studies (*n*=5) [29-30,42,51,59], reviews (*n*=10) [1,6-9,17,28,38,46,61], conference abstracts (*n*=1) [3], duplicates (*n*=10) [12,15,20,27,37,47,52,60,62,63], patients with T2DM (*n*=11) [4,10,19,21-22,36,40,44,49,55,57], no mention of adverse effects (*n*=14) [2,5,11,13,24-26,32,39,43,45,53-54,58], no GLP-1 analogues (*n*=2) [14,56], experimental model (*n*=1) [18], meta-analysis (*n*=8) [19,23,31,34-35,41,48,50], commentary (*n*=1) [33].

1. Diet, drugs, and surgery for weight loss. The Medical Letter on Drugs and Therapeutics [Internet]. 2015 Feb 16 [cited 2024 Jul 16];57(1462):21–8. Available from: https://pubmed.ncbi.nlm.nih.gov/25668315/
2. Semaglutide (Ozempic) for Weight Loss | The Medical Letter Inc. [Internet]. The Medical Letter. 2021. Available from: https://secure.medicalletter.org/TML-article-1621c
3. Apovian CM, Garvey WT, Ryan DH. Challenging obesity: Patient, provider, and expert perspectives on the roles of available and emerging nonsurgical therapies. Obesity [Internet]. 2015 Jul;23:S1–26. Available from: https://www.ncbi.nlm.nih.gov/pmc/articles/PMC4699189/
4. Armstrong MJ, Barton D, Gaunt P, Hull D, Guo K, Stocken D, et al. Liraglutide efficacy and action in non-alcoholic steatohepatitis (LEAN): study protocol for a phase II multicentre, double-blinded, randomised, controlled trial. BMJ Open. 2013 Nov;3(11):e003995.
5. Blundell J, Finlayson G, Axelsen M, Flint A, Gibbons C, Kvist T, et al. Effects of once‐weekly semaglutide on appetite, energy intake, control of eating, food preference and body weight in subjects with obesity. Diabetes, Obesity and Metabolism [Internet]. 2017 May 5;19(9):1242–51. Available from: https://www.ncbi.nlm.nih.gov/pmc/articles/PMC5573908/
6. Chatzigeorgiou A, Kandaraki E, Papavassiliou AG, Koutsilieris M. Peripheral targets in obesity treatment: a comprehensive update. Obesity Reviews. 2014 Feb 26;15(6):487–503.
7. Christou GA, Katsiki N, N. Kiortsis D. The Current Role of Liraglutide in the Pharmacotherapy of Obesity. Current Vascular Pharmacology. 2016 Feb 8;14(2):201–7.
8. Clements JN, Shealy KM. Liraglutide. Annals of Pharmacotherapy. 2015 May 18;49(8):938–44.
9. Cornell S, D’Souza J. Pharmacotherapy Considerations in Diabetes and Obesity: Setting Patients up for Success. Postgraduate Medicine. 2014 Mar;126(2):100–9.
10. Coskun T, Urva S, Roell WC, Qu H, Loghin C, Moyers JS, et al. LY3437943, a novel triple glucagon, GIP, and GLP-1 receptor agonist for glycemic control and weight loss: From discovery to clinical proof of concept. Cell Metabolism [Internet]. 2022 Sep 6;34(9):1234-1247.e9. Available from: https://pubmed.ncbi.nlm.nih.gov/35985340/
11. Ebdrup BH, Knop FK, Ishøy PL, Egill Rostrup, Fagerlund B, Lublin H, et al. Glucagon-like peptide-1 analogs against antipsychotic-induced weight gain: potential physiological benefits. BMC medicine [Internet]. 2012 Aug 15;10(1). Available from: https://www.ncbi.nlm.nih.gov/pmc/articles/PMC3573939/
12. Elkind-Hirsch KE, Chappell N, Shaler D, Storment J, Bellanger D. Liraglutide 3 mg on weight, body composition, and hormonal and metabolic parameters in women with obesity and polycystic ovary syndrome: a randomized placebo-controlled-phase 3 study. Fertility and Sterility. 2022 Aug;118(2):371–81.
13. Farr OM, Upadhyay J, Rutagengwa C, DiPrisco B, Ranta Z, Adra A, et al. Longer‐term liraglutide administration at the highest dose approved for obesity increases reward‐related orbitofrontal cortex activation in response to food cues: Implications for plateauing weight loss in response to anti‐obesity therapies. Diabetes, Obesity and Metabolism. 2019 Aug 8;21(11):2459–64.
14. Ferjan S, Janez A, Jensterle M. Dipeptidyl Peptidase-4 Inhibitor Sitagliptin Prevented Weight Regain in Obese Women with Polycystic Ovary Syndrome Previously Treated with Liraglutide: A Pilot Randomized Study. Metabolic Syndrome and Related Disorders. 2017 Dec;15(10):515–20.
15. Lundgren JR, Janus C, Jensen SBK, Juhl CR, Olsen LM, Christensen RM, et al. Healthy Weight Loss Maintenance with Exercise, Liraglutide, or Both Combined. New England Journal of Medicine. 2021 May 6;384(18):1719–30.
16. Gadde KM, Atkins KD. The limits and challenges of antiobesity pharmacotherapy. Expert Opinion on Pharmacotherapy. 2020 Apr 15;21(11):1319–28.
17. Geloneze B, de Lima-Júnior JC, Velloso LA. Glucagon-Like Peptide-1 Receptor Agonists (GLP-1RAs) in the Brain–Adipocyte Axis. Drugs [Internet]. 2017;77(5):493–503. Available from: https://www.ncbi.nlm.nih.gov/pmc/articles/PMC5357258/
18. Guo W, Xu Z, Zou H, Li F, Li Y, Feng J, et al. Discovery of ecnoglutide – A novel, long-acting, cAMP-biased glucagon-like peptide-1 (GLP-1) analog. Molecular Metabolism [Internet]. 2023 Sep 1;75:101762. Available from: https://www.sciencedirect.com/science/article/pii/S2212877823000960
19. Guo X, Zhou Z, Lyu X, Xu H, Zhu H, Pan H, et al. The Antiobesity Effect and Safety of GLP-1 Receptor Agonist in Overweight/Obese Patients Without Diabetes: A Systematic Review and Meta-Analysis. Hormone and Metabolic Research. 2022 May 5;54(07):458–71.
20. Haupt A, Blevins T, Connery L, Rosenstock J, Raha S, Liu R, et al. Daily Oral GLP-1 Receptor Agonist Orforglipron for Adults with Obesity. The New England Journal of Medicine. 2023 Jun 23;389(10).
21. Horowitz M, Aroda VR, Han J, Hardy E, Rayner CK. Upper and/or lower gastrointestinal adverse events with glucagon-like peptide-1 receptor agonists: Incidence and consequences. Diabetes, Obesity and Metabolism. 2017 Feb 17;19(5):672–81.
22. Iacobellis G, Mohseni M, Bianco SD, Banga PK. Liraglutide causes large and rapid epicardial fat reduction. Obesity (Silver Spring, Md) [Internet]. 2017 [cited 2019 Jul 16];25(2):311–6. Available from: https://www.ncbi.nlm.nih.gov/pubmed/28124506
23. Iqbal J, Wu H, Hu N, Zhou Y, Li L, Xiao F, et al. Effect of glucagon‐like peptide‐1 receptor agonists on body weight in adults with obesity without diabetes mellitus—a systematic review and meta‐analysis of randomized control trials. Obesity Reviews. 2022 Feb 22;23(6).
24. Ishøy PL, Knop FK, Broberg BV, Baandrup L, Fagerlund B, Jørgensen NR, et al. Treatment of antipsychotic-associated obesity with a GLP-1 receptor agonist—protocol for an investigator-initiated prospective, randomised, placebo-controlled, double-blinded intervention study: the TAO study protocol. BMJ Open [Internet]. 2014 Jan 1;4(1):e004158. Available from: https://bmjopen.bmj.com/content/4/1/e004158
25. Ishøy PL, Knop FK, Broberg BV, Bak N, Andersen UB, Jørgensen NR, et al. Effect of GLP-1 receptor agonist treatment on body weight in obese antipsychotic-treated patients with schizophrenia: a randomized, placebo-controlled trial. Diabetes, Obesity and Metabolism. 2016 Nov 14;19(2):162–71.
26. Jensen SBK, Lundgren JR, Janus C, Juhl CR, Olsen LM, Rosenkilde M, et al. Protocol for a randomised controlled trial of the combined effects of the GLP-1 receptor agonist liraglutide and exercise on maintenance of weight loss and health after a very low-calorie diet. BMJ Open [Internet]. 2019 Nov;9(11):e031431. Available from: https://bmjopen.bmj.com/content/9/11/e031431
27. Jensterle M, Kravos NA, Pfeifer M, Kocjan T, Janez A. A 12-week treatment with the long-acting glucagon-like peptide 1 receptor agonist liraglutide leads to significant weight loss in a subset of obese women with newly diagnosed polycystic ovary syndrome. Hormones (Athens, Greece) [Internet]. 2015 Jan 1;14(1):81–90. Available from: https://pubmed.ncbi.nlm.nih.gov/25885106/
28. Kalra S, Gupta Y. Endocrine and metabolic effects of Glucagon like peptide 1 receptor agonists (GLP1RA). JPMA The Journal of the Pakistan Medical Association [Internet]. 2016 Mar 1;66(3):357–9. Available from: https://pubmed.ncbi.nlm.nih.gov/26968296/
29. Kelly AS, Metzig AM, Rudser KD, Fitch AK, Fox CK, Nathan BM, et al. Exenatide as a Weight-Loss Therapy in Extreme Pediatric Obesity: A Randomized, Controlled Pilot Study. Obesity. 2012 Feb;20(2):364–70.
30. Kelly AS, Rudser KD, Nathan BM, Fox CK, Metzig AM, Coombes BJ, et al. The Effect of Glucagon-Like Peptide-1 Receptor Agonist Therapy on Body Mass Index in Adolescents With Severe Obesity. JAMA Pediatrics. 2013 Apr 1;167(4):355.
31. Khera R, Murad MH, Chandar AK, Dulai PS, Wang Z, Prokop LJ, et al. Association of Pharmacological Treatments for Obesity With Weight Loss and Adverse Events: A Systematic Review and Meta-analysis. JAMA [Internet]. 2016;315(22):2424–34. Available from: https://www.ncbi.nlm.nih.gov/pubmed/27299618
32. Kim GW, Lin JE, Blomain ES, Waldman SA. Antiobesity Pharmacotherapy: New Drugs and Emerging Targets. Clinical Pharmacology & Therapeutics [Internet]. 2013 Jan [cited 2019 Dec 12];95(1):53–66. Available from: https://pubmed.ncbi.nlm.nih.gov/24105257-antiobesity-pharmacotherapy-new-drugs-and-emerging-targets/
33. Koch L. GLP-1R agonists—the new weapon against obesity? Nature Reviews Endocrinology. 2012 Jan 31;8(4):196–6.
34. Lin Q, Xue Y, Zou H, Ruan Z, Oi C, Hu H. Efficacy and safety of liraglutide for obesity and people who are overweight: a systematic review and meta-analysis of randomized controlled trials. Expert Review of Clinical Pharmacology. 2022 Oct 5;15(12):1461–9.
35. Ma H, Lin YH, Dai LZ, Lin CS, Huang Y, Liu SY. Efficacy and safety of GLP-1 receptor agonists versus SGLT-2 inhibitors in overweight/obese patients with or without diabetes mellitus: a systematic review and network meta-analysis. BMJ Open [Internet]. 2023 Mar 1;13(3):e061807. Available from: https://bmjopen.bmj.com/content/13/3/e061807
36. MacConell L, Remillard C, Li Y, Pencek R, Maggs D, Porter L. Exenatide once weekly: sustained improvement in glycemic control and cardiometabolic measures through 3 years. Diabetes, Metabolic Syndrome and Obesity: Targets and Therapy. 2013 Jan;6:31–41.
37. McGowan BM, Batterham RL, Calanna S, Wilding JPH. Once-Weekly Semaglutide in Adults with Overweight or Obesity. The New England Journal of Medicine [Internet]. 2021 Feb 10;384(11):989–1002. Available from: https://www.nejm.org/doi/full/10.1056/NEJMoa2032183
38. Mehta A, Marso SP, Neeland IJ. Liraglutide for weight management: a critical review of the evidence. Obesity Science & Practice. 2016 Dec 19;3(1):3–14.
39. Millar K, Poole R. Current drugs for weight loss. Practical Diabetes. 2016 Sep;33(7):229–32.
40. Mok J, Adeleke MO, Brown A, Magee CG, Firman C, Makahamadze C, et al. Safety and Efficacy of Liraglutide, 3.0 mg, Once Daily vs Placebo in Patients With Poor Weight Loss Following Metabolic Surgery: The BARI-OPTIMISE Randomized Clinical Trial. JAMA Surgery [Internet]. 2023 Oct 1;158(10):1003–11. Available from: https://jamanetwork.com/journals/jamasurgery/fullarticle/2807724?utm_campaign=articlePDF&utm_medium=articlePDFlink&utm_source=articlePDF&utm_content=jamasurg.2023.2930
41. Monami M, Dicembrini I, Marchionni N, Rotella CM, Mannucci E. Effects of Glucagon-Like Peptide-1 Receptor Agonists on Body Weight: A Meta-Analysis. Experimental Diabetes Research. 2012;2012:1–8.
42. Nathan BM, Rudser KD, Abuzzahab MJ, Fox CK, Coombes BJ, Bomberg EM, et al. Predictors of weight-loss response with glucagon-like peptide-1 receptor agonist treatment among adolescents with severe obesity. Clinical Obesity. 2015 Dec 18;6(1):73–8.
43. O’Neil PM, Garvey WT, Gonzalez-Campoy JM, Mora P, Ortiz RV, Guerrero G, et al. Effects of Liraglutide 3.0 mg on weight and risk factors in Hispanic versus Non-hispanic populations: subgroup analysis from scale randomized trials. Endocrine Practice. 2016 Nov;22(11):1277–87.
44. Overgaard RV, Lindberg SØ, Thielke D. Impact on HbA1c and body weight of switching from other GLP‐1 receptor agonists to semaglutide: A model‐based approach. Diabetes, Obesity & Metabolism [Internet]. 2019 Jan 1;21(1):43–51. Available from: https://www.ncbi.nlm.nih.gov/pmc/articles/PMC6585654/
45. Peradze N, Farr OM, Perakakis N, Lázaro I, Sala-Vila A, Mantzoros CS. Short-term treatment with high dose liraglutide improves lipid and lipoprotein profile and changes hormonal mediators of lipid metabolism in obese patients with no overt type 2 diabetes mellitus: a randomized, placebo-controlled, cross-over, double-blind clinical trial. Cardiovascular Diabetology [Internet]. 2019 Oct 31;18. Available from: https://www.ncbi.nlm.nih.gov/pmc/articles/PMC6823961/
46. Powell AG, Apovian CM, Aronne LJ. New drug targets for the treatment of obesity. Clinical Pharmacology and Therapeutics [Internet]. 2011 Jul 1;90(1):40–51. Available from: https://pubmed.ncbi.nlm.nih.gov/21654742/
47. Rigas G, Bailey TS, Billings LK, Davies M, Frias JP, Koroleva A, et al. Effect of Subcutaneous Semaglutide vs Placebo as an Adjunct to Intensive Behavioral Therapy on Body Weight in Adults With Overweight or Obesity: The STEP 3 Randomized Clinical Trial. JAMA [Internet]. 2021 Apr 13;325(14):1403–13. Available from: https://jamanetwork.com/journals/jama/fullarticle/2777025
48. Robert SA, Rohana AG, Shah SA, Chinna K, Wan Mohamud WN, Kamaruddin NA. Improvement in binge eating in non-diabetic obese individuals after 3 months of treatment with liraglutide – A pilot study. Obesity Research & Clinical Practice. 2015 May;9(3):301–4.
49. Rosenstock J, Klaff LJ, Schwartz S, Northrup J, Holcombe JH, Wilhelm K, et al. Effects of Exenatide and Lifestyle Modification on Body Weight and Glucose Tolerance in Obese Subjects With and Without Pre-Diabetes. Diabetes Care. 2010 Mar 23;33(6):1173–5.
50. Ryan PM, Seltzer S, Hayward NE, Rodriguez DA, Sless RT, Hawkes CP. Safety and Efficacy of Glucagon-Like Peptide-1 Receptor Agonists in Children and Adolescents with Obesity: A Meta-Analysis. The Journal of Pediatrics. 2021 Sep;236:137-147.e13.
51. Ryder B, McKnight J, Blann A, Dhatariya K, Gregory R, Robinson T, et al. ABCD position statement on GLP-1 based therapies and pancreatic damage. Practical Diabetes. 2013 Nov;30(9):388–91.
52. Schou M, Batterham RL, Bhatta M, Buscemi S, Christensen LN, Frias JP, et al. Two-year effects of semaglutide in adults with overweight or obesity: the STEP 5 trial. Nature Medicine [Internet]. 2022 Oct 1;28(10):2083–91. Available from: https://www.nature.com/articles/s41591-022-02026-4
53. Steurer, J. (2022). Weight loss with semaglutide is significantly greater than with liraglutide. Praxis **111**(9): 534-535.
54. ten Kulve JS, Veltman DJ, van Bloemendaal L, Groot PFC, Ruhé HG, Barkhof F, et al. Endogenous GLP1 and GLP1 analogue alter CNS responses to palatable food consumption. Journal of Endocrinology. 2016 Apr;229(1):1–12.
55. Thakur U, Bhansali A, Gupta R, Rastogi A. Liraglutide Augments Weight Loss After Laparoscopic Sleeve Gastrectomy: a Randomised, Double-Blind, Placebo-Control Study. Obesity Surgery. 2020 Jul 12;31(1).
56. Tronieri JS, Alfaris N, Chao AM, Pearl RL, Alamuddin N, Bakizada ZM, et al. Lorcaserin plus lifestyle modification for weight loss maintenance: Rationale and design for a randomized controlled trial. Contemporary Clinical Trials. 2017 Aug;59:105–12.
57. Tronieri JS, Wadden TA, Walsh OA, Berkowitz RI, Alamuddin N, Gruber K, et al. Effects of liraglutide plus phentermine in adults with obesity following 1 year of treatment by liraglutide alone: A randomized placebo-controlled pilot trial. Metabolism. 2019 Jul;96:83–91.
58. Updike WH, Pane O, Franks R, Saber F, Abdeen F, Balazy DD, et al. Is it Time to Expand Glucagon-like Peptide-1 Receptor Agonist Use for Weight Loss in Patients Without Diabetes? Drugs. 2021 Apr 30;81(8):881–93.
59. Weghuber D, Barrett T, Barrientos-Pérez M, Gies I, Hesse D, Jeppesen OK, et al. Once-Weekly Semaglutide in Adolescents with Obesity. New England Journal of Medicine [Internet]. 2022 Nov 2;387(24):2245–57. Available from: https://www.nejm.org/doi/full/10.1056/NEJMoa2208601
60. Wilding JPH, Batterham RL, Calanna S. Once-Weekly Semaglutide in Adults with Overweight or Obesity. The New England Journal of Medicine [Internet]. 2021 Feb 10;384(11):989–1002. Available from: https://www.nejm.org/doi/full/10.1056/NEJMoa2032183
61. Wilding JPH, Rajeev SP, DeFronzo RA. Positioning SGLT2 Inhibitors/Incretin-Based Therapies in the Treatment Algorithm. Diabetes Care. 2016 Jul 19;39(Supplement 2):S154–64.
62. Wilding JPH, Calanna S, Davies M, Dicker D, Garvey WT, Goldman B, et al. Semaglutide 2.4 mg for the Treatment of Obesity: Key Elements of the STEP Trials 1 to 5. Obesity. 2020 May 22;28(6):1050–61.
63. Zhang F, Tong Y, Su N, Li Y, Tang L, Huang L, et al. Weight loss effect of glucagon-like peptide-1 mimetics on obese/overweight adults without diabetes: A systematic review and meta-analysis of randomized controlled trials胰高血糖素样肽-1类似物对非糖尿病肥胖/超重成人的减重作用：一篇对随机对照试验进行的系统评价及meta分析. Journal of Diabetes. 2014 Sep 10;7(3):329–39.
