# Supplementary material for: Gastrointestinal adverse events associated with GLP-1 RA in non-diabetic patients with overweight or obesity: a systematic review and network meta-analysis
Source: Int J Obes (Lond). 2025 Aug 13;49(10):1946–57. doi: 10.1038/s41366-025-01859-6 (PMC12532569; doi:10.1038/s41366-025-01859-6)
Supplement: Supplementary file 3 — Studies evaluating GI adverse events with GLP-1 agonists in subjects with overweight and obesity [file 41366_2025_1859_MOESM3_ESM.docx]

**Supplementary Table 1.** Studies evaluating GI adverse events with GLP-1 agonists in subjects with overweight and obesity

| First Author / Year / Country | Study Design | Study Characteristics | Main Findings |
| --- | --- | --- | --- |
| Elkind-Hersch A et al. /  2008 / USA | Open-label prospective, randomized, outpatient clinical  trial with three treatment groups | - **Population**: Non-diabetic females with obesity and PCOS - **Total subjects**: 60 patients enrolled, 41 completed - **BMI**: 39.9±1.5 (EX), 41.3±1.8 (MET), 41.2±1.7 (COM) - **Mean age (years):** 28.2±1.1 (EX), 27.7±1.3 (MET), 32.1±0.7 (COM) - **Sex:** Females: 60 (100%) - **Intervention: EX vs. MET vs. COM.** - **Duration:** An open-label prospective, randomized, outpatient clinical trial with three treatment groups for 24 weeks - **GI adverse events: n = 34** - Nausea: n = 16 - Diarrhea: n = 8 - Bloating: n = 3 - Vomiting: n = 1 - Cramping (gastrointestinal): n = 1 - Indigestion/heartburn: n = 2 - Stomachache: n = 1   Constipation: n = 2 | The most frequent adverse events were mild or moderate and were gastrointestinal in nature. Nausea was the most frequent adverse event (overall 27%), and it was higher in COM- treated subjects than in those on monotherapy. Nausea was generally mild or moderate in intensity and was reported at a higher incidence during the initial weeks of EX therapy (weeks 0 – 8) and declined thereafter. Other reported adverse events associated with EX were vomiting (7%) or headache (2%). Diarrhea (overall 13%) was more likely to occur with combination or MET treatment. |
| Astrup A et al. /  2009 / Denmark | Randomized, double-blind, placebo-controlled study | - **Population**: Non-diabetic participants with obesity of stable weight - **Total subjects**: 564 enrolled, 472 completed - **BMI**: 35 - **Mean age (years):** 46 - **Sex:** Female: 75%, Male: 25% - **Intervention:** Individuals were randomly assigned to one of four liraglutide doses (1·2 mg, 1·8 mg, 2·4 mg, or 3·0 mg) or to placebo administered once a day subcutaneously, or orlistat (120 mg) three times a day orally. - **Duration:** randomized, double-blind, placebo-controlled study for 20 weeks - **GI adverse events: n = 315** - Constipation: n =71 - Diarrohea: n= 72 - Nausea: n= 138 - Vomiting: n = 40 | Nausea and vomiting were more frequent with liraglutide than with the other treatments, although these events were mostly transient and of mild or moderate intensity. |
| Dushay J et al. /  2011 / USA | Randomized, double-blind, placebo controlled,  crossover study | - **Population**: Non-diabetic females with obesity and PCOS - **Total subjects**: 41 patients - **BMI**: 33±4 - **Mean age (years):** 48±11 - **Sex:** Females: 41 (100%) - **Intervention:** Exenatide vs. placebo. There was no lifestyle intervention. - **Duration:** 35-week randomized, double-blind, placebo controlled, crossover study, including two 16-week treatment periods separated by a 3-week washout period. - **GI adverse events: n = 29** - Nausea: n = 23 - Diarrhea: n = 1 - Constipation: n = 2 - Heartburn: n = 1 - Belching: n = 1 - Uncomfortable satiety: n = 1 | Nausea scores were not significantly different between exenatide and placebo. |
| Sze L et al. / 2011 / Australia | Single-blinded, randomized, crossover trial | - **Population**: Non-diabetic participants with obesity - **Total subjects**: 11 enrolled - **BMI**: 34.4±1.3 - **Mean age (years):** 31.3±2.7 - **Sex:** Male 6 (60%), female 5 (40%) - **Intervention:** participants received either 10 g exenatide or normal saline injected sc 15 min before a standardized breakfast - **Duration:** N.A. - **GI adverse events: 13** - Nausea: n = 5 - Bloating: n = 6 - Vomiting: n = 2 | No side effects were observed in PWS subjects, with placebo or exenatide, whereas nine of 11 subjects with obesity (*P <* 0.001) reported side effects with exenatide, most commonly bloating (n = 6), nausea (n = 5), and vomiting (n = 2). |
| Astrup A et al. /  2012 / Denmark | Randomized, double-blind, placebo-controlled study | - **Population**: Non-diabetic participants of stable weight and fasting plasma glucose (FPG) <7 mmol l^-1^ (126 mg dl) at run-in - **Total subjects**: 564 adults enrolled, 398 entered the extension and 268 completed the 2-year trial - **BMI**: N/A, ≥30 and ≤40 kg/m^2^ - **Mean age (years):** N/A, 18-65 years - **Sex:** N/A - **Intervention:** Participants received diet (500 kcal deficit per day) and exercise counseling during 2-week run-in, before being randomly assigned (with a telephone or web-based system) to once-daily subcutaneous liraglutide (1.2, 1.8, 2.4 or 3.0 mg), placebo, or open-label orlistat. After 1 year, liraglutide/placebo recipients switched to liraglutide 2.4 mg, then 3.0 mg - **Duration:** A randomized, double-blind, placebo-controlled 20-week study with 2-year extension (sponsor unblinded at 20 weeks, - participants/investigators at 1 year) - **GI adverse events: n = 546** - Abdominal pain: n = 11 - Abdominal pain upper: n = 21 - Constipation: n = 71 - Diarrhea: n = 53 - Dyspepsia: n = 35 - Flatulence: n = 11 - Nausea: n = 176 - Toothache: n = 9 - Vomiting: n = 57 | Nausea and vomiting were more frequent with liraglutide than with the other treatments, although these events were mostly transient and of mild or moderate intensity. |
| Jensterle M et al. /  2013 / Slovenia | Open-label  prospective, randomized, outpatient clinical study | - **Population**: Non-diabetic females with obesity and PCOS - **Total subjects**: 40 enrolled, 35 completed - **BMI**: 36.6±3.5 (metformin), 39.3±4.2 (liraglutide), 37.6±5.1 (combination) - **Mean age (years):** 31.3±9.4 (metformin), 31.5±6.4 (liraglutide), 31.1±5.1 (combination) - **Sex:** Female: 40 (100%) - **Intervention:** Metformin vs. liraglutide vs. combination. There was no lifestyle intervention. - **Duration:** an open-label prospective, randomized, outpatient clinical study for 12 weeks - **GI adverse events: n = 12** - Nausea: n = 6 - Diarrhea: n = 6 | The most frequent adverse events were nausea and diarrhea that were generally mild to moderate, subsided over time and did not correlate with weight loss. |
| Lean M E et al. / 2014 / UK | Randomized, placebo-controlled, double-blind study | - **Population**: Non-diabetic participants with obesity - **Total subjects**: 564 received randomized treatment, 472 completed the 20-week trial and 398 chose to enroll in the extension period (74 discontinued), with 268 completing the full 2-year period - **BMI**: 34.8±2.7 - **Mean age (years):** 45.9±10.3 - **Sex:** 135 male (24%), 429 female (76%) - **Intervention:** After the run-in period, individuals were randomly assigned to double- blinded treatment (liraglutide, placebo or orlistat). Liraglutide doses of 1.2, 1.8, 2.4 or 3.0 mg were administered once daily by evening subcutaneous injection using a pre-filled injection pen with NovoFine Needles 8 mm 30 G, starting with doses of 0.6mg per day and increasing by weekly increments of 0.6 mg (dose escalation). The open-label comparator group was randomized to receive orlistat capsules (3 120 mg per day) with each main meal for the full 2-year period. During the run-in period and throughout treatment, all participants received dietary counseling for a nutritionally balanced low-calorie diet (with about 30% of total caloric intake from fat, 20% from protein and 50% from carbohydrates), providing an energy deficit of approximately 500 kcal per day below the estimated 24-h energy requirements (calculated as basal metabolic rate physical activity level 1.3). Participants were also advised to maintain or increase physical activity. - **Duration:** randomized, placebo-controlled, double-blind 20-week study with an 84-week extension - **GI adverse events: 442** - Nausea: n = 386 - Vomiting: n = 56 | The frequency of nausea and/or vomiting for women (n1⁄4135; 19% (95% confidence interval (CI) 13–26%)) was not statistically significantly different from that of men (n1⁄429; 12% (95% CI 7–20%)): the odds ratio (female: male) was 1.7 (95% CI: 0.9–3.1; P 1⁄4 0.09). The proportion of individuals reporting nausea/vomiting at any time during year 1 on liraglutide was dose-dependent, and statistically significantly greater than with placebo and orlistat for all liraglutide doses. |
| Wadden T A et al. / 2013 / USA | Randomized, double-blind, placebo- controlled trial | - **Population**: Non-diabetic participants with obesity/overweight - **Total subjects**: 422 enrolled - **BMI**: 36 (liraglutide 3.0 mg), 35.2 (placebo) - **Mean age (years):** 45.9 (liraglutide 3.0 mg), 46.5 (placebo) - **Sex:** male/female 16/84 (liraglutide 3.0 mg), 21/79 (placebo) - **Intervention:** lost ≥5% of initial weight during an LCD run-in were randomly assigned to liraglutide 3.0 mg per day or placebo (subcutaneous administration) - **Duration:** randomized, double-blind, placebo- controlled trial for 56 weeks - **GI adverse events: 156** - Nausea: n = 101 - Constipation: n = 57 - Diarrhea: n = 38 - Vomiting: n = 35 - Dyspepsia: n = 20 - Abdominal pain: n = 14 - Abdominal distention: n = 13 - Eructation: n = 11 - Flatulence: n = 11 | In the liraglutide group, most adverse events withdrawals (11/18) were due to GI disorders. Eight of the 11 participants withdrew due to GI events that had onset in the first 4 weeks of the trial during dose escalation. Most GI disorders (94.8%) with liraglutide were of mild or moderate severity. Nausea was transient, with most incidents occurring during the first 4 weeks of treatment, coinciding with dose escalation. |
| Faurschou A et al. /  2014 / Denmark | Randomized placebo-controlled trial | - **Population**: Non-diabetic patients with obesity and plaque psoriasis - **Total subjects**: 21 enrolled, 20 completed - **BMI**: 35 (placebo), 37 (liraglutide) - **Mean age (years):** 48 (placebo), 52 (liraglutide) - **Sex:** Female: 6 (29%), Male: 15 (71%) - **Intervention:** Liraglutide vs. placebo. There was no lifestyle intervention. - **Duration:** A randomized placebo-controlled trial for 8 weeks - **GI adverse events: n = 8** - Nausea: n = 5 - Loss of appetite: n = 2 - Constipation: n = 1 | The most common complaint was nausea reported from five patients in the liraglutide group (45%) but from no patients in the control group. Other adverse events included transient GI symptoms such as loss of appetite (18%) and constipation (9%). |
| Iepsen EW et al. /  2014 / Denmark | Randomized controlled trial | - **Population**: Non-diabetic participants with obesity - **Total subjects**: 58 enrolled, 52 completed - **BMI**: 30.8± 0.7 (liraglutide), 29.7±0.7 (control) - **Mean age (years):** 46 ± 2 (liraglutide), 45 ± 2 (control) - **Sex:** Female: 44 (85%), male: 8 (15%) - **Intervention:** Liraglutide vs. control. There was no lifestyle intervention. - **Duration:** A randomized controlled trial for 8 weeks - **GI adverse events: n = 11** - Nausea: n = 11 | Mild-to-moderate GI side effects are common but transient during treatment with exenatide once weekly. |
| Van can J et al / 2014 / Netherlands | Randomized, placebo-controlled, double-blind, two-period incomplete crossover trial | - **Population**: Non-diabetic participants with obesity - **Total subjects**: 49 enrolled - **BMI**: 34.2±2.7 - **Mean age (years):** 48.3±13.2 - **Sex:** male 29 (59%), female 21 (41%) - **Intervention:** liraglutide 1.8mg, 3.0mg and placebo were administered once daily by evening subcutaneous injections - **Duration:** randomized, placebo-controlled, double-blind, two-period incomplete crossover trial for 20 weeks. - **GI adverse events: 77** - Nausea: n = 20 - Diarrhea: n = 15 - Feces hard: n = 5 - Gastroesophageal reflux disease: n = 9 - Abdominal pain upper: n = 3 - Constipation: n = 5 - Eructation: n = 2 - Infrequent bowel movement: n = 2 - Vomiting: n = 6 | The proportion of individuals reporting adverse effects, which were all of mild or moderate severity, was similar for liraglutide 1.8 mg (90%) and 3.0 mg (94%), and lower (75%) for placebo. |
| Jensterle M et al. /  2015 / Slovenia | Prospective randomized open-label study | - **Population**: Non-diabetic females with obesity and PCOS - **Total subjects**: 45 enrolled, 41 completed - **BMI**: 30.7 ± 7.9 - **Mean age (years):** 38.6 ±6.0 - **Sex:** Female: 45 (100%) - **Intervention:** Metformin vs. liraglutide vs. roflumilast. There was no lifestyle intervention. - **Duration:** A prospective randomized open-label study for 12 weeks - **GI adverse events: n = 6** - Nausea: n = 4 - Obstipation: n = 2 - Diarrhea: n = 1 | Nausea in LIRA arm was present up to 3 days when liraglutide was initiated at a dose of 0.6 mg injected s.c. once per day and if present reappeared for 2 to 3 days when the dose was increased to 1.2 mg/day after 1 week. It was not accompanied with vomiting. Nausea in ROF arm was more persistent when compared to MET yet it was mild and not accompanied with vomiting. |
| Pi-Sunyer X et al. / 2015 / USA | Double-blind trial | - **Population**: Non-diabetic patients with overweight or obesity and dyslipidemia or hypertension - **Total subjects**: 3731 enrolled, 3662 completed - **BMI**: 38.3±6.4 (liraglutide), 38.3±6.3 (placebo) - **Mean age (years):** 45.2±12.1 (liraglutide), 45±12 (placebo) - **Sex:** female 1957 (78.7%) and male 530 (21.3%) (liraglutide), female 971 (78.1%) and male 273 (21.9%) (placebo) - **Intervention:** patients were randomly assigned in a 2:1 ratio to receive once-daily subcutaneous injections of liraglutide at a dose of 3.0 mg (2487 patients) or placebo (1244 patients); both groups received counseling on lifestyle modification - **Duration:** double-blind trial for 56 weeks - **GI adverse events: 2,941** - Nausea: n = 997 - Diarrhea: n = 518 - Constipation: n = 495 - Vomiting: n = 404 - Dyspepsia: n = 236 - Upper abdominal pain: n = 141 - Abdominal pain: n = 130   *Cholelithiasis: n = 20 | GI disorders are common and mostly transient side effects of treatment. |
| Blackman A et al. /  2016 / Canada | Randomized, double-blind trial | - **Population**: Non-diabetic participants with obesity and moderate (apnea–hypopnea index (AHI) 15.0–29.9 events h − 1) or severe (AHI ⩾30.0 events h − 1) OSA and are unable or unwilling to use CPAP therapy - **Total subjects**: 359 enrolled, 142 completed - **BMI**: 38.9±6.4 (liraglutide 3.0 mg), 39.4±7.4 (placebo) - **Mean age (years):** 48.6±9.9 (liraglutide 3.0 mg), 48.4±9.5 (placebo) - **Sex:** Females: 101 (28%), Males: 258 (72%) - **Intervention:** Participants were randomized for 32 weeks to liraglutide 3.0 mg or placebo, both as adjunct to diet (500 kcal day − 1 - deficit) and exercise - **Duration:** A randomized, double-blind trial for 32 weeks - **GI adverse events: n = 182** - Nausea: n = 59 - Diarrhea: n = 38 - Constipation: n = 26 - Dyspepsia: n = 20 - Vomiting: n = 16 - GERD: n = 11 - Lipase increased: n = 11 - Cholelithiasis: n = 1 | GI events, consistent with the known physiological effects of GLP-1, were more frequent than with placebo. At year 1, nausea and/or vomiting was associated with greater weight loss with liraglutide 3.0mg, but even those who did not experience these events lost more weight than those on placebo or orlistat. |
| Jensterle M et al. /  2016 / Slovenia | Prospective randomized open-label design | - **Population**: Non-diabetic females with obesity and PCOS - **Total subjects**: 44 enrolled, 43 completed - **BMI**: 37.2±4.5 - **Mean age (years):** 30.3±4.4 - **Sex:** Female: 44 (100%) - **Intervention:** Liraglutide vs. combined liraglutide and metformin. There was no lifestyle intervention. - **Duration:** A prospective randomized open-label design for 12 weeks - **GI adverse events: n = 13** - Nausea: n = 6 - Diarrhea: n = 6 - Vomiting: n = 1 | All side effects in LIRA group resolved within the first 4 weeks. Adverse events associated with COMBI treatment were present in the two months of treatment. |
| Halawi H et al. /  2017 / USA | Randomized, double-blind, placebo-controlled pilot trial | - **Population**: Non-diabetic or over-weight participants with obesity and at least one weight-related comorbidity - **Total subjects**: 47 enrolled, 35 completed - **BMI**: N/A - **Mean age (years):** 37 (26–51) (placebo), 42 (32–51) (liraglutide) - **Sex:** N/A - **Intervention:** Liraglutide vs. placebo. There was no lifestyle intervention. - **Duration:** A randomized, double-blind, placebo-controlled pilot trial for 16 weeks - **GI adverse events: n = 3** - Nausea: n = 12 - Uneventful cholecystectomy for acute cholecystitis: n = 1 | A post-hoc subgroup analysis of the liraglutide group comparing the participants who reported nausea at any time while on liraglutide (n=12) with those who did not experience nausea (n=6) showed no significant difference in the median gastric emptying T1/2 at baseline, 5 weeks, or 16 weeks between the two groups. |
| Liu X et al. /  2017 / China | Open-label prospective, randomized, clinical study | - **Population**: Non-diabetic females with overweight/obesity and PCOS - **Total subjects**: 175 enrolled, 158 completed - **BMI**: 28.29±1.86 (metformin), 29.16±3.11 (exenatide) - **Mean age (years):** 27.69±3.80 (metformin), 27.93±2.70 (exenatide) - **Sex:** Female: 176 (100%) - **Intervention:** Metformin vs exenatide - **Duration:** 24-week open-label prospective, randomized, clinical study - **GI adverse events: n = 24** - Nausea: n = 14 - Bloating: n = 4 - Vomiting: n = 6 | Besides nausea which was more frequent in the GLP-1RA group (40 vs 4%), there was no difference in the occurrence of adverse effects. The nausea was mild and transient and disappeared after a few weeks. |
| Lundkvist P et al. / 2017 / Sweden | Randomized, parallel-group, double-blind, placebo-controlled trial | - **Population**: Non-diabetic participants with obesity - **Total subjects**: 50 - **BMI**: 35.4 - **Mean age (years):** 51.4 - **Sex:** 40% male, 60% female - **Intervention:** following the 24-week double-blind phase, all eligible participants receiving PBO and DAPA+ExQW were offered 28 weeks of open-label DAPA+ExQW treatment (hereafter referred to as PBO→DAPA+ExQW and continued DAPA+ExQW groups, respectively). - **Duration:** 24-week, randomized, parallel-group, double-blind, placebo-controlled trial followed by an optional 28-week open- label phase - **GI adverse events: 111** - Nausea: n = 26 - Decreased appetite: n = 21 - Abdominal pain: n = 14 - Diarrhea: n = 13 - Abdominal distension: n = 11 - Vomiting: n = 11 - Constipation: n = 9 - Dyspepsia: n = 6 | Nausea was more frequent with dapagliflozin+exenatide than with placebo and diminished over time. No clear difference in adverse event-related withdrawals between placebo and active treatment periods was observed. |
| Nylander M et al. / 2017/ Denmark | Randomized, placebo-controlled, double-blind clinical trial | - **Population**: Non-diabetic females with overweight and PCOS - **Total subjects**: 72 enrolled, 65 completed - **BMI**: 33.3 (liraglutide), 33.3 (placebo) - **Mean age (years):** 31.4 (liraglutide), 26.2 (placebo) - **Sex:** 65 female (100%) - **Intervention:** Liraglutide/placebo was administered as a subcutaneous injection once daily: 0.6 mg the first week, 1.2 mg the second week and 1.8 mg for the rest of the study period. The participants were randomized in a 2:1 ratio (liraglutide:placebo). - **Duration:** randomized, placebo-controlled, double-blind clinical trial for 26 weeks - **GI adverse events: 69** - Nausea: n = 37 - Vomiting: n = 5 - Ructus/heartburn: n = 8 - Diarrhea: n = 5 - Constipation: n = 12 - Epigastrial pain: n = 8   *Gastroenteritis: n = 5 | In the liraglutide group the most prevalent adverse effect was nausea, mainly in the up-titration phase. |
| Zheng S. et al / 2017 / China | Open-label, randomized, prospective trial | - **Population**: Non-diabetic females with overweight/obesity and PCOS - **Total subjects**: 82 enrolled, 64 completed - **BMI**: 29.18 ± 4.15 (EXE), 29.00 ± 4.10 (MET) - **Mean age (years):** 27.70 ± 3.41 (EXE), 28.16 ± 3.92 (MET) - **Sex:** female 82 (100%) - **Intervention:** patients were provided diet and exercise instruction, and all received either subcutaneous exenatide (EXE) 10 mg twice daily or metformin (MET) 1000 mg twice daily - **Duration:** open-label, randomized, prospective trial for 12 weeks - **GI adverse events: 25** - Nausea: n = 9 - Diarrhea: n = 2 - Bloating: n = 2 - Vomiting: n = 6 - Gastrointestinal spasms: n = 2 - Stomachache: n = 2 - Constipation: n = 2 | The prevailing adverse side-effect was a mild or moderate GI reaction. Nausea was the most common reaction (10.98%, 9/82), and the incidence in the EXE group (14.63%, 6/41) was twice as frequent to that in the MET group (7.32%, 3/41). Nausea was generally mild or of moderate intensity, and most symptoms could be seen at 0-8 weeks at the beginning of exenatide treatment; slowly over time, then, the exenatide appeared to be tolerated. Vomiting (9.76%, 4/41) and abdominal distension (4.88%, 2/41) were also observed with exenatide treatment. Use of slowly increasing doses appeared to reduce the exenatide- or metformin-associated GI reactions. |
| Frossing S et al. /  2018 / Denmark | Double-blind, placebo-controlled,  randomized clinical trial | - **Population**: Non-diabetic females with obesity and PCOS - **Total subjects**: 74 enrolled, 65 completed - **BMI**: 33.3±5.1 (liraglutide), 33.3±4.6 (placebo) - **Mean age (years):** N/A - **Sex:** Female: 74 (100%) - **Intervention:** Liraglutide vs. placebo. There was no lifestyle intervention. - **Duration:** A double-blind, placebo-controlled, randomized clinical trial for 24 weeks - **GI adverse events: n = 6** - Nausea: n = 38 - Constipation: n = 13 - Gallstone-related pain: n = 3 - Cholecystectomy: n = 2 | Nausea (liraglutide, 79%; placebo, 13%; P < .01) and constipation (liraglutide, 26%; placebo, 0%; P < .01) were the most prevalent adverse effects. Gallstone-related pain occurred in 6% of participants in the liraglutide group and 4% in the placebo group (P = NS). |
| O’ Neil P M et al. / 2018 / USA | Randomised, double-blind, placebo and active controlled, multicentre, dose-ranging, phase 2 trial | - **Population**: Non-diabetic participants with obesity - **Total subjects**: 957 enrolled, 767 completed - **BMI**: 39.3 (semaglutide), 38.6 (liraglutide), 40.1 (placebo) - **Mean age (years):** 46 (semaglutide), 49 (liraglutide), 46 (placebo) - **Sex:** Male 338 (35%), female 619 (65%) - **Intervention:** randomly assigned participants (6:1) to each active treatment group (ie, semaglutide [0·05 mg, 0·1 mg, 0·2 mg, 0·3 mg, or 0·4 mg; initiated at 0·05 mg per day and incrementally escalated every 4 weeks] or liraglutide [3·0 mg; initiated at 0·6 mg per day and escalated by 0·6 mg per week]) or matching placebo group (equal injection volume and escalation schedule to active treatment group) using a block size of 56. All treatment doses were delivered once-daily via subcutaneous injections. - **Duration:** randomised, double-blind, placebo and active controlled, multicentre, dose-ranging, phase 2 trial for 59 weeks - **GI adverse events: 1,734** - Nausea: n = 362 - Diarrhea: n = 231 - Constipation: n = 175 - Vomiting: n = 134 - Decreased appetite: n = 115 - Eructation: n = 74   *GI disorders: n = 603  *Gallbladder-related disorders: n = 25   - *Hepatic disorders: n = 15 | GI events were semaglutide dose-dependent and numerically more common at the highest dose than on liraglutide 3·0 mg, although the proportion of discontinuations for GI events on any active treatment (3–13% across groups) was low compared with the overall incidence of GI (62–82%). Gallbladder disorders showed a possible dose-related trend for semaglutide, but the number of events was low in all groups and exceeded placebo only at the highest doses. |
| Salamun V et al. / 2018/ Slovenia | prospective randomized open-label study | - **Population**: Infertile non-diabetic females with obesity and PCOS - **Total subjects**: 28 - **BMI**: 35.5±4.9 (metformin), 37.8±3.0 (metformin+liraglutide) - **Mean age (years):** 31.1±4.7 (metformin), 30.1±3.6 (metformin+liraglutide) - **Sex:** female 28 (100%) - **Intervention:** metformin 1000 mg BID or metformin 1000 mg BID combined with 1.2 mg liraglutide QD s.c. - **Duration:** prospective randomized open-label study for 12 weeks - **GI adverse events: 7** - Nausea: n = 6 - Diarrhea: n = 1 | The adverse events were transient and mild. The patients in the MET group reported nausea (1/14) and diarrhea (1/14). The patients in the COMBI group reported nausea (5/13) and headache (2/13). Low-dose and gradual titration reduced the GI side effects associated with liraglutide. |
| Ma R L et al. / 2021 / China | open-label prospective, randomized, outpatient clinical trial | - **Population**: Non-diabetic females with obesity and PCOS - **Total subjects**: 58 enrolled, 40 completed - **BMI**: 30.62±3.42 (metformin), 31.51±4.20 (exenatide+metformin) - **Mean age (years):** 28.17±4.40 (metformin), 30.10±4.52 (exenatide+metformin) - **Sex:** Female 40 (100%) - **Intervention:** Participants were randomly distributed into one of two treatment arms: (1) MET 500 mg three times a day combined with exenatide QW 2mg combination treatment (COM) or (2) MET alone for 12 weeks. MET was administered at an initial dose of 500 mg/day and gradually increased to a final dose of 1500 mg/day within 2 weeks. Patients in both groups were treated with Diane- 35 (ethinylestradiol 0.035 mg and cyproterone acetate 2mg, Bayer, Leverkusen, Germany) for 21 consecutive days from the first day of menstruation or progesterone withdrawal hemorrhage, and the next cycle began after 7 days of withdrawal. - **Duration:** open-label prospective, randomized, outpatient clinical trial for 12 weeks - **GI adverse events: 57** - Nausea: n = 21 - Diarrhea: n = 20 - Bloating: n = 8 - Vomiting: n = 5 - Constipation: n = 3 | Mild and moderate nausea and diarrhea were the most common side effects in both groups, with similar incidence. GI adverse events usually occurred within the first 8 weeks of therapy. |
| Alba M et al. /  2021 / USA | Randomized, double-blind, placebo-controlled and  open-label active-controlled, parallel-group, multicentre study | - **Population**: Non-diabetic participants with obesity and stable weight - **Total subjects**: 474 enrolled, 343 completed - **BMI**: 40 - **Mean age (years):** 46 - **Sex:** Female: 356 (75.1%), Male: 118 (24.9%) - **Intervention:** 1:1:2:2:2 ratio to blinded once-weekly treatment with placebo; JNJ-64565111 5.0 mg, 7.4 mg or 10.0 mg; or open-label once-daily liraglutide 3.0 mg, each with no dose escalation   **Duration:** A randomized, double-blind, placebo-controlled and open-label active-controlled, parallel-group, multicentre study for 26 weeks.   - **GI adverse events: n = 742** - Nausea: n = 237 - Vomiting: n = 144 - Diarrhea: n = 82 - Constipation: n = 68 - Dyspepsia: n = 45 - GERD: n = 39 - Eructation: n = 39 - Abdominal distention: n = 23 - Abdominal pain upper: n = 23 - Dry mouth: n = 18 - Abdominal pain: n = 17   Abdominal discomfort: n = 7 | In the JNJ-64565111 5.0 mg, 7.4 mg and 10.0 mg groups, 6.8%, 13.6% and 22.9%, respectively, discontinued treatment due to an adverse event of nausea compared with 0% and 6.7% in the placebo and liraglutide groups, respectively. |
| Enebo L B et al. /  2021 / USA | Randomized, placebo-controlled, multiple-ascending dose, phase 1b trial | - **Population**: Non-diabetic participants with obesity - **Total subjects**: 96 enrolled, 80 completed - **BMI**: 32±1 - **Mean age (years):** 40±6 - **Sex:** Female: 39 (41%), Male: 56 (59%) - **Intervention:** six sequential overlapping cohorts, and in each cohort eligible participants were randomly assigned (3:1) to once-weekly subcutaneous cagrilintide (0·16, 0·30, 0·60, 1·2, 2·4, or 4·5 mg) or matched placebo, in combination with once-weekly subcutaneous semaglutide 2·4 mg, without lifestyle interventions - **Duration:** A randomized, placebo-controlled, multiple-ascending dose, phase 1b trial for 20 weeks - **GI adverse events: n = 65** - Nausea: n = 9 - Decreased appetite: n = 14 - Early satiety: n = 9 - Vomiting: n = 5 - Dyspepsia: n = 12 - Diarrhea: n = 14   Abdominal pain: n = 2 | The addition of cagrilintide to semaglutide was associated with increased nausea and vomiting, but these events were mild or moderate in severity and the proportion of participants reporting gastrointestinal disorders did not increase with cagrilintide dose. |
| Gudbergsen H et al. /  2021 / Denmark | Randomized controlled trial | - **Population**: Non-diabetic participants with overweight and KOA - **Total subjects**: 168 enrolled, 129 completed - **BMI**: 32.1 - **Mean age (years):** 59.2 - **Sex:** Female: 109 (65%), Male: 59 (35%) - **Intervention:** Liraglutide vs. placebo. There was no lifestyle intervention. - **Duration:** A randomized controlled trial for 52 weeks - **GI adverse events: n = 264**   Gastrointestinal: n = 264 | Patients receiving liraglutide reporting GI adverse events more frequently than patients receiving liraglutide placebo. |
| Lau D C W et al. / 2021 / Canada | randomised, double-blind, placebo-controlled and active-controlled, dose- finding phase 2 trial | - **Population**: Non-diabetic participants with overweight or obesity and hypertension or dyslipidemia - **Total subjects**: 706 enrolled, 677 completed - **BMI**: 37.8 (cagrilintide), 38.4 (liraglutide), 37.4 (placebo) - **Mean age (years):** 52.3 (cagrilintide), 51.5 (liraglutide), 51.4 (placebo) - **Sex:** Male 270 (38%), 436 (62%) - **Intervention:** participants were randomly assigned (6:1) to subcutaneous self-injections of once-weekly cagrilintide (0·3, 0·6, 1·2, 2·4, or 4·5 mg), once-daily liraglutide 3·0 mg, or volume-matched placebo (for six placebo groups). - **Duration:** randomised, double-blind, placebo-controlled and active-controlled, dose- finding phase 2 trial for 32 weeks - **GI adverse events: n = 509** - Nausea: n = 202 - Constipation: n = 92 - Diarrhea: n = 76 - Vomiting: n = 54 - Dyspepsia: n = 25 - Decreased appetite: n = 60   *GI disorders: n = 310  *Gallbladder-related disorders: n = 7  *Hepatic disorders: n = 11 | The number of participants with GI disorders was numerically higher with cagrilintide than pooled placebo. The number of GI disorders reported increased with cagrilintide dose; most were non-serious and mild or moderate and similar to that with liraglutide 3·0 mg. |
| Lundgren J R et al. / 2021 / Denmark | randomized, head-to-head, placebo-controlled trial | - **Population**: Non-diabetic participants with obesity - **Total subjects**: 215 enrolled, 195 completed - **BMI**: 37.0±2.9 before low calorie diet, 32.6±2.9 after low calorie diet at randomization - **Mean age (years):** 43±12 - **Sex:** Male 71 (36%), female 124 (64%) - **Intervention:** fter an 8-week low-calorie diet, participants were randomly assigned for 1 year to one off our strategies: a moderate- to-vigorous–intensity exercise program plus placebo (exercise group); treatment with liraglutide (3.0 mg per day) plus usual activity (liraglutide group); exercise program plus liraglutide therapy (combination group); or placebo plus usual activity (placebo group). - **Duration:** randomized, head-to-head, placebo-controlled trial for 1 year - **GI adverse events: 348** - Nausea: n = 81 - Abdominal pain: n = 46 - Decreased appetite: n = 40 - Diarrhea: n = 38 - Constipation: n = 34 - Vomiting: n = 34 - Flatulence or abdominal distention: n = 22 - Dyspepsia: n = 19 | GI adverse events were more frequently reported in the groups that received liraglutide (i.e., in the liraglutide group and combination group) than in the other two groups. |
| Rubino D et al. / 2021/ Italy | randomized, double-blind, 68-week phase 3a withdrawal study | - **Population**: Non-diabetic participants with obesity - **Total subjects**: 902 enrolled, 803 completed - **BMI**: 38.4 - **Mean age (years):** 47 (semaglutide), 46 (placebo) - **Sex:** female 429 (80.2%), male 106 (19.8%) (semaglutide), female 205 (76.5%), male 63 (25.5%) (placebo) - **Intervention:** participants received once-weekly subcutaneous semaglutide during run-in. After 20 weeks (16 weeks of dose escalation; 4 weeks of maintenance dose), 803 participants (89.0%) who reached the 2.4-mg/wk semaglutide maintenance dose were randomized (2:1) to 48 weeks of continued subcutaneous semaglutide (n = 535) or switched to placebo (n = 268), plus lifestyle intervention in both groups. - **Duration:** Randomized, double-blind, 68-week phase 3a withdrawal study - **GI adverse events: 1,061** - Nausea: n = 105 - Diarrhea: n = 114 - Constipation: n = 75 - Vomiting: n = 88 - Abdominal pain: n = 46   *GI disorders: n = 607  *Gallbladder-related disorders: n = 15   - *Hepatic disorders: n = 11 | Typical of this class, transient, mild to moderate GI tract disorders were the most fre- quently reported adverse events. More of these events occurred during the run-in period, when semaglutide was escalated to the target dose, compared with the randomized period, despite the randomized period being twice as long. Over the entire trial, few participants in either group discontinued treatment because of adverse events, with the majority of participants who continued semaglutide and completed treatment receiving the 2.4-mg dose at week 68. These results indicate that most participants tolerated the strict up-titration schedule in the trial, and those who continued treatment at the 2.4-mg/wk dose beyond 20 weeks were unlikely to experience significant tolerability challenges thereafter. |
| Saxena A R et al. / 2021 / USA | randomized, double-blind, placebo-controlled study | - **Population**: Non-diabetic participants with obesity - **Total subjects**: 61 enrolled, 56 completed - **BMI**: 34.18 (liraglutide), 34.77 (placebo) - **Mean age (years):** 43 (liraglutide), 48.1 (placebo) - **Sex:** female 38 (67.9%), 18 (32.1%) - **Intervention:** participants received subcutaneous liraglutide (titrated to 3.0 mg/day) or placebo once daily, with inpatient assessments at baseline and weeks 3 and 6 - **Duration:** Randomized, double-blind, placebo-controlled study for 6 weeks - **GI adverse events: 73** - Nausea: n = 15 - Abdominal distension: n = 4 - Upper abdominal pain: n = 6 - Constipation: n = 9 - Diarrhea: n = 11 - Dyspepsia: n = 7 - Nausea: n = 15 - Vomiting: n = 6 | The most frequently reported treatment‐emergent adverse events were nausea, diarrhea, headache, constipation. Two participants discontinued due to vomiting (while receiving 1.2 mg liraglutide), one due to abdominal pain (while receiving 1.2 mg liraglutide), and one due to nausea, headache, and anxiety (while receiving 0.6 mg liraglutide). All adverse events were mild or moderate in intensity and resolved by the last follow‐up. |
| Wadden T A et al. / 2021 / USA | randomized, double-blind, parallel-group, phase 3a study | - **Population**: Non-diabetic participants with overweight/obesity and at least one dysmetabolic comorbidity - **Total subjects**: 611 enrolled, 567 completed - **BMI**: 38.1 (semaglutide 2.4 mg), 37.8 (placebo) - **Mean age (years):** 46 - **Sex:** female 215 (77.4%), male 92 (22.6%) (semaglutide 2.4 mg), female 180 (88.2%), male 24 (11.8%) (placebo) - **Intervention:** participants were randomized (2:1) to semaglutide, 2.4 mg or placebo, both combined with a low-calorie diet for the first 8 weeks and intensive behavioral therapy - **Duration:** randomized, double-blind, parallel-group, 68-week, phase 3a study - **GI adverse events: 1,207** - Nausea: n = 237 - Constipation: n = 150 - Diarrhea: n = 147 - Vomiting: n = 111 - Abdominal pain: n = 54 - Flatulence: n = 47 - Abdominal distention: 41 - Gastroenteritis viral: 42   *Gastrointestinal disorders: n = 337  * Gallbladder-related disorders: n = 20  * Cholelithiasis: n = 13   - * Hepatic disorders: n = 8 | GI disorders were the most frequent and occurred in more participants receiving semaglutide (82.8%) than placebo (63.2%) Most GI events were mild to moderate and of relatively short. The proportion of participants experiencing nausea with semaglutide peaked at approximately 25% at week 20 and declined thereafter, remaining at approximately 15% for the duration of the study. |
| Wharton S et al / 2021 / Canada | randomized, placebo- controlled trial | - **Population**: Non-diabetic participants with obesity or overweight plus at least one weight-related coexisting condition - **Total subjects**: 3379 enrolled - **BMI**: 37.8±6.7 (semaglutide 2.4 mg OW), 38±6.5 (placebo) - **Mean age (years):** 46±13 (semaglutide 2.4 mg OW), 47±12 (placebo) - **Sex:** female 1453 (74%), male 488 (26%) - **Intervention:** each STEP trial included an initial dose escalation for semaglutide, with initiation at 0.25 mg once weekly for 4 weeks, increased at 4-weekly intervals to 0.5 mg, 1.0 mg, 1.7 mg and finally 2.4 mg at week 16. - **Duration:** randomized, placebo- controlled trial for 75 weeks - **GI adverse events: 4,157** - Nausea: n = 1,025 - Diarrhea: n = 722 - Vomiting: n = 578 - Constipation: n = 588 - Dyspepsia: n = 205 - Abdominal pain: n = 232 - Abdominal pain upper: n = 194 - Abdominal distension: n = 169 - Eructation: n = 178 - Flatulence: n = 142 - Gastroesophageal reflux disease: n = 124 | Pooled data from the STEP 1-3 trials demonstrate frequent reports of GI adverse effects in both semaglutide 2.4 mg and placebo arms, with nausea, diarrhoea, vomiting and constipation the most common side effects with semaglutide. The relatively high incidence of GI adverse effects in the placebo arms was not unexpected and may in part reflect the known underlying increased risk of GI disorders associated with obesity. |
| Garvey T W et al. /  2022 / USA | Randomized, double-blind, placebo-controlled,  multinational trial | - **Population**: Non-diabetic participants with obesity or overweight and at least one weight-related comorbidity - **Total subjects**: 304 enrolled, 282 completed - **BMI**: 38.6 (semaglutide), 38.5 (placebo) - **Mean age (years):** 47.3 (semaglutide), 47.4 (placebo) - **Sex:** Female: 236 (78%), Male: 68 (22%) - **Intervention:** Semaglutide vs. placebo. There was no lifestyle intervention. - **Duration:** A randomized, double-blind, placebo-controlled, multinational trial for 104 weeks - **GI adverse events: n = 1,354** - Gastrointestinal disorders leading to trial product discontinuation: n = 7 - Nausea: n = 213 - Diarrhea: n = 108 - Constipation: n = 62 - Vomiting: n = 78 - Abdominal pain upper: n = 23 - Abdominal pain: n = 32 - Dyspepsia: n = 24 - Flatulence: n = 25 - Gastroenteritis: n = 28 - Decreased appetite: n = 18 - Eructation: n = 21   *GI disorders: n = 696  *Gallbladder-related disorders: n = 6  *Hepatobiliary disorders: n = 6  *Cholelithiasis: n = 3  *Hepatic disorders: n = 4 | GI disorders were the most common adverse events with semaglutide, typically transient, of mild-to-moderate severity, occurring during dose escalation, and infrequently leading to treatment discontinuation. |
| Rubino D et al. / 2022/ Italy | randomized, open-label, 68-week, phase 3b trial | - **Population**: Non-diabetic participants with overweight/obesity - **Total subjects**: 387 enrolled, 338 completed - **BMI**: 37 (semaglutide), 37.2 (liraglutide), 38.8 (placebo) - **Mean age (years):** 48 (semaglutide), 49 (liraglutide), 51 (placebo) - **Sex:** female 102 (81%), male 24 (19%) (semaglutide), female 97 (76.4%), male 30 (23.6%) (liraglutide), female 66 (77.6%), male 19 (22.4%) (placebo) - **Intervention:** Participants were randomized (3:1:3:1) to receive once-weekly subcutaneous semaglutide, 2.4 mg (16-week escalation; n = 126), or matching placebo, or once-daily subcutaneous liraglutide, 3.0 mg (4-week escalation; n = 127), or matching placebo, plus diet and physical activity. Participants unable to tolerate 2.4 mg of semaglutide could receive 1.7 mg; participants unable to tolerate 3.0 mg of liraglutide discontinued treatment and could restart the 4-week titration. Placebo groups were pooled (n = 85). - **Duration:** randomized, open-label, 68-week, phase 3b trial - **GI adverse events: 1,304** - Nausea: n = 232 - Diarrhea: n = 88 - Constipation: n = 132 - Eructation: n = 25 - Decreased appetite: n = 33 - Dyspepsia: n = 31   *GI disorders: n = 753  *Gallbladder-related disorders: n = 7  *Hepatic disorders: n = 3 | Most GI events were mild to moderate in severity (severe GI events were reported by 3.2% [n = 4], 2.4% [n = 3], and 3.5% [n = 3] with semaglutide, liraglutide, and placebo, respectively), transient, and resolved without permanent treatment discontinuation. |
| Knop F K et al. /  2023 / Denmark | Randomized, double-blind, placebo-controlled, phase 3, superiority trial | - **Population**: Non-diabetic participants with obesity or overweight and at least one weight-related comorbidity - **Total subjects**: 667 enrolled, 627 completed - **BMI**: 37.5 - **Mean age (years):** 50 - **Sex:** Female: 485 (73%), male: 182 (27%) - **Intervention:** Semaglutide vs. placebo. There was no lifestyle intervention. - **Duration:** A randomized, double-blind, placebo-controlled, phase 3, superiority trial for 68 weeks - **GI adverse events: n = 1,080** - Gastrointestinal disorders leading to trial product discontinuation: n = 19 - Nausea: n = 331 - Diarrhea: n = 169 - Constipation: n = 123 - Vomiting: n = 154 - Abdominal pain upper: n = 45 - Abdominal pain: n = 28 - Dyspepsia: n = 64 - Flatulence: n = 22 - Gastroenteritis: n = 22 - Decreased appetite: n = 61 - Eructation: n = 42   *GI disorders: n = 1136  *Gallbladder-related disorders: n = 15  *Hepatobiliary disorders: n = 15  *Cholelithiasis: n = 12  *Hepatic disorders: n = 7 | GI-related adverse events were more common in Semaglutide group than with placebo and were associated with the dose escalation period. |
| Wadden T A et al. / 2023 / USA | randomized, double-blind, placebo-controlled trial | - **Population**: Non-diabetic participants with overweight/obesity and at least one dysmetabolic comorbidity - **Total subjects**: 579 enrolled, 479 completed - **BMI**: 38.7 (tirzepatide), 38.4 (placebo) - **Mean age (years):** 45.6 - **Sex:** female 364 (62.9%), male 215 (37.1) - **Intervention:** ≥5.0% weight reduction after a 12-week intensive lifestyle intervention, to tirzepatide maximum tolerated dose (10 or 15 mg) or placebo once weekly - **Duration:** randomized, double-blind, placebo-controlled trial for 72 weeks - **GI adverse events: 515** - Nausea: n = 138 - Vomiting: n = 58 - Diarrhea: n = 92 - Dyspepsia: n = 30 - Constipation: n = 68 - Abdominal pain: n = 30 - Decreased appetite: n = 27 - Flatulence: n = 19 - Gastroesophageal reflux disease: n = 19 - Eructation: n = 16   *Gastrointestinal disorders: n = 16  * Gallbladder-related disorders: n = 2 | Mild-to-moderate GI events were the most frequent treatment-emergent adverse events, mostly transient and occurring during dose escalation. Compared with the tirzepatide 15 mg group in SURMOUNT-1, the tirzepatide group in this study had modestly higher rates of GI adverse events and treatment discontinuation due to adverse events. |
| Wharton S et al / 2023 / USA | phase 2, randomized, double-blind trial | - **Population**: Non-diabetic participants with obesity or overweight plus at least one weight-related coexisting condition - **Total subjects**: 272 enrolled, 235 completed - **BMI**: 37.9 - **Mean age (years):** 54.2 - **Sex:** female 161 (59%), male 111 (41%) - **Intervention:** participants were randomly assigned to receive orforglipron at one of four doses (12, 24, 36, or 45 mg) or placebo once daily for 36 weeks. - **Duration:** phase 2, randomized, double-blind trial for 36 weeks - **GI adverse events: 368** - Nausea: n = 106 - Vomiting: n = 59 - Constipation: n = 100 - Diarrhea: n = 51 - Gastroesophageal reflux disease: n = 19 - Dyspepsia: n = 19 - Abdominal pain: n = 13 - *Gastrointestinal polyp hemorrhage: n = 1 | Although GI events occurred at a higher incidence than desired, they allowed us to find the doses that were most efficacious. The pattern of GI events was informative, as was the incidence (for nausea, 37 to 58% with orforglipron and 10% with placebo; for vomiting, 14 to 32% with orforglipron and 6% with placebo). These findings suggest that lower starting doses and slower dose escalation are indicated for reducing GI events and reaching the target dose; this is the concept used for injectable GLP-1 receptor agonists. |
| Zhang Y et al. / 2023 / China | randomized, controlled, open-label clinical trial | - **Population**: Non-diabetic females with overweight/obesity and PCOS - **Total subjects**: 68 enrolled - **BMI**: 29.68 (dulaglutide Combined with CRD therapy), 29.71 (CRD therapy) - **Mean age (years):** 30.31 (dulaglutide Combined with CRD therapy), 28.64 (CRD therapy) - **Sex:** female 68 (100%) - **Intervention:** participants were randomized to receive dulaglutide combined with CRD or CRD alone, hinged on the predetermined number generated by a computer with a 1:1 allocation, which was concealed using opaque, sealed, and serially numbered envelopes. Participants remained on their prescribed treatment program until they reached the 7% weight loss goal (determined by initial body weight at randomization), which was reported to be associated with ameliorative metabolic outcomes. The groups both received dietary counseling and were urged to engage in more physical activities throughout the program (30 min of moderate-intensity aerobic exercise per day, 5–7 days a week, and 10–20 min of resistance exercise, 3 times a week). Participants in the dulaglutide + CRD group received dulaglutide treatment weekly at a dose of 1.5 mg by subcutaneous injection.   **Duration:** randomized, controlled, open-label clinical trial for 6 months   - **GI adverse events: 27** - Nausea: n = 8 - Vomiting: n = 7 - Constipation: n = 4 - Loss of appetite: n = 4 - Abdominal distension: n = 2 - Abdominal pain: n = 1 - Eructation: n = 1 | Two participants developed GI reactions within the first week of starting dulaglutide injection; therefore, they were unwilling to continue to dulaglutide treatment and withdrew from the trial. Approximately 37.14% of the participants with dulaglutide treatment reported at least one GI treatment-emergent adverse event. Most of GI effects were mild to moderate in severity, and most were more pronounced within the first 2 weeks, and then gradually ameliorated within 1 month. |
| BMI: body mass index; PBO: placebo; DAPA: dapagliflozin; ExQW: subcutaneous exenatide; EXE: exenatide; BID: two times a day; QD: once a day; CRD: calorie-restricted diet. | | | |
