# Supplementary material for: Gastrointestinal adverse events associated with GLP-1 RA in non-diabetic patients with overweight or obesity: a systematic review and network meta-analysis
Source: Int J Obes (Lond). 2025 Aug 13;49(10):1946–57. doi: 10.1038/s41366-025-01859-6 (PMC12532569; doi:10.1038/s41366-025-01859-6)
Supplement: Supplementary file 4 — Network meta-analysis summary for the assessed gastrointestinal adverse events associated with GLP-1 receptor agonists in subjects with overweight and obesity [file 41366_2025_1859_MOESM4_ESM.docx]

| **Supplementary table 2.** Network meta-analysis summary for the assessed gastrointestinal adverse events associated with GLP-1 receptor agonists in subjects with overweight and obesity | | | | | | | | | | | | |
| --- | --- | --- | --- | --- | --- | --- | --- | --- | --- | --- | --- | --- |
| Nausea (*n* = 29 studies) | | | Heterogeneity: I^2^ = 17.8% (0% - 49.2%), *p* = 0.209 | | | | | | | | | |
| Drug | RR | | 95% CI | | *z* | | *p*-value | | Rank | | P score | |
| Metformin | 1.4436 | | 0.6185; 3.3694 | | 0.85 | | 0.3959 | | 1 | | 0.8328 | |
| Cagrilinitide | 2.2983 | | 1.6884, 3.1285 | | 5.29 | | <0.0001 | | 2 | | 0.6418 | |
| Exenatide | 2.6645 | | 1.3956, 5.0870 | | 2.97 | | 0.0030 | | 3 | | 0.4433 | |
| Tirzepatide | 2.8997 | | 2.0048, 4.1939 | | 5.65 | | < 0.0001 | | 4 | | 0.3805 | |
| Semaglutide | 2.9464 | | 2.6138, 3.3213 | | 17.68 | | < 0.0001 | | 5 | | 0.3672 | |
| Liraglutide | 3.0919 | | 2.7271, 3.5056 | | 17.62 | | < 0.0001 | | 6 | | 0.2656 | |
| Orforglipron | 4.7748 | | 2.0161, 11.3085 | | 3.55 | | 0.0004 | | 7 | | 0.0973 | |
| Vomiting (*n* = 23) | | | Heterogeneity: I^2^ = 0% (0% - 48%), *p* = 0.555 | | | | | | | | | |
| Drug | RR | | 95% CI | | *z* | | *p*-value | | Rank | | P score | |
| Cagrilinitide | 1.3701 | | 0.8106; 2.3158 | | 1.18 | | 0.2397 | | 1 | | 0.7937 | |
| Metformin | 2.8865 | | 0.1367; 60.9718 | | 0.68 | | 0.4958 | | 2 | | 0.5703 | |
| Liraglutide | 3.8722 | | 3.1510; 4.7585 | | 12.87 | | < 0.0001 | | 3 | | 0.4730 | |
| Orforglipron | 4.4294 | | 1.4471; 13.5578 | | 2.61 | | 0.0091 | | 4 | | 0.3908 | |
| Exenatide | 4.5238 | | 0.2472; 82.7745 | | 1.02 | | 0.3088 | | 5 | | 0.3894 | |
| Semaglutide | 4.2108 | | 3.5822; 4.9497 | | 17.43 | | < 0.0001 | | 6 | | 0.3833 | |
| Tirzepatide | 13.2265 | | 4.8471; 36.0917 | | 5.04 | | < 0.0001 | | 7 | | 0.0748 | |
| Diarrhea (*n* = 24) | | | Heterogeneity: I^2^ = 44.3% (6.8% - 66.7%), *p* = 0.016 | | | | | | | | | |
| Drug | RR | | 95% CI | | *z* | | *p*-value | | Rank | | P score | |
| Exenatide | 0.1756 | | 0.0231; 1.3355 | | -1.68 | | 0.0929 | | 1 | | 0.9774 | |
| Metformin | 0.7277 | | 0.1254; 4.2241 | | -0.35 | | 0.7232 | | 2 | | 0.697 | |
| Cagrilinitide | 1.1956 | | 0.6819; 2.0961 | | 0.62 | | 0.5329 | | 3 | | 0.6161 | |
| Semaglutide | 1.7714 | | 1.4679; 2.1377 | | 5.96 | | < 0.0001 | | 4 | | 0.3629 | |
| Liraglutide | 1.8231 | | 1.4804; 2.2452 | | 5.65 | | < 0.0001 | | 5 | | 0.3284 | |
| Orforglipron | 2.2973 | | 0.8893; 5.9347 | | 1.72 | | 0.0859 | | 6 | | 0.2396 | |
| Tirzepatide | 3.3537 | | 1.9216; 5.8533 | | 4.26 | | < 0.0001 | | 7 | | 0.0500 | |
| Constipation (*n* = 24) | | | Heterogeneity: I^2^ = 51.4% (19.9% - 70.5%), *p* = 0.004 | | | | | | | | | |
| Drug | RR | | 95% CI | | *z* | | *p*-value | | Rank | | P score | |
| Cagrilinitide | 1.2782 | | 0.6950; 2.3508 | | 0.79 | | 0.4299 | | 1 | | 0.8301 | |
| Semaglutide | 2.0979 | | 1.6722; 2.6321 | | 6.40 | | < 0.0001 | | 2 | | 0.5873 | |
| Liraglutide | 2.2368 | | 1.7424; 2.8715 | | 6.32 | | < 0.0001 | | 3 | | 0.5237 | |
| Exenatide | 5.0000 | | 0.2379; 105.0709 | | 1.04 | | 0.3003 | | 4 | | 0.3936 | |
| Tirzepatide | 3.3575 | | 1.7007; 6.6282 | | 3.49 | | 0.0005 | | 5 | | 0.3325 | |
| Orforglipron | 4.0541 | | 1.1930; 13.7766 | | 2.24 | | 0.0249 | | 6 | | 0.3100 | |
| Metformin | 26.3652 | | 0.7815; 889.4372 | | 1.82 | | 0.0684 | | 7 | | 0.0817 | |
| GERD (*n* = 8) | | | Heterogeneity: I^2^ = 50% (0% - 81.7%), *p* = 0.092 | | | | | | | | | |
| Drug | RR | | 95% CI | | *z* | | *p*-value | | Rank | | P score | |
| Liraglutide | 1.8614 | | 0.6671; 5.1939 | | 1.19 | | 0.2354 | | 1 | | 0.5597 | |
| Semaglutide | 2.4321 | | 1.1013; 5.3710 | | 2.20 | | 0.0279 | | 2 | | 0.4114 | |
| Tirzepatide | 2.7616 | | 0.7517; 10.1459 | | 1.53 | | 0.1260 | | 3 | | 0.3727 | |
| Orforglipron | 4.9550 | | 0.5427; 45.2367 | | 1.42 | | 0.1561 | | 4 | | 0.2244 | |
| Eructation (*n* = 11) | | | Heterogeneity: I^2^ = 47.3% (0% - 75.5%), *p* = 0.05 | | | | | | | | | |
| Drug | RR | | 95% CI | | *z* | | *p*-value | | Rank | | P score | |
| Liraglutide | 3.2373 | | 1.2419; 8.4388 | | 2.40 | | 0.0163 | | 1 | | 0.5993 | |
| Exenatide | 3.0000 | | 0.1005; 89.5452 | | 0.63 | | 0.5261 | | 2 | | 0.5745 | |
| Tirzepatide | 5.4262 | | 0.9687; 30.3963 | | 1.92 | | 0.0544 | | 3 | | 0.4186 | |
| Semaglutide | 7.8838 | | 3.7606; 16.5278 | | 5.47 | | < 0.0001 | | 4 | | 0.2689 | |
| Orforglipron | 16.1146 | | 0.7796; 333.0789 | | 1.80 | | 0.0720 | | 5 | | 0.2057 | |
| Abdominal distention (*n* = 11) | | | Heterogeneity: I^2^ = 0% (0% - 67.6%), *p* = 0.75 | | | | | | | | | |
| Drug | RR | | 95% CI | | *z* | | *p*-value | | Rank | | P score | |
| Semaglutide | 1.4245 | | 1.1320; 1.7925 | | 3.02 | | 0.0026 | | 1 | | 0.6085 | |
| Liraglutide | 1.5656 | | 0.8445; 2.9025 | | 1.42 | | 0.1547 | | 2 | | 0.5614 | |
| Metformin | 6.5609 | | 0.6262; 68.7366 | | 1.57 | | 0.1165 | | 3 | | 0.1961 | |
| Exenatide | 6.5609 | | 0.8253; 52.1544 | | 1.78 | | 0.0753 | | 4 | | 0.1776 | |
| Abdominal pain upper (*n* = 13 studies) | | | | Heterogeneity: I^2^ = 0% (0% - 62.4%), *p* = 0.673 | | | | | | | | |
| Drug | | RR | | 95% CI | | *z* | | *p*-value | | Rank | | P score |
| Exenatide | | 0.3333 | | 0.0140; 7.9482 | | -0.68 | | 0.4972 | | 1 | | 0.8590 |
| Liraglutide | | 1.6919 | | 1.2339; 2.3199 | | 3.26 | | 0.0011 | | 2 | | 0.3852 |
| Metformin | | 1.7589 | | 0.0472; 65.5057 | | 0.31 | | 0.7596 | | 3 | | 0.3611 |
| Semaglutide | | 2.1435 | | 1.6582; 2.7709 | | 5.82 | | < 0.0001 | | 4 | | 0.1776 |
| Abdominal pain (*n* = 12 studies) | | | | Heterogeneity: I^2^ = 60% (16.7% - 80.8%), *p* = 0.01 | | | | | | | | |
| Drug | | RR | | 95% CI | | *z* | | *p*-value | | Rank | | P score |
| Orforglipron | | 1.4640 | | 0.2619; 8.1832 | | 0.43 | | 0.6642 | | 1 | | 0.6295 |
| Liraglutide | | 2.0774 | | 1.0602; 4.0703 | | 2.13 | | 0.0331 | | 2 | | 0.4586 |
| Semaglutide | | 2.3447 | | 1.4134; 3.8898 | | 3.30 | | 0.0010 | | 3 | | 0.3785 |
| Tirzepatide | | 4.3604 | | 1.2866; 14.7780 | | 2.36 | | 0.0181 | | 4 | | 0.1229 |
| Abdominal discomfort (*n* = 1 studies) | | | |  | | | | | | | | |
| Drug | | RR | | 95% CI | | *z* | | *p*-value | | Rank | | P score |
| Liraglutide | | 0.5474 | | 0.0350; 8.5718 | | -0.43 | | 0.6677 | | 1 | | 0.6662 |
| Flatulence (*n* = 8 studies) | | | | Heterogeneity: I^2^ = 0% (0% - 74.6%), *p* = 0.607 | | | | | | | | |
| Drug | | RR | | 95% CI | | *z* | | *p*-value | | Rank | | P score |
| Liraglutide | | 1.3407 | | 0.6862; 2.6198 | | 0.86 | | 0.3909 | | 1 | | 0.5358 |
| Semaglutide | | 1.4005 | | 1.1100; 1.7671 | | 2.84 | | 0.0045 | | 2 | | 0.4507 |
| Tirzepatide | | 2.4164 | | 1.0751; 5.4309 | | 2.14 | | 0.0327 | | 3 | | 0.0849 |
| Cholelithiasis (*n* = 4 studies) | | | | Heterogeneity: I^2^ = 17.7% (0% - 91.4%), *p* = 0.297 | | | | | | | | |
| Drug | | RR | | 95% CI | | *z* | | *p*-value | | Rank | | P score |
| Tirzepatide | | 1.3566 | | 0.2354; 7.8184 | | 0.34 | | 0.7329 | | 1 | | 0.5249 |
| Liraglutide | | 1.7470 | | 0.5471; 5.5783 | | 0.94 | | 0.3462 | | 2 | | 0.3732 |
| Semaglutide | | 1.9181 | | 0.2354; 7.8184 | | 0.34 | | 0.7329 | | 3 | | 0.3488 |
| Decreased appetite (*n* = 6 studies) | | | | Heterogeneity: I^2^ = 36.5% (0% - 70.8%), *p* = 0.126 | | | | | | | | |
| Drug | | RR | | 95% CI | | *z* | | *p*-value | | Rank | | P score |
| Tirzepatide | | 2.2892 | | 0.9685; 5.4110 | | 1.89 | | 0.0592 | | 1 | | 0.5501 |
| Orforglipron | | 3.1532 | | 0.3940; 25.2360 | | 1.08 | | 0.2792 | | 2 | | 0.3982 |
| Semaglutide | | 3.0190 | | 1.6699; 5.4580 | | 3.66 | | 0.0003 | | 3 | | 0.3680 |
| Cagrilinitide | | 3.0684 | | 1.3826; 6.8099 | | 2.76 | | 0.0058 | | 4 | | 0.3604 |
| Liraglutide | | 3.0404 | | 2.1283; 4.3434 | | 6.11 | | < 0.0001 | | 5 | | 0.3577 |
| Gallstone-related (*n* = 2 studies) | | | | Heterogeneity: I^2^ = 0% (NA% - NA%), *p* = 0.643 | | | | | | | | |
| Drug | | RR | | 95% CI | | *z* | | *p*-value | | Rank | | P score |
| Liraglutide | | 0.3770 | | 0.0862; 1.6494 | | -1.30 | | 0.1952 | | 1 | | 0.9024 |
| Cholecystitis (*n* = 2 studies) | | | |  | | | | | | | | |
| Drug | | RR | | 95% CI | | *z* | | *p*-value | | Rank | | P score |
| Tirzepatide | | 0.3391 | | 0.0139; 8.2904 | | -0.66 | | 0.5073 | | 1 | | 0.8128 |
| Liraglutide | | 4.5063 | | 0.2428; 83.6327 | | 1.01 | | 0.3124 | | 2 | | 0.1385 |
| Acute cholecystitis (*n* = 2 studies) | | | |  | | | | | | | | |
| Drug | | RR | | 95% CI | | *z* | | *p*-value | | Rank | | P score |
| Tirzepatide | | 3.0522 | | 0.1249; 74.6134 | | 0.68 | | 0.4938 | | 1 | | 0.4943 |
| Liraglutide | | 12.5176 | | 0.7418; 211.2448 | | 1.75 | | 0.0796 | | 2 | | 0.1491 |
| Acute pancreatitis (*n* = 1 studies) | | | |  | | | | | | | | |
| Drug | | RR | | 95% CI | | *z* | | *p*-value | | Rank | | P score |
| Liraglutide | | 4.5063 | | 0.2428; 83.6327 | | 1.01 | | 0.3124 | | 1 | | 0.1562 |
| Viral gastroenteritis (*n* = 1 studies) | | | |  | | | | | | | | |
| Drug | | RR | | 95% CI | | *z* | | *p*-value | | Rank | | P score |
| Semaglutide | | 1.6194 | | 0.8897; 2.9474 | | 1.58 | | 0.1147 | | 1 | | 0.0573 |
| Feces hard (*n* = 1 studies) | | | |  | | | | | | | | |
| Drug | | RR | | 95% CI | | *z* | | *p*-value | | Rank | | P score |
| Liraglutide | | 2.5000 | | 0.3056; 20.4528 | | 0.85 | | 0.3929 | | 1 | | 0.1964 |
| Infrequent bowel movements (*n* = 1 studies) | | | |  | | | | | | | | |
| Drug | | RR | | 95% CI | | *z* | | *p*-value | | Rank | | P score |
| Liraglutide | | 2.5207 | | 0.1249; 50.8829 | | 0.60 | | 0.5465 | | 1 | | 0.2733 |
