# Supplementary material for: Gastrointestinal adverse events associated with GLP-1 RA in non-diabetic patients with overweight or obesity: a systematic review and network meta-analysis
Source: Int J Obes (Lond). 2025 Aug 13;49(10):1946–57. doi: 10.1038/s41366-025-01859-6 (PMC12532569; doi:10.1038/s41366-025-01859-6)
Supplement: Supplementary file 5 — Supplementary Table 3 [file 41366_2025_1859_MOESM5_ESM.docx]

**Supplementary Table 3.** Cochrane Risk of Bias Tool for RCTs

| **Domain** | **Elkind-Hirsch et al. 2008** | **Astrup et al. 2009** | **Dushai et al. 2011** | **Sze et al. 2011** | **Astrup et al. 2012** |
| --- | --- | --- | --- | --- | --- |
| *Selection bias – Random sequence generation* | - | - | + | ? | - |
| *Selection bias – Allocation concealment* | - | - | ? | ? | - |
| *Performance bias – Blinding (participants and personnel)* | + | - | ? | ? | - |
| *Detection bias – Blinding (outcome assessment)* | + | - | ? | + | - |
| *Attrition bias – Incomplete outcome data* | - | - | - | - | - |
| *Reporting bias – Selective reporting* | - | - | - | - | - |
| *Other bias – Other sources of bias* | - | - | - | - | - |
| **+**, high risk; **-**, low risk; **?**, unclear risk of bias | | | | | |

| **Domain** | **Jensterle et al. 2013** | **Lean et al.**  **2013** | **Wadden et al. 2013** | **Faurschou et al. 2014** | **Iepsen et al. 2014** |
| --- | --- | --- | --- | --- | --- |
| *Selection bias – Random sequence generation* | - | ? | - | - | ? |
| *Selection bias – Allocation concealment* | ? | ? | - | - | ? |
| *Performance bias – Blinding (participants and personnel)* | + | - | - | - | ? |
| *Detection bias – Blinding (outcome assessment)* | + | - | - | - | ? |
| *Attrition bias – Incomplete outcome data* | - | - | - | - | - |
| *Reporting bias – Selective reporting* | - | - | - | - | - |
| *Other bias – Other sources of bias* | - | - | - | - | - |
| **+**, high risk; **-**, low risk; **?**, unclear risk of bias | | | | | |

| **Domain** | **Van can et al. 2014** | **Jensterle et al. 2015** | **Pi-Sunyer et al. 2015** | **Blackman et al. 2016** | **Jensterle et al. 2016** |
| --- | --- | --- | --- | --- | --- |
| *Selection bias – Random sequence generation* | ? | ? | - | - | ? |
| *Selection bias – Allocation concealment* | ? | ? | - | - | ? |
| *Performance bias – Blinding (participants and personnel)* | - | + | ? | - | + |
| *Detection bias – Blinding (outcome assessment)* | - | + | ? | - | + |
| *Attrition bias – Incomplete outcome data* | - | - | - | - | - |
| *Reporting bias – Selective reporting* | - | - | - | - | ? |
| *Other bias – Other sources of bias* | - | - | - | - | - |
| **+**, high risk; **-**, low risk; **?**, unclear risk of bias | | | | | |

| **Domain** | **Halawi et al.**  **2017** | **Liu et al.**  **2017** | **Lundkvist et al. 2017** | **Nylander et al. 2017** | **Zheng et al. 2017** |
| --- | --- | --- | --- | --- | --- |
| *Selection bias – Random sequence generation* | - | - | ? | - | - |
| *Selection bias – Allocation concealment* | - | - | ? | - | - |
| *Performance bias – Blinding (participants and personnel)* | - | + | ? | - | + |
| *Detection bias – Blinding (outcome assessment)* | - | + | - | - | + |
| *Attrition bias – Incomplete outcome data* | - | - | - | - | - |
| *Reporting bias – Selective reporting* | - | ? | - | - | - |
| *Other bias – Other sources of bias* | - | - | - | - | - |
| **+**, high risk; **-**, low risk; **?**, unclear risk of bias | | | | | |

| **Domain** | **Enebo et al.**  **2021** | **Frossing et al. 2018** | **O’Neil et al. 2018** | **Salamun et al. 2018** | **Ma et al. 2020** |
| --- | --- | --- | --- | --- | --- |
| *Selection bias – Random sequence generation* | - | ? | - | ? | - |
| *Selection bias – Allocation concealment* | - | ? | - | ? | - |
| *Performance bias – Blinding (participants and personnel)* | - | ? | - | + | + |
| *Detection bias – Blinding (outcome assessment)* | - | ? | - | + | + |
| *Attrition bias – Incomplete outcome data* | - | - | - | - | - |
| *Reporting bias – Selective reporting* | - | - | - | - | - |
| *Other bias – Other sources of bias* | - | - | - | - | - |
| **+**, high risk; **-**, low risk; **?**, unclear risk of bias | | | | | |

| **Domain** | **Alba et al.**  **2021** | **Gudbergsen et al. 2021** | **Lau et al.**  **2021** | **Lundgren et al. 2021** | **Rubino et al. 2021** |
| --- | --- | --- | --- | --- | --- |
| *Selection bias – Random sequence generation* | ? | - | - | - | - |
| *Selection bias – Allocation concealment* | ? | - | - | - | - |
| *Performance bias – Blinding (participants and personnel)* | - | - | - | ? | ? |
| *Detection bias – Blinding (outcome assessment)* | - | - | - | ? | ? |
| *Attrition bias – Incomplete outcome data* | - | - | - | - | - |
| *Reporting bias – Selective reporting* | - | - | - | ? | - |
| *Other bias – Other sources of bias* | - | - | - | - | - |
| **+**, high risk; **-**, low risk; **?**, unclear risk of bias | | | | | |

| **Domain** | **Saxena et al.**  **2021** | **Wadden et al. 2021** | **Wharton et al. 2021** | **Garvey et al. 2022** | **Rubino et al. 2021** |
| --- | --- | --- | --- | --- | --- |
| *Selection bias – Random sequence generation* | ? | - | ? | - | - |
| *Selection bias – Allocation concealment* | ? | - | ? | - | - |
| *Performance bias – Blinding (participants and personnel)* | - | ? | ? | - | ? |
| *Detection bias – Blinding (outcome assessment)* | + | ? | ? | - | ? |
| *Attrition bias – Incomplete outcome data* | - | - | - | - | - |
| *Reporting bias – Selective reporting* | - | - | - | - | - |
| *Other bias – Other sources of bias* | - | - | - | - | - |
| **+**, high risk; **-**, low risk; **?**, unclear risk of bias | | | | | |

| **Domain** | **Knop et al.**  **2023** | **Wadden et al. 2023** | **Wharton et al. 2023** | **Zhang et al. 2023** |
| --- | --- | --- | --- | --- |
| *Selection bias – Random sequence generation* | - | - | ? | - |
| *Selection bias – Allocation concealment* | - | - | ? | - |
| *Performance bias – Blinding (participants and personnel)* | ? | - | ? | + |
| *Detection bias – Blinding (outcome assessment)* | ? | - | ? | + |
| *Attrition bias – Incomplete outcome data* | - | - | - | - |
| *Reporting bias – Selective reporting* | - | - | - | - |
| *Other bias – Other sources of bias* | - | - | - | - |
| **+**, high risk; **-**, low risk; **?**, unclear risk of bias | | | | |
