# Supplementary material for: Gastrointestinal adverse events associated with GLP-1 RA in non-diabetic patients with overweight or obesity: a systematic review and network meta-analysis
Source: Int J Obes (Lond). 2025 Aug 13;49(10):1946–57. doi: 10.1038/s41366-025-01859-6 (PMC12532569; doi:10.1038/s41366-025-01859-6)
Supplement: Supplementary file 6 — Supplementary figures [file 41366_2025_1859_MOESM6_ESM.docx]

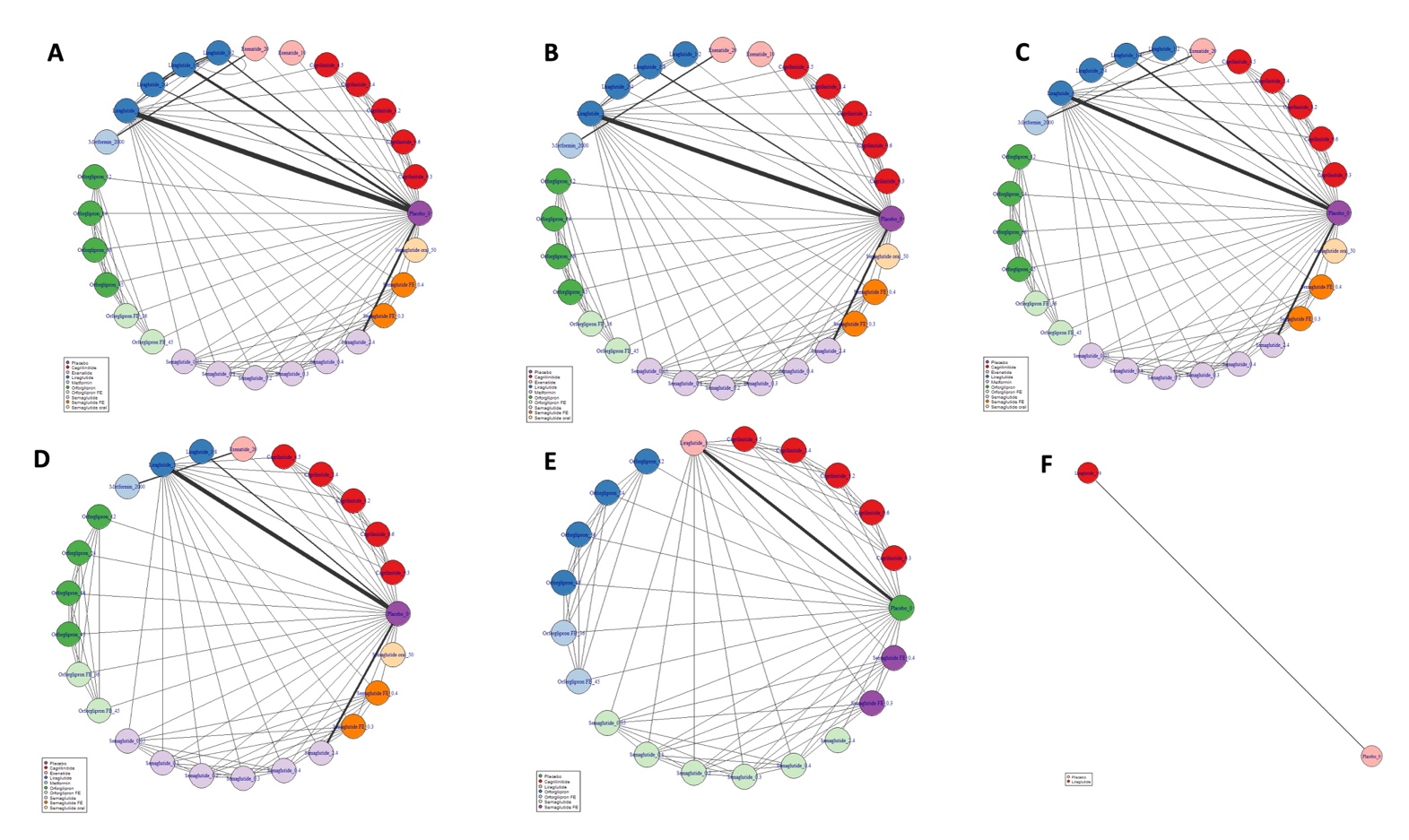


**Supplementary figure 1.** Network dose-response meta-analysis for (A) nausea, (B) vomiting, (C) diarrhea, (D) constipation, (E) decreased appetite, and (F) gallstone-related.

**
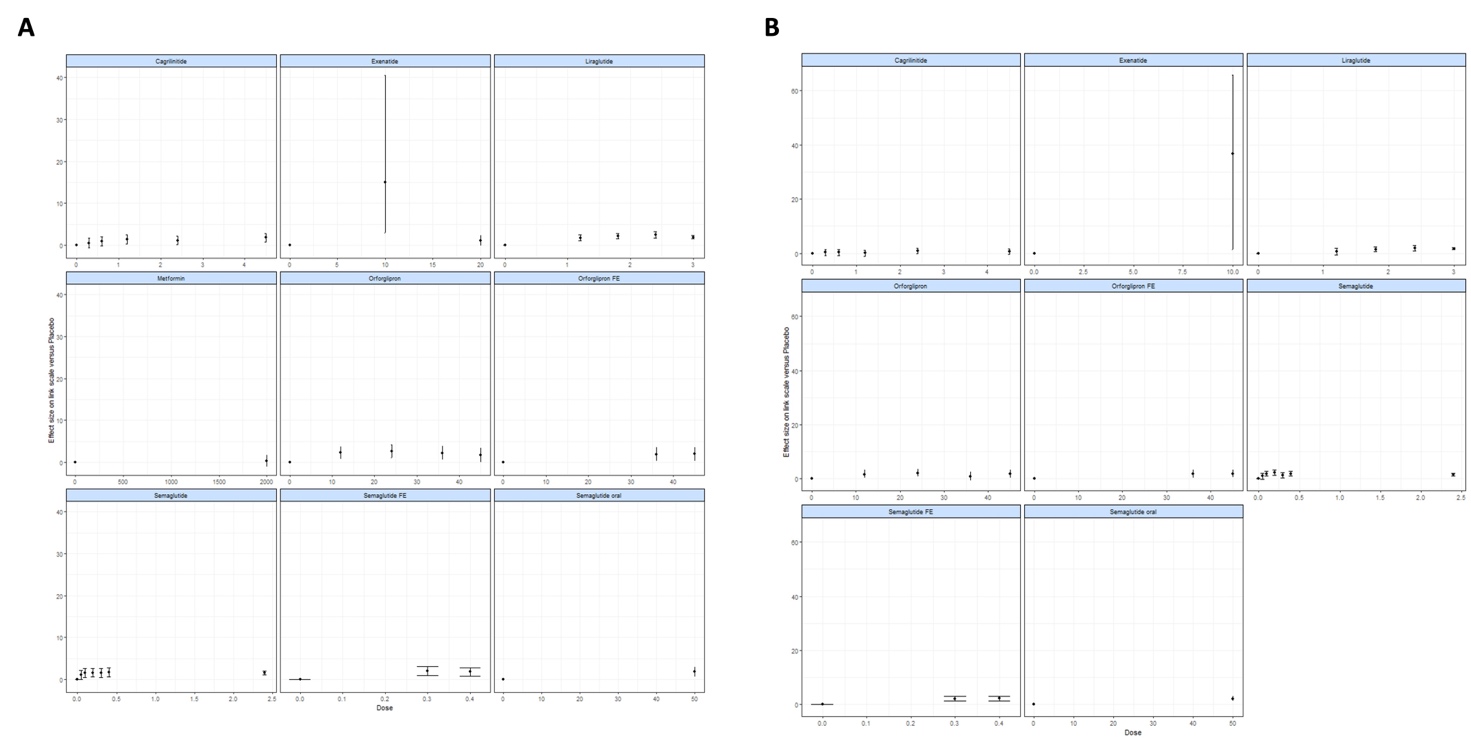
**

**
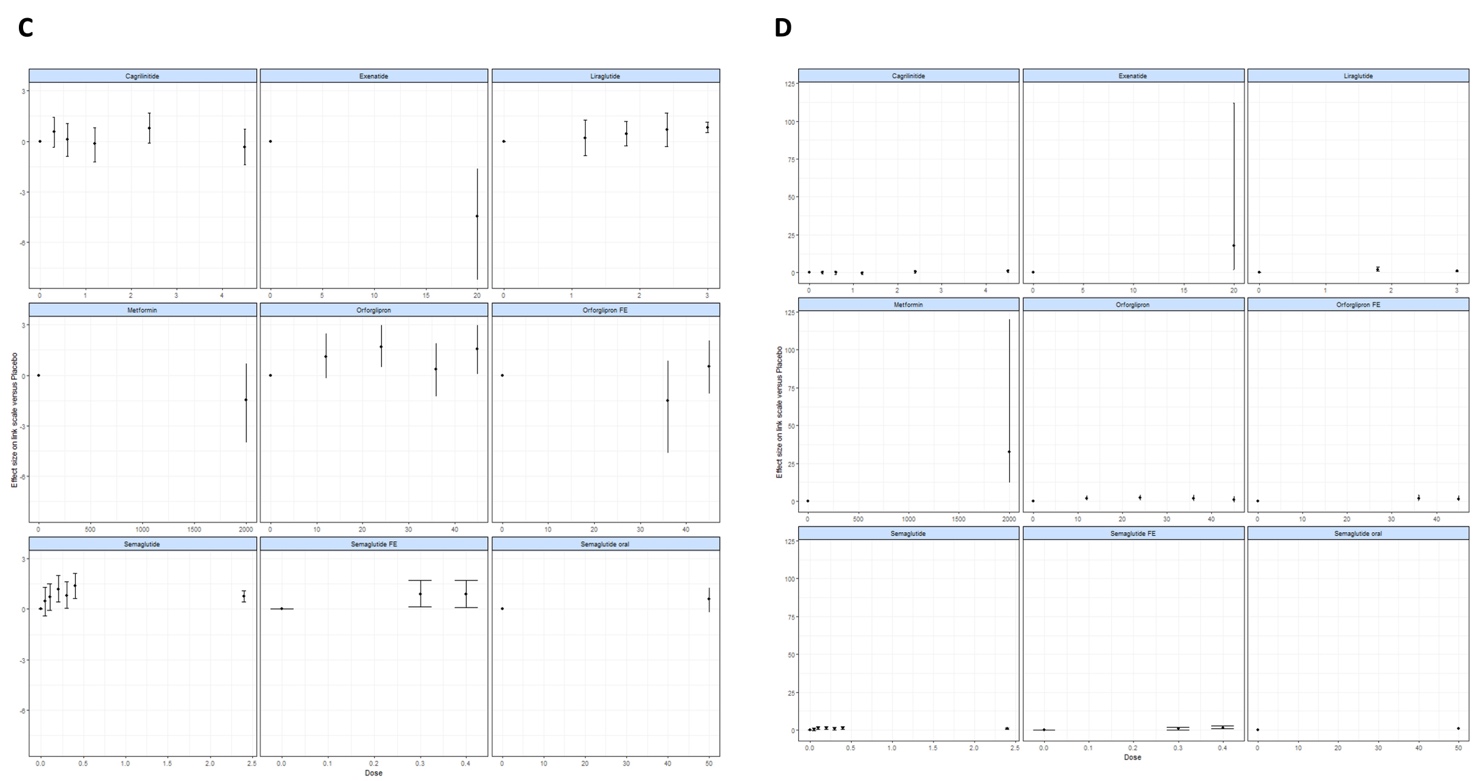
**

**
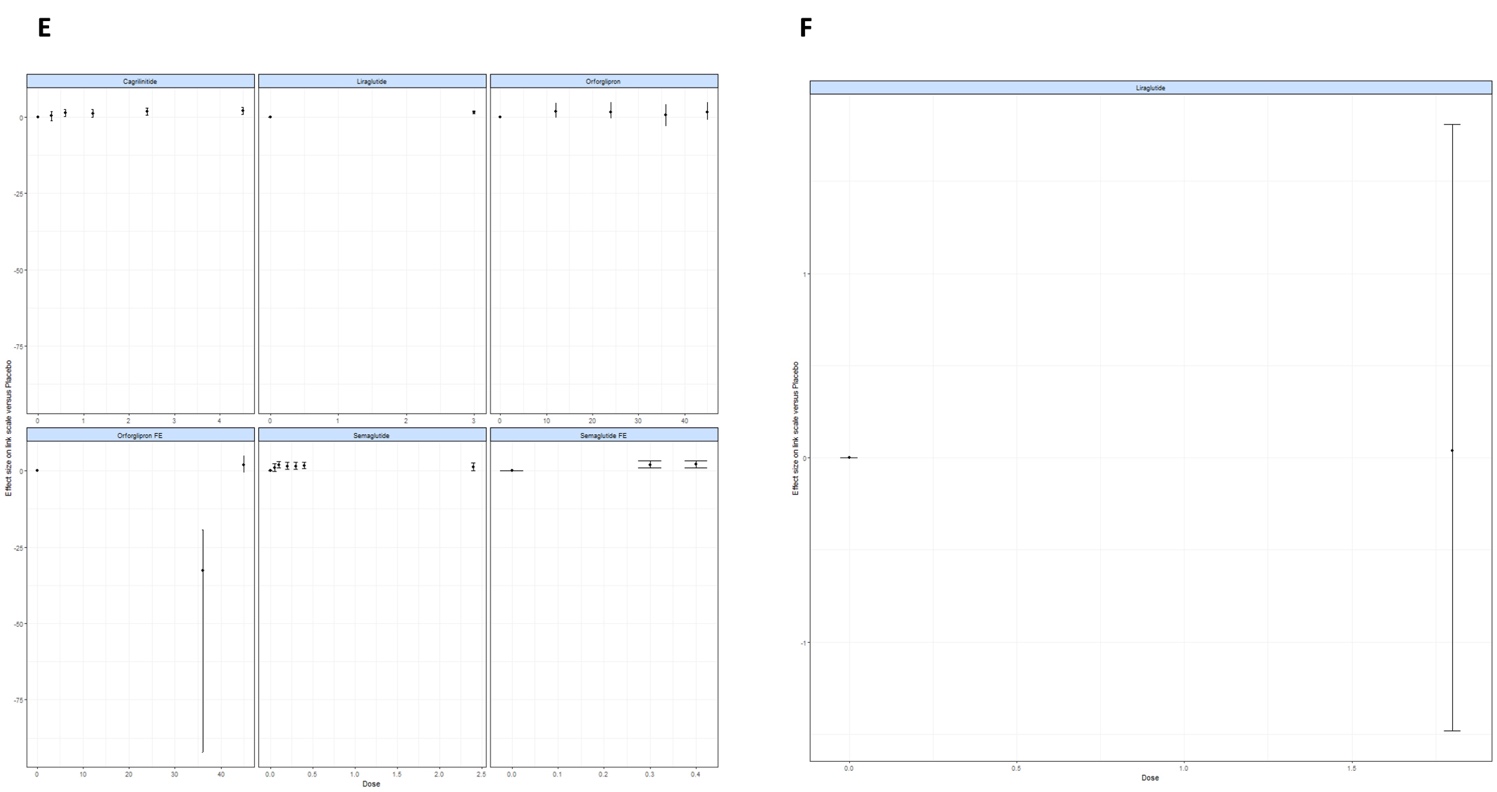
**

**Supplementary figure 2.** Network dose-response meta-analysis estimates by dose for (A) nausea, (B) vomiting, (C) diarrhea, (D) constipation, (E) decreased appetite, and (F) gallstone-related.

**
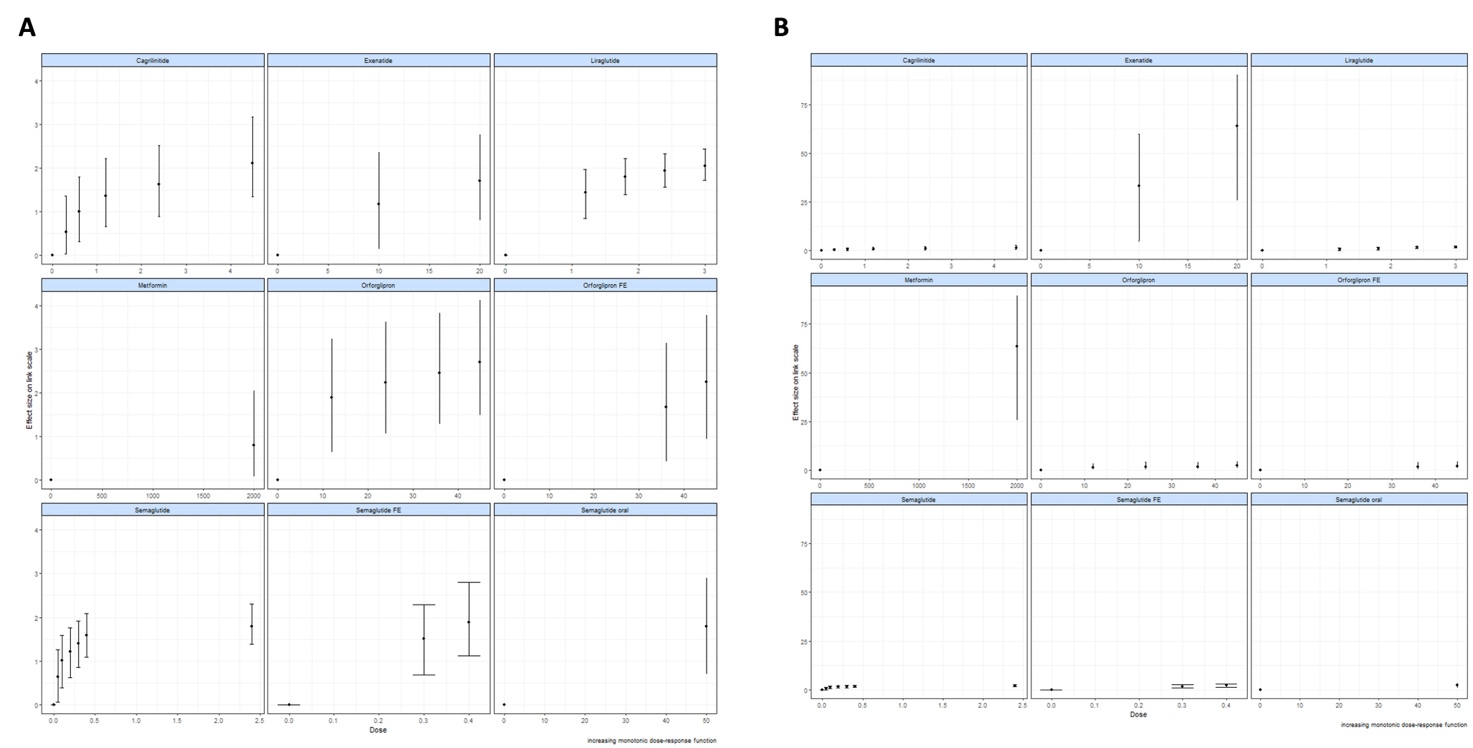
**

**
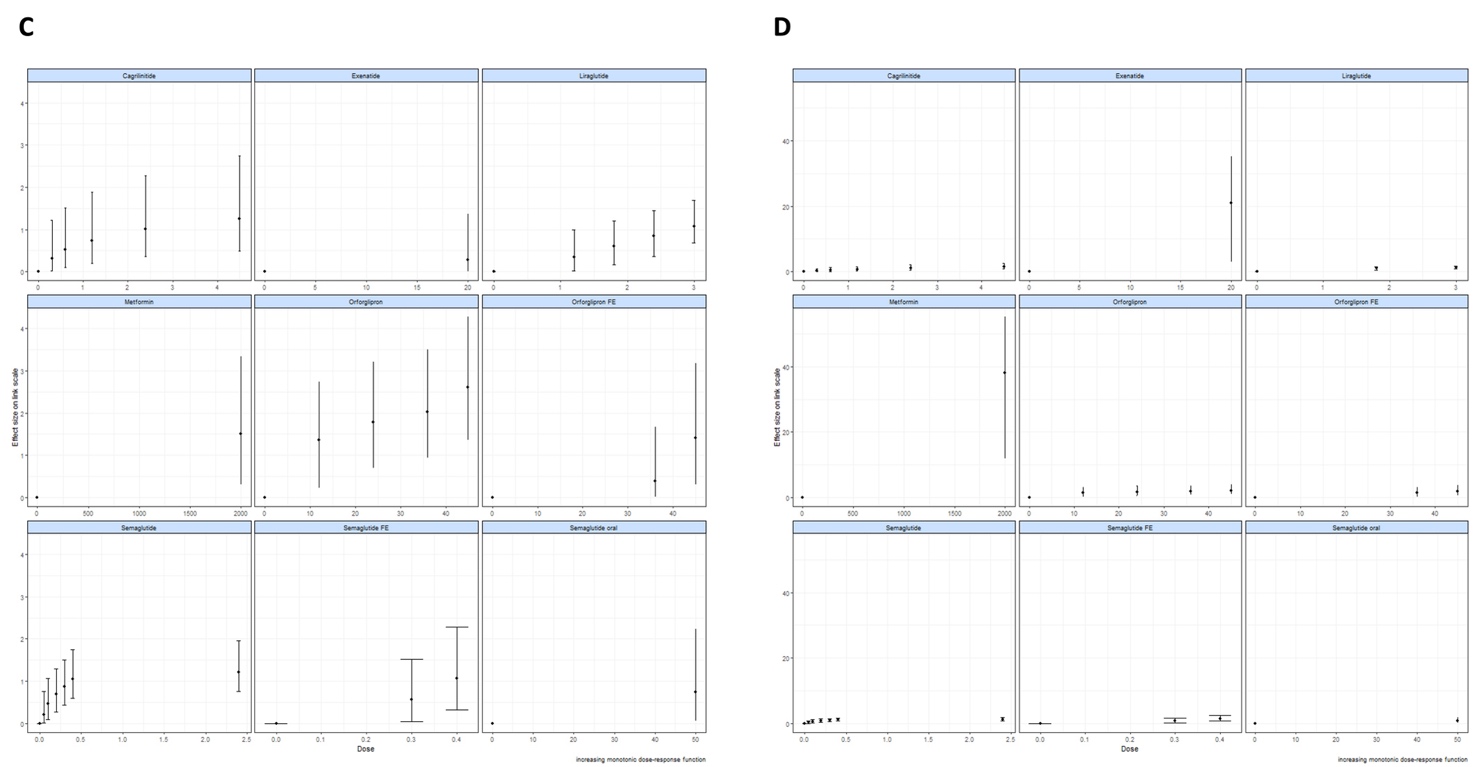
**

**
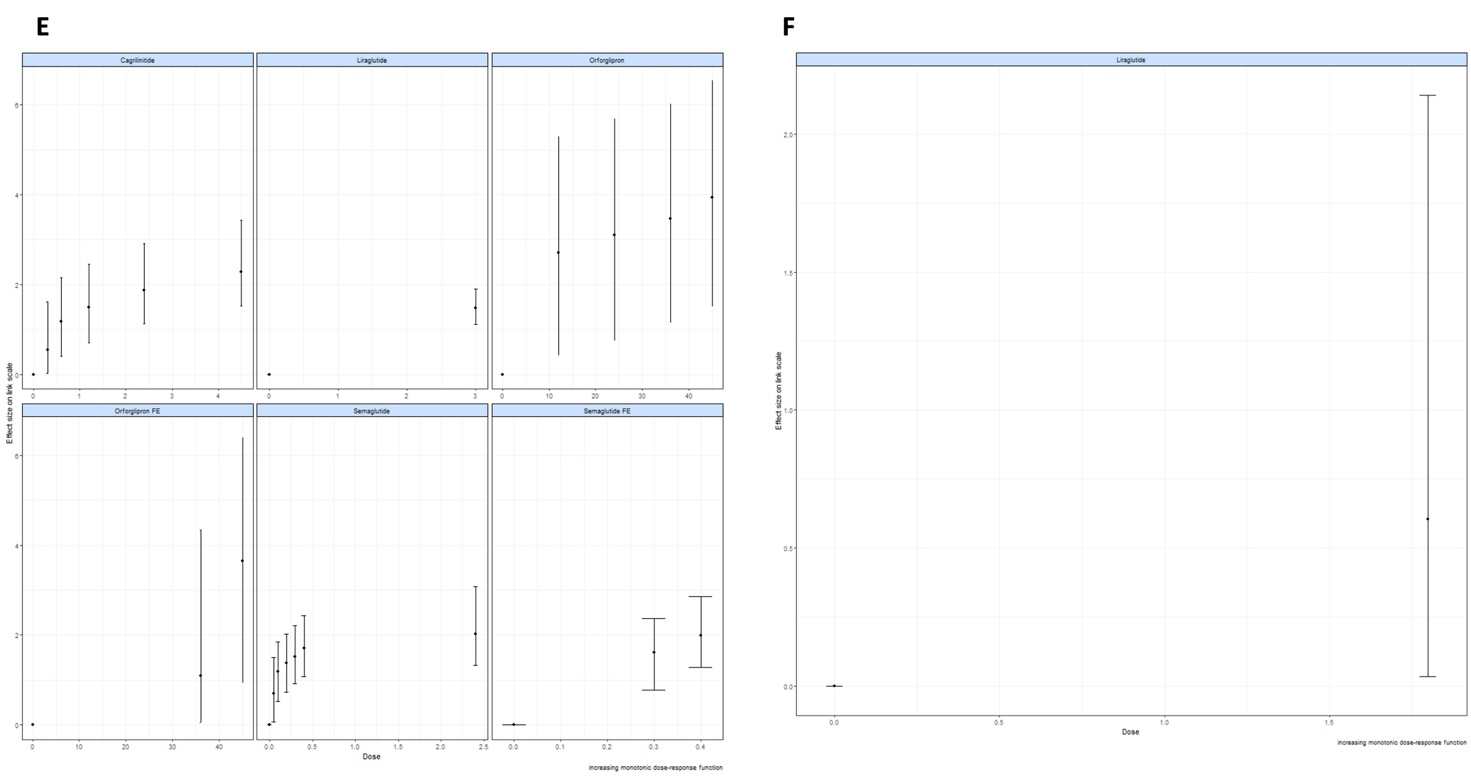
**

**Supplementary figure 3.** Nonparametric monotonic increasing model for (A) nausea, (B) vomiting, (C) diarrhea, (D) constipation, (E) decreased appetite, and (F) gallstone-related.

**
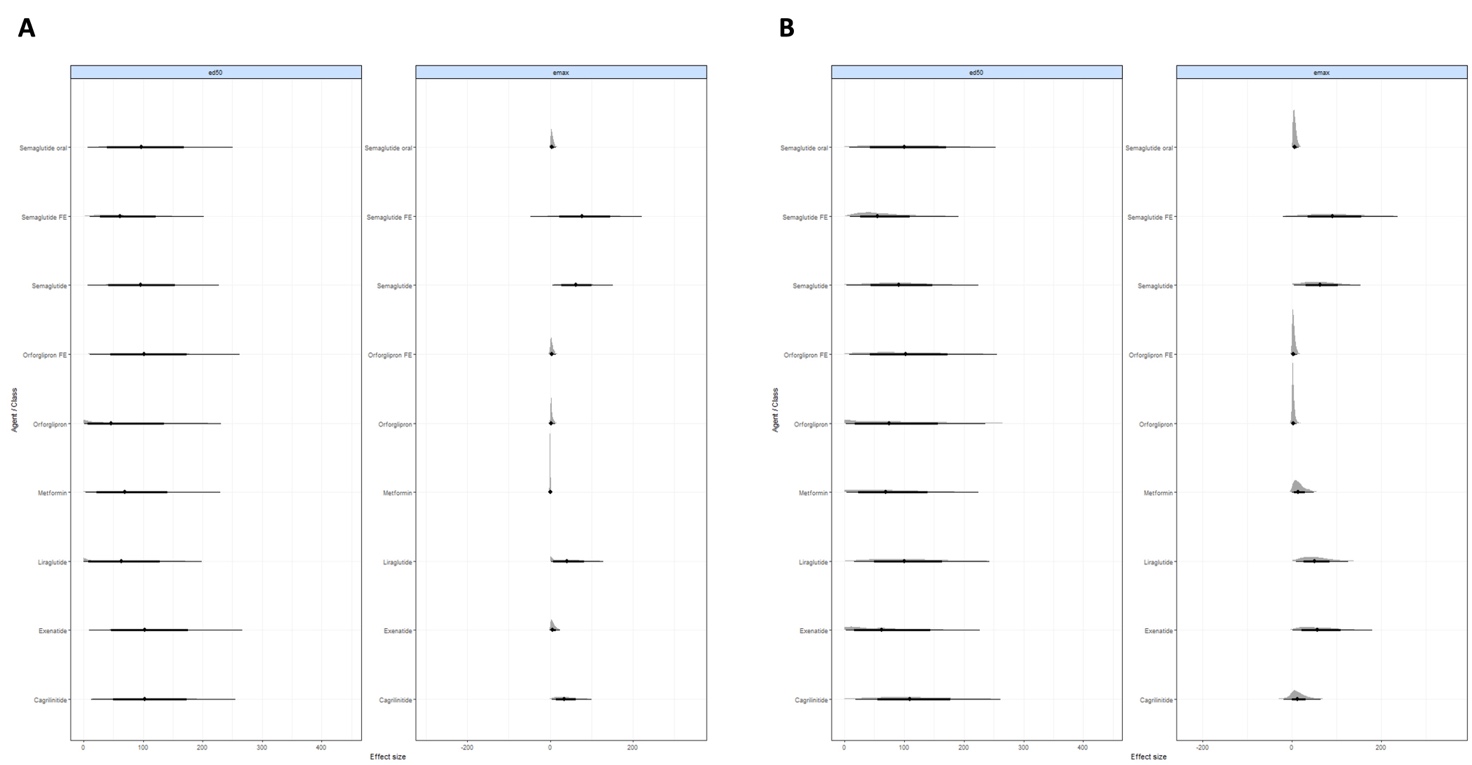
**

**
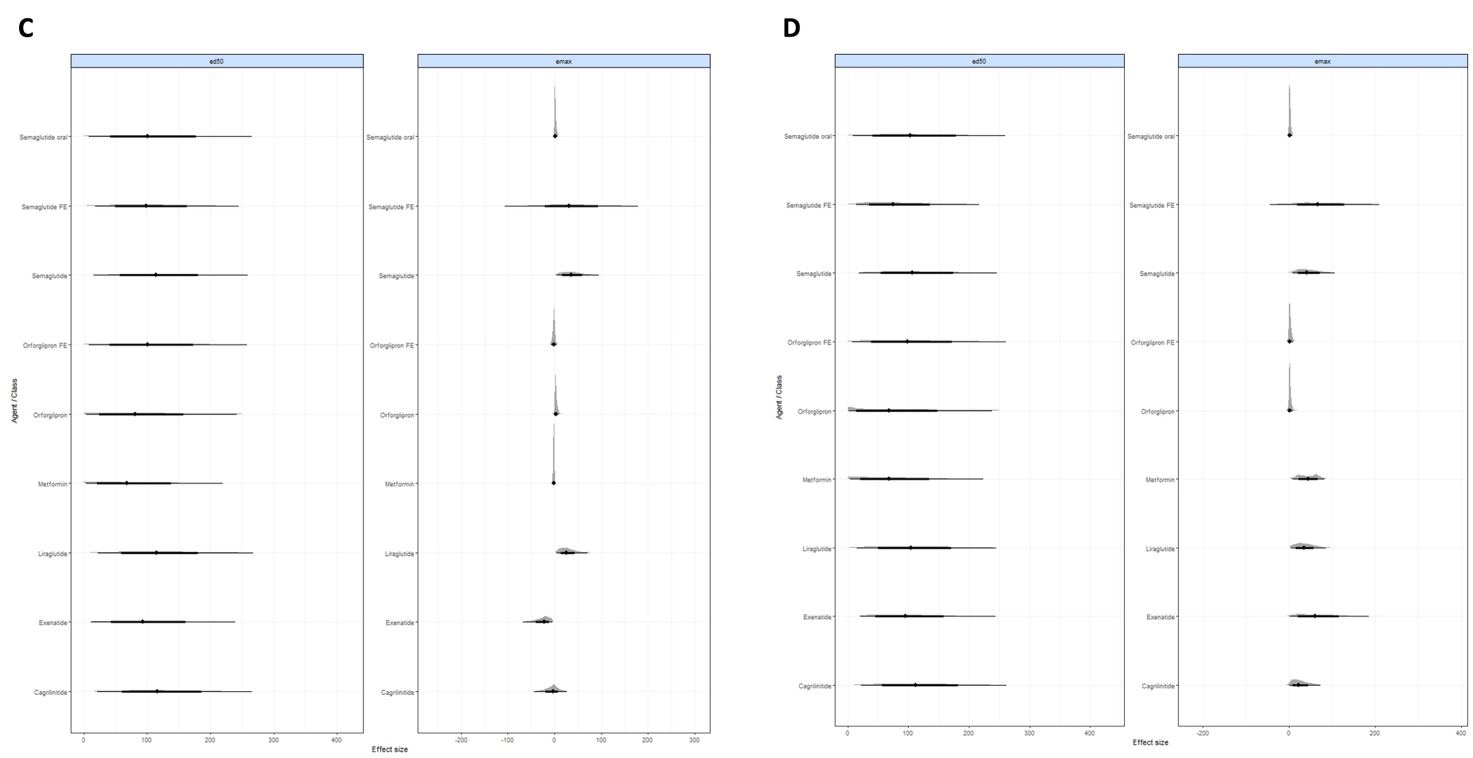
**

**
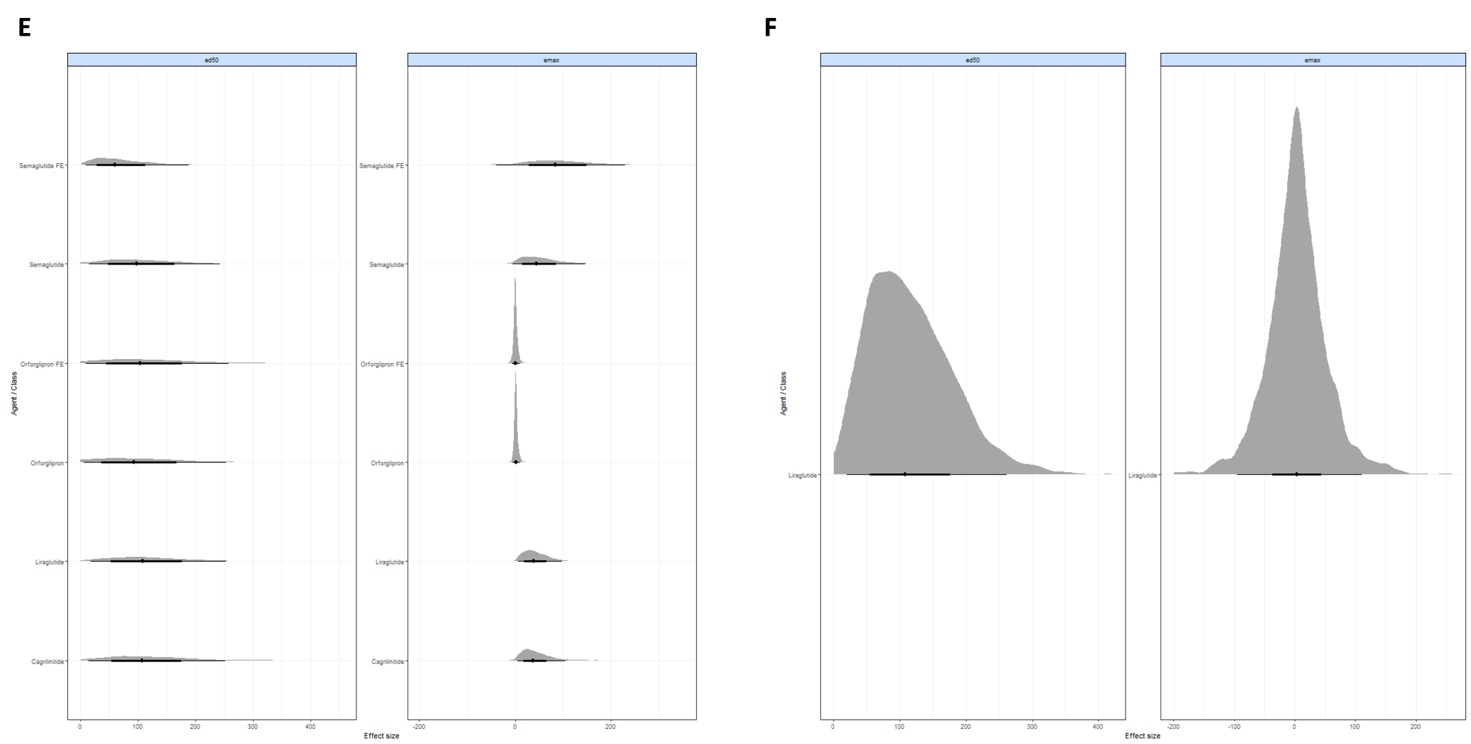
**

**Supplementary figure 4.** Network meta-analysis forest plot for (A) nausea, (B) vomiting, (C) diarrhea, (D) constipation, (E) decreased appetite, and (F) gallstone-related.

**
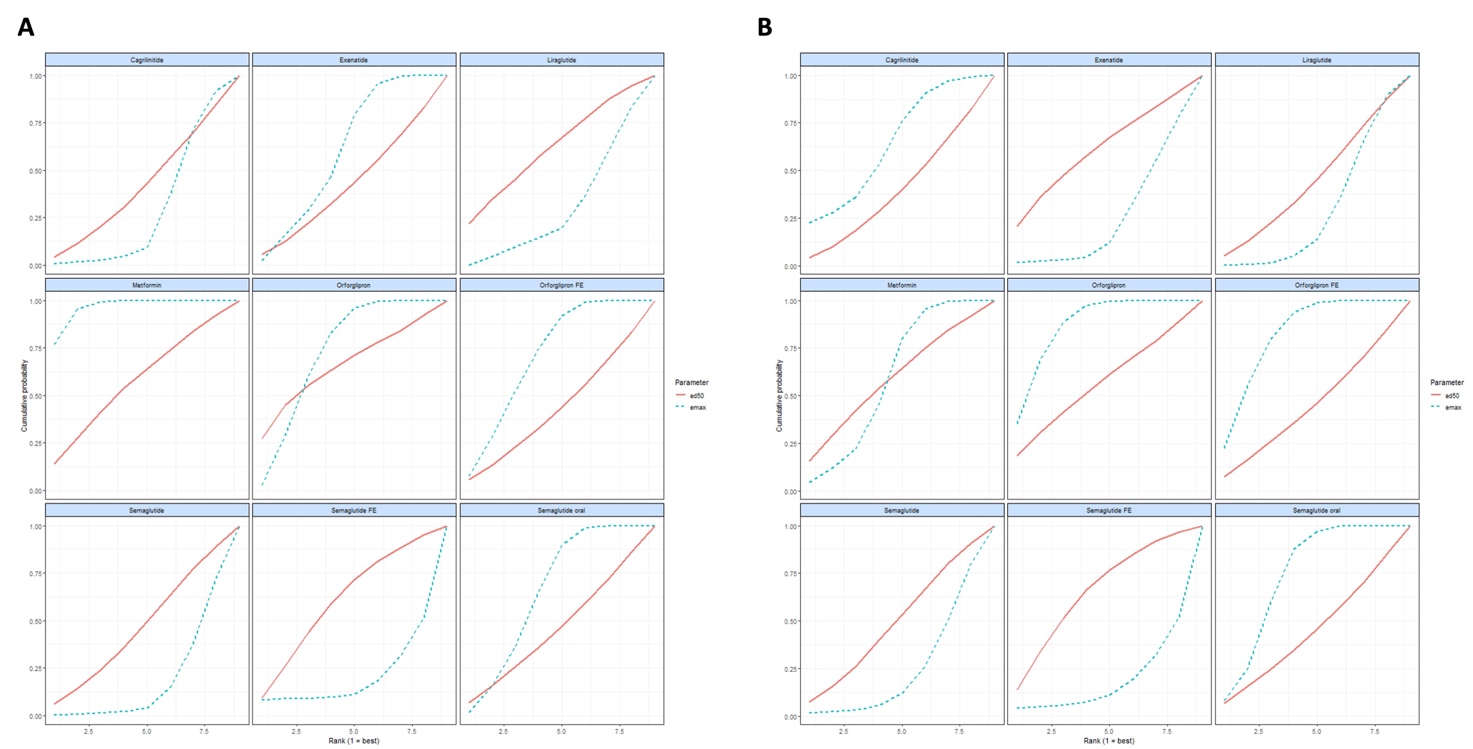
**

**
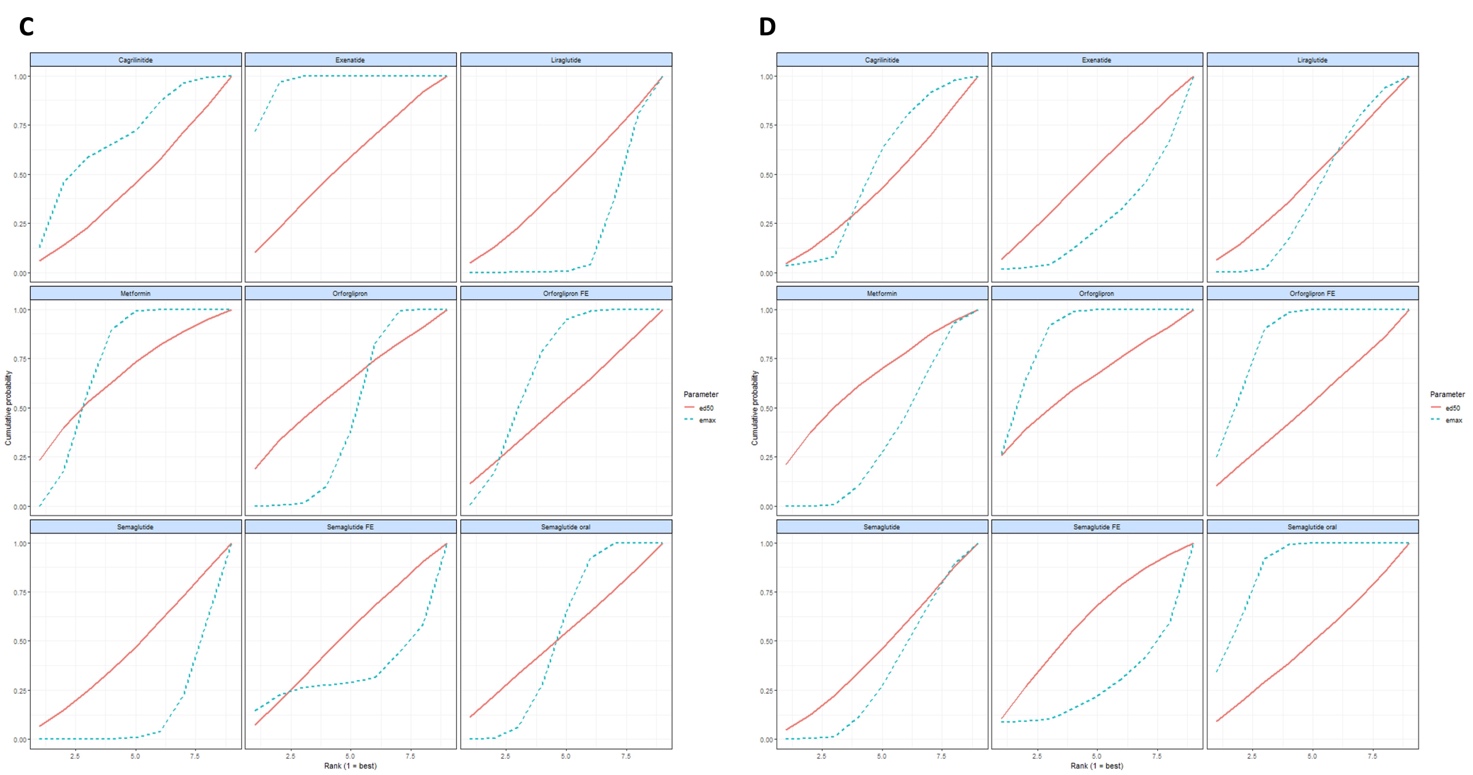
**

**
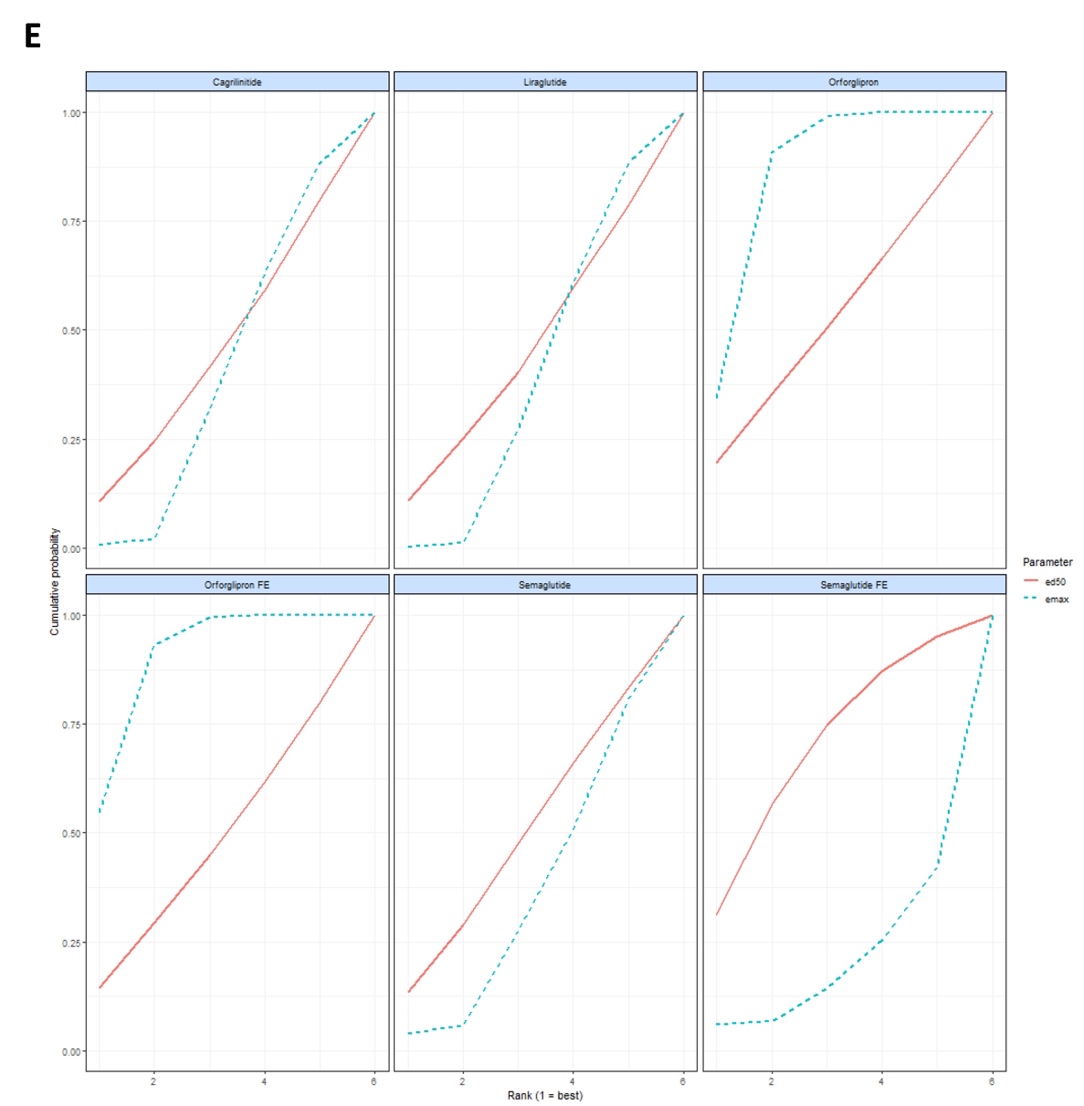
**

**Supplementary figure 5.** Network meta-analysis cumulative ranking for (A) nausea, (B) vomiting, (C) diarrhea, (D) constipation, and (E) decreased appetite.

**
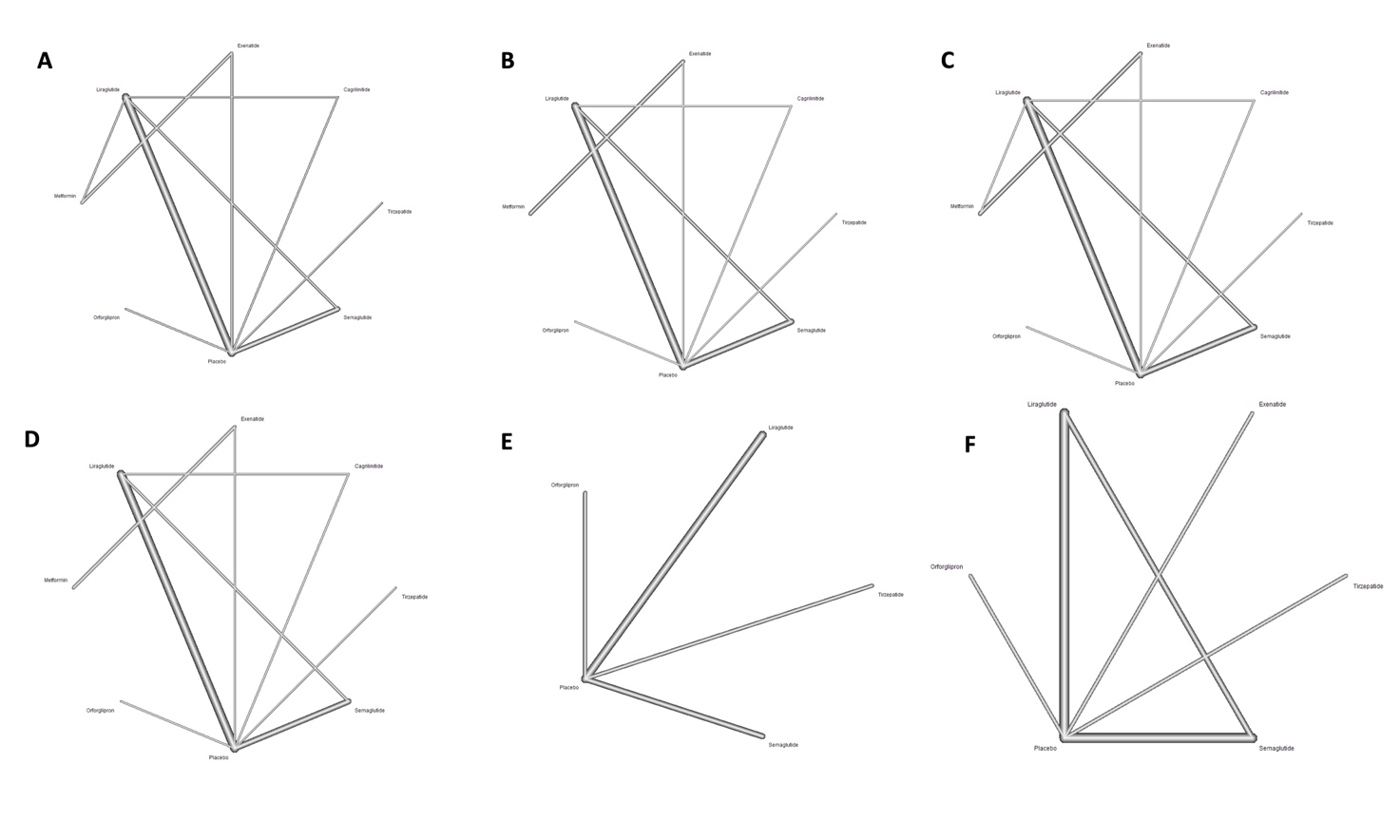
**

**Supplementary figure 6.** Network plot for (A) nausea, (B) vomiting, (C) diarrhea, (D) constipation, (E) GERD, and (F) eructation.

**
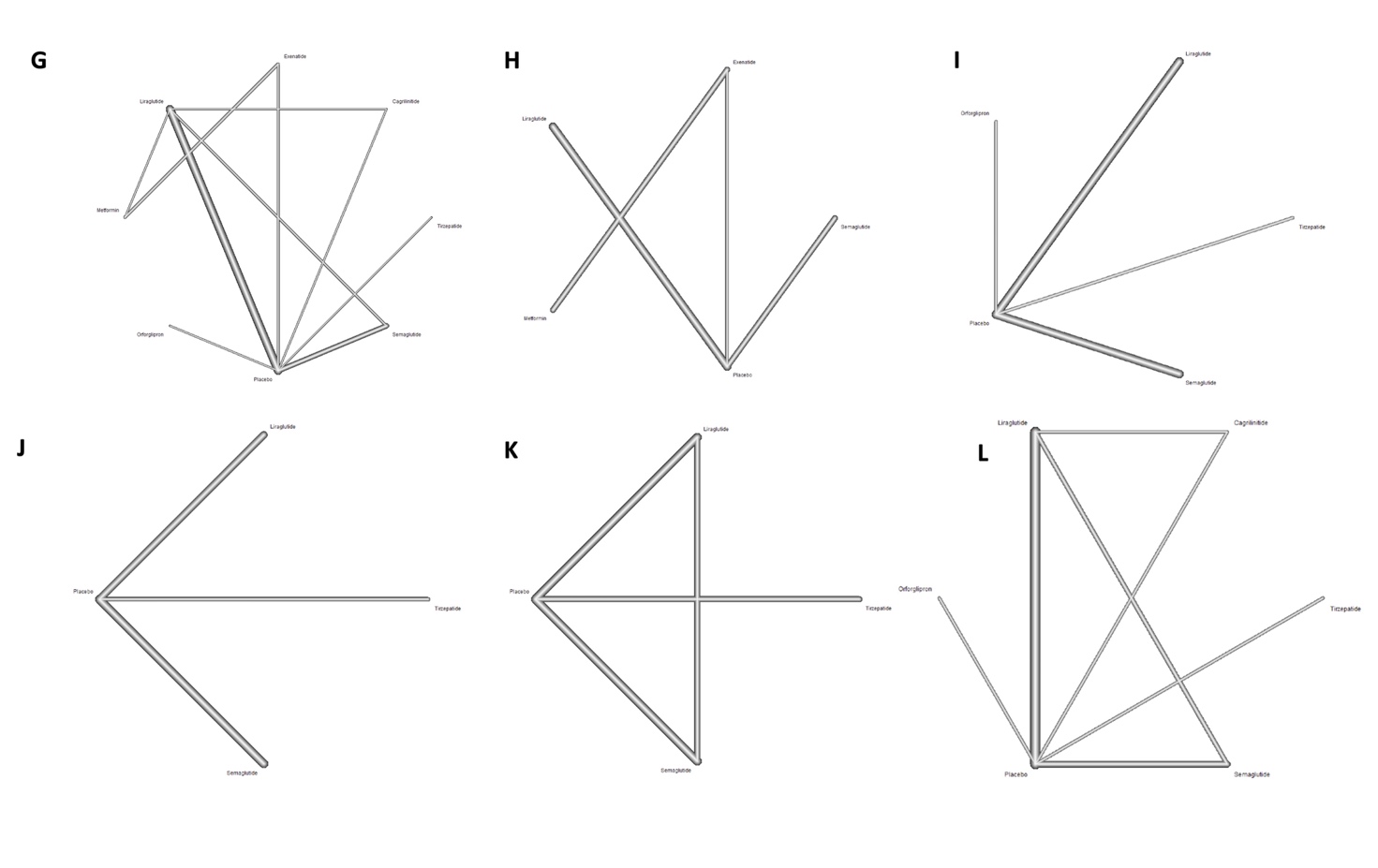
**

**Supplementary figure 6.** Network plot for (G) abdominal distention, (H) abdominal pain upper, (I) abdominal pain, (J) flatulence, (K) cholelithiasis, and (L) decreased appetite.

**
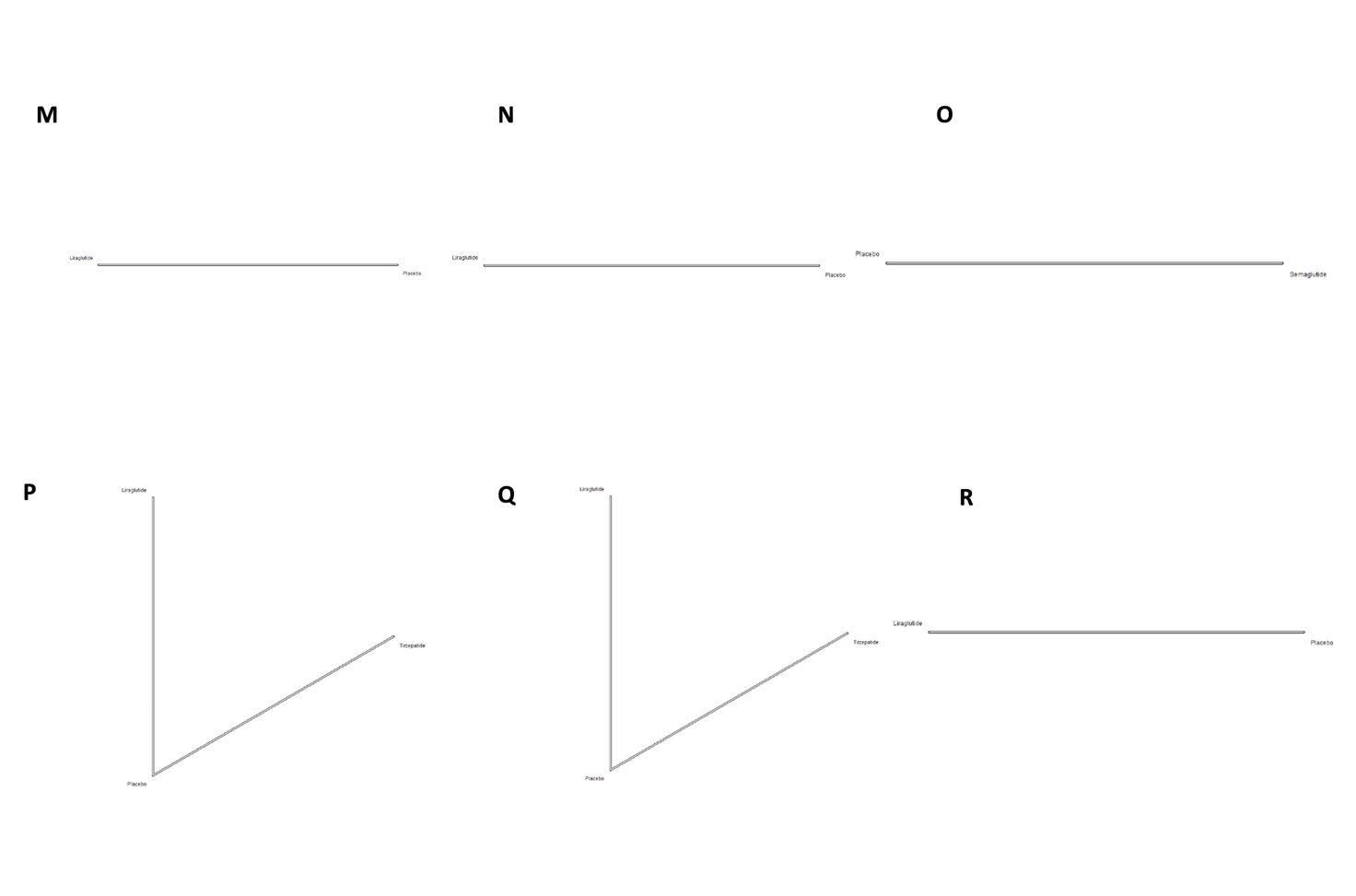
**

**Supplementary figure 7.** Network plot for (M) abdominal discomfort, (N) gallstone-related, (O) viral gastroenteritis, (P) cholecystitis, (Q) acute cholecystitis, and (R) acute pancreatitis.


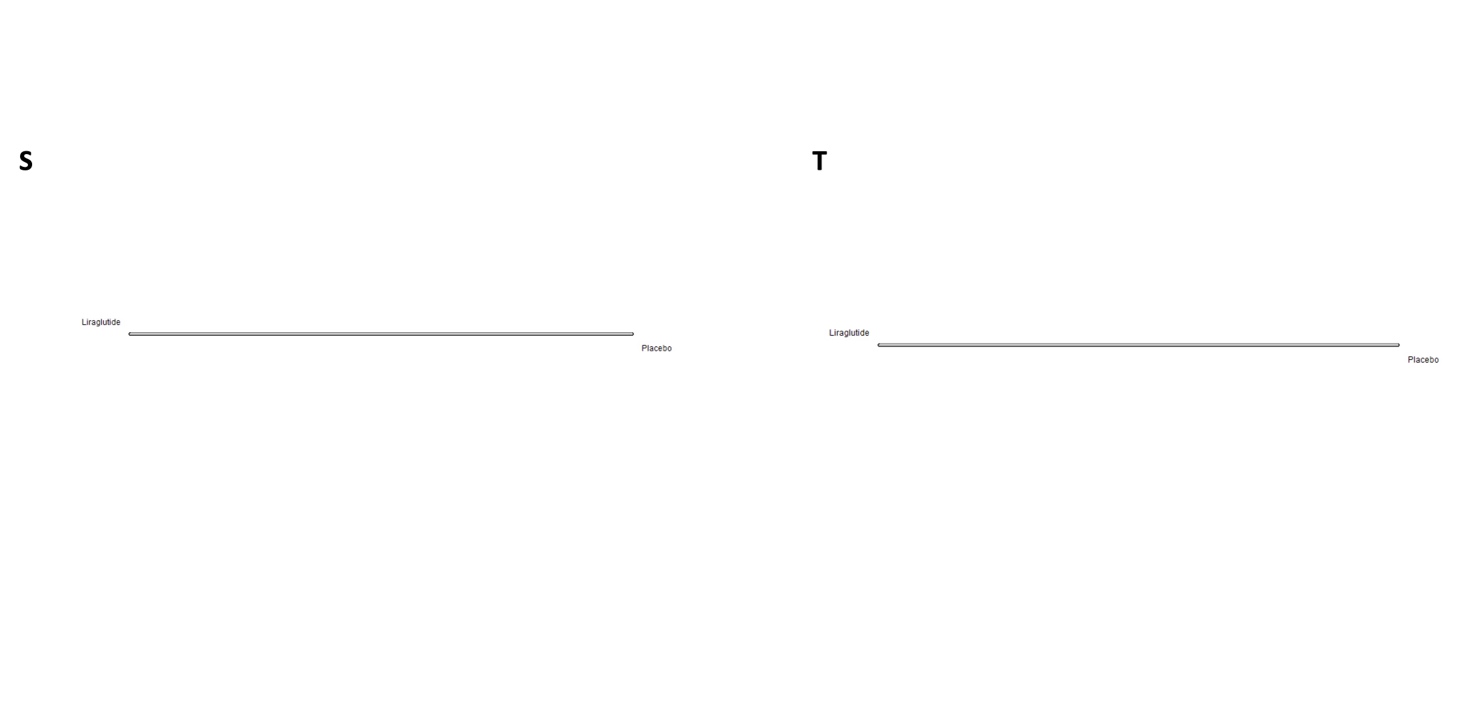


**Supplementary figure 8.** Network plot for (S) hard feces, and (T) infrequent bowel movements.


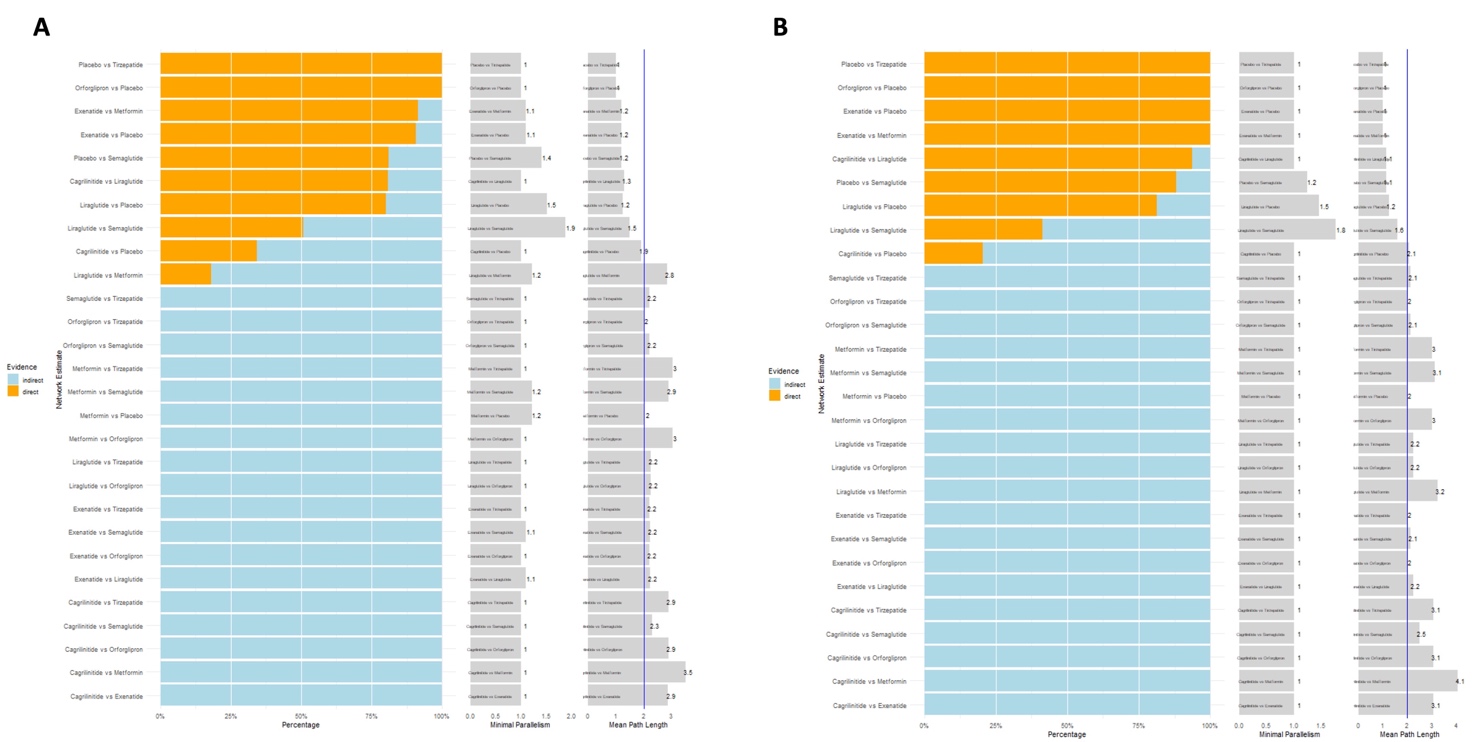


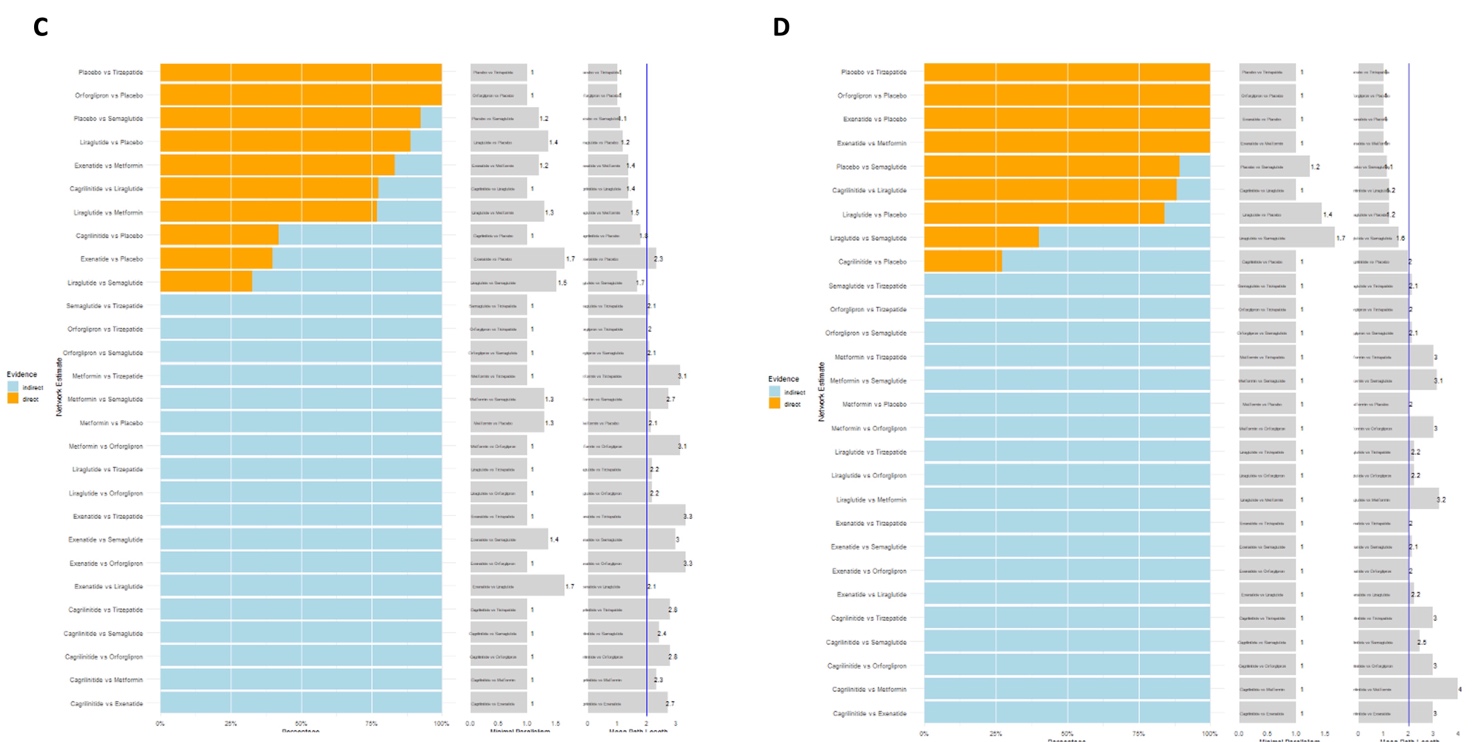


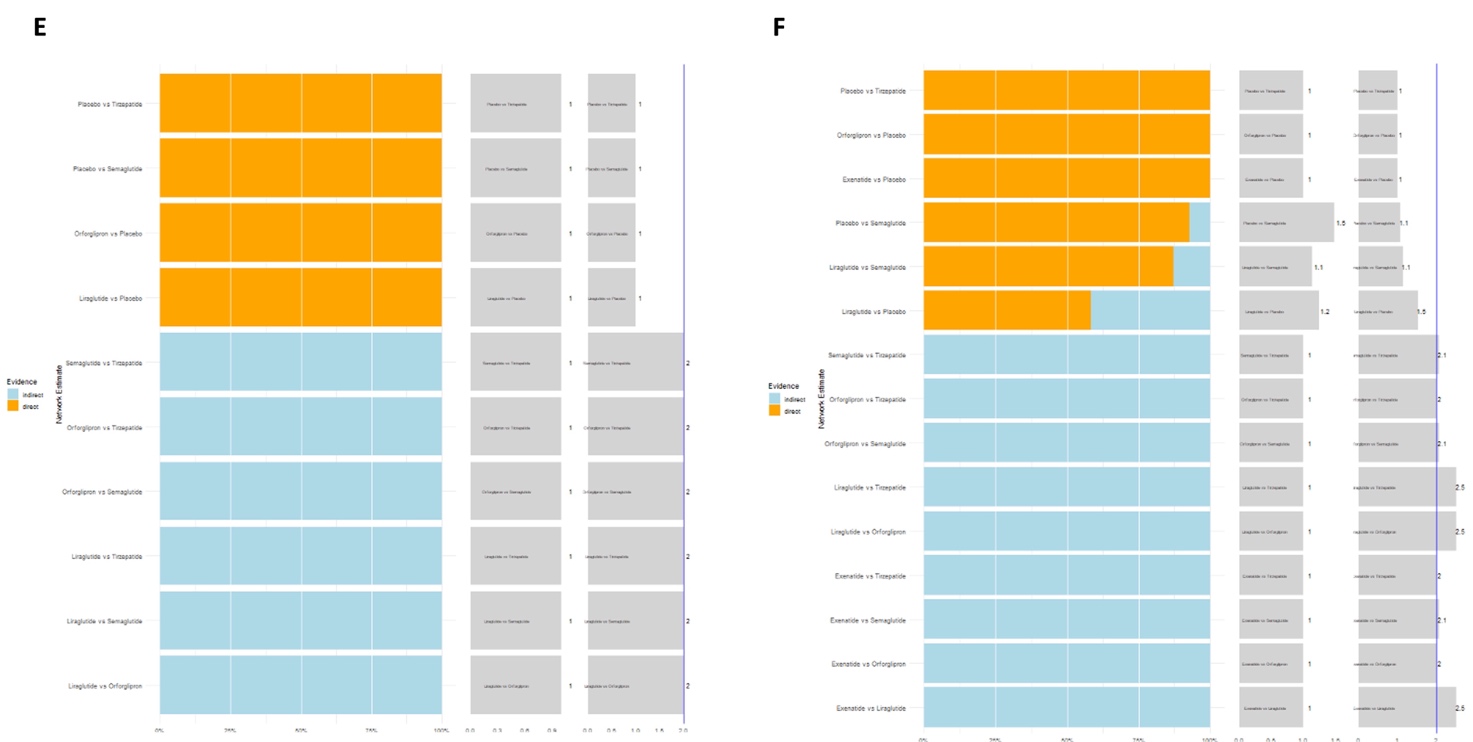


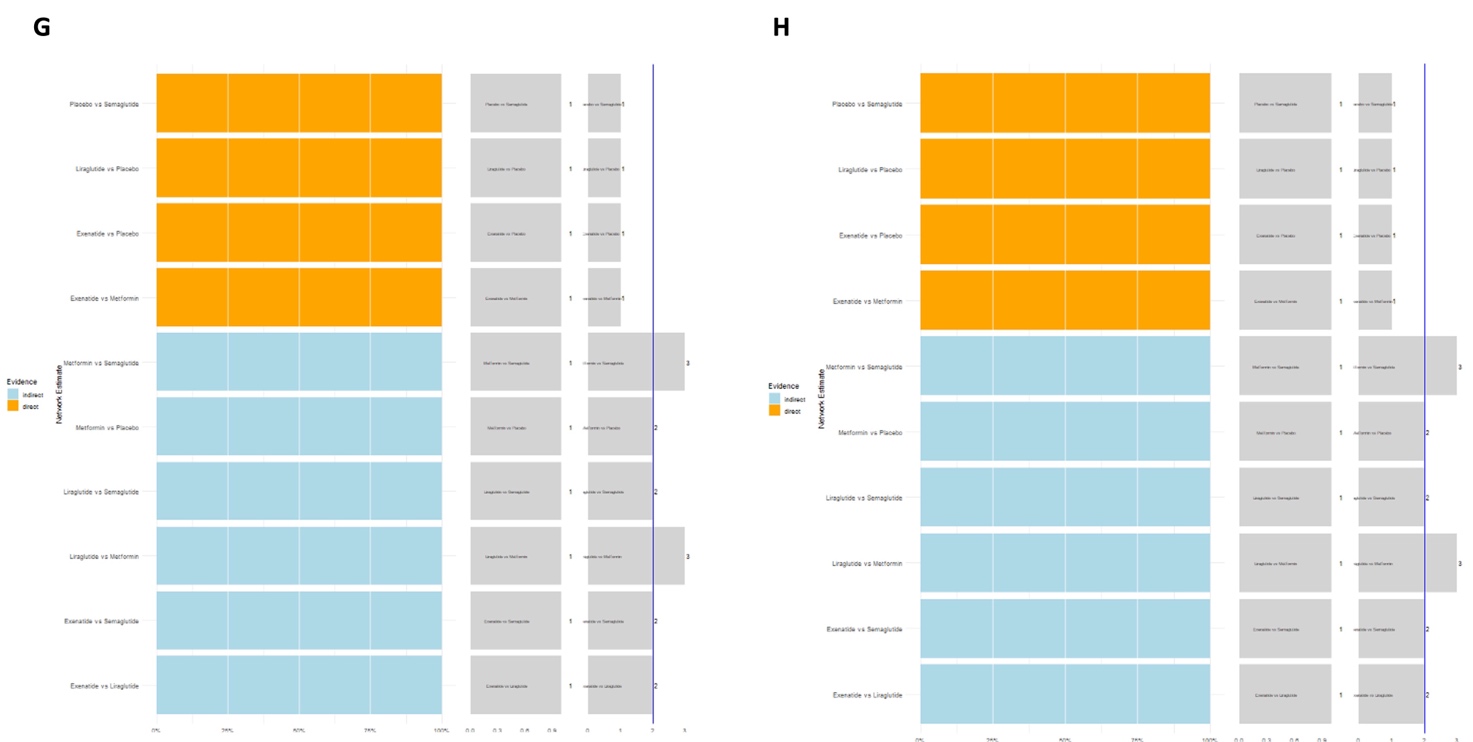


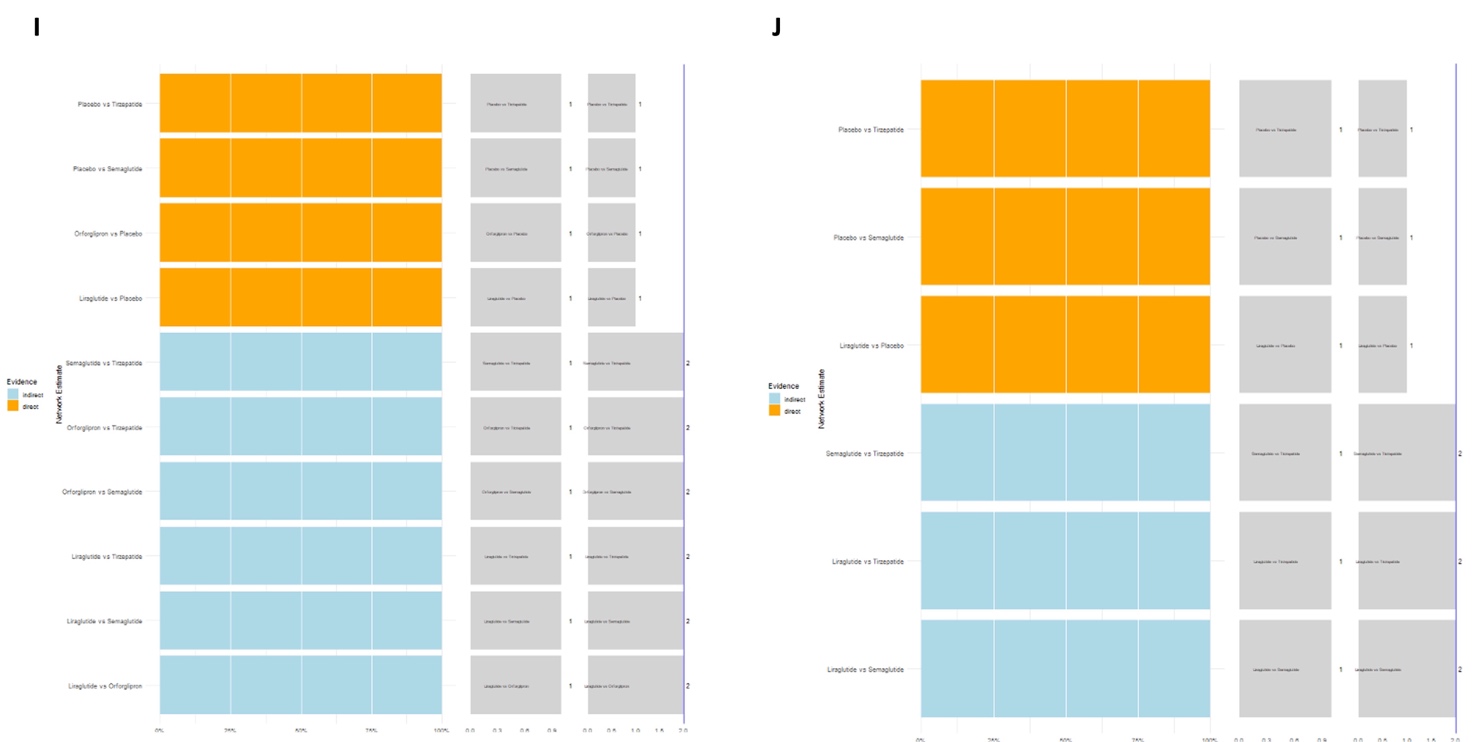


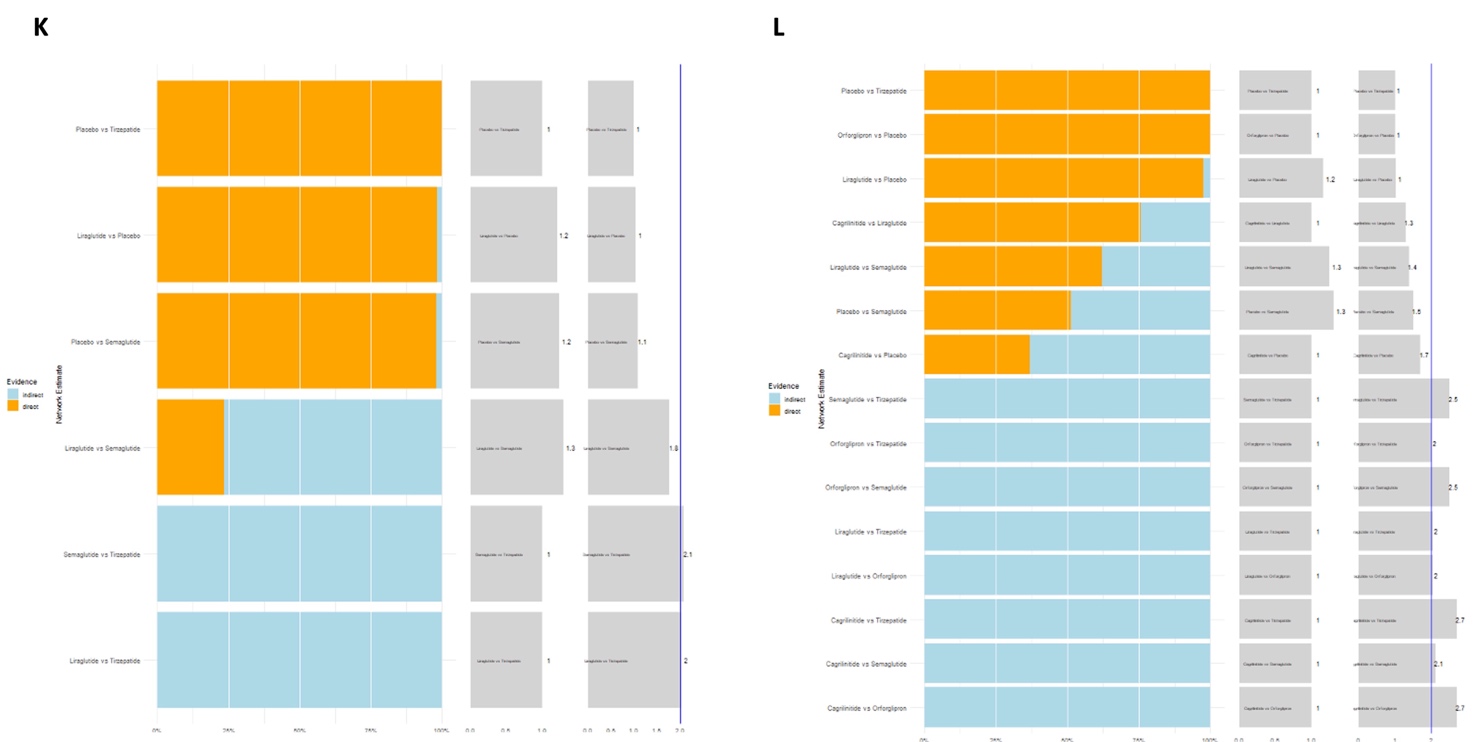


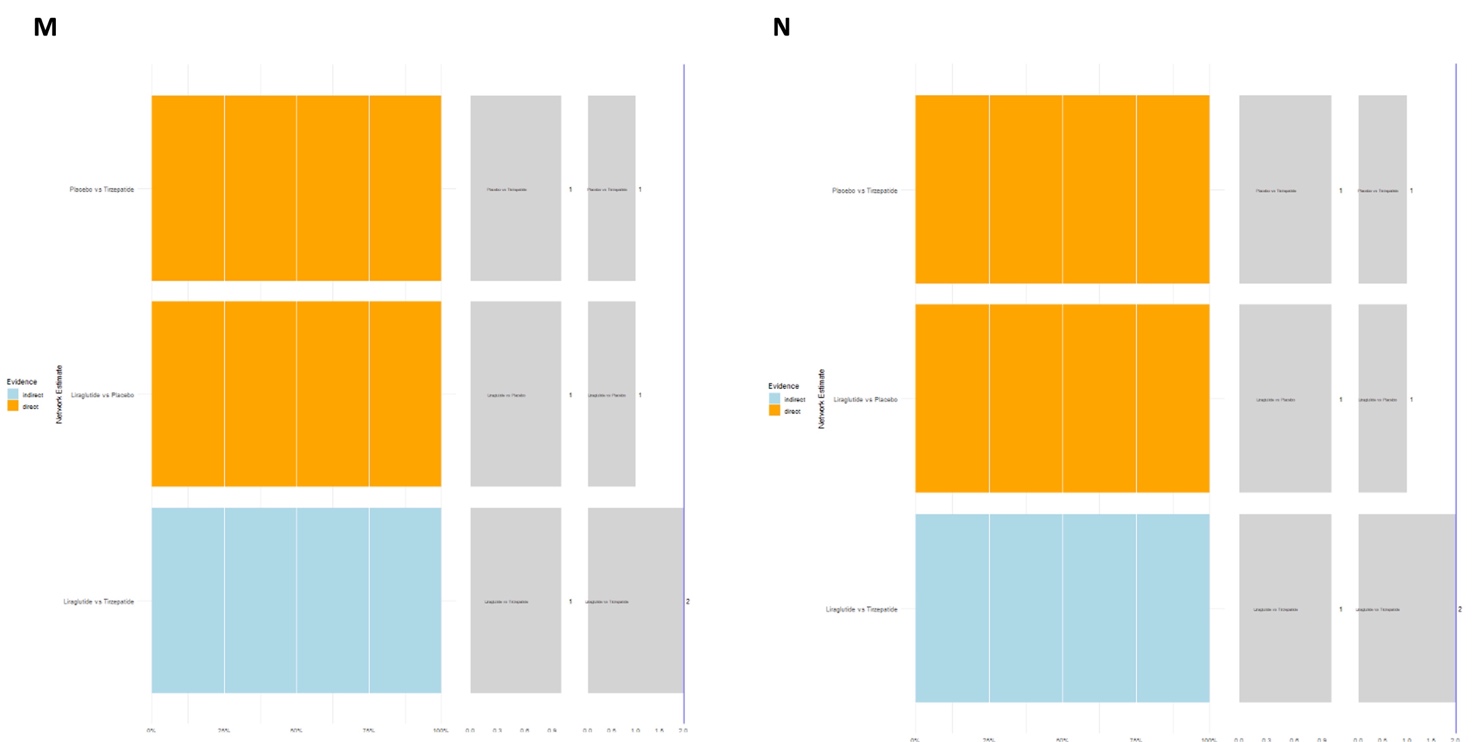


**Supplementary figure 9.** Fixed-effect model for (A) nausea, (B) vomiting, (C) diarrhea, (D) constipation, (E) GERD, (F) eructation, (G) abdominal distention, (H) abdominal pain, (I) abdominal pain, (J) flatulence, (K) cholelithiasis, (L) decreased appetite, (M) cholecystitis, and (N) acute cholecystitis.


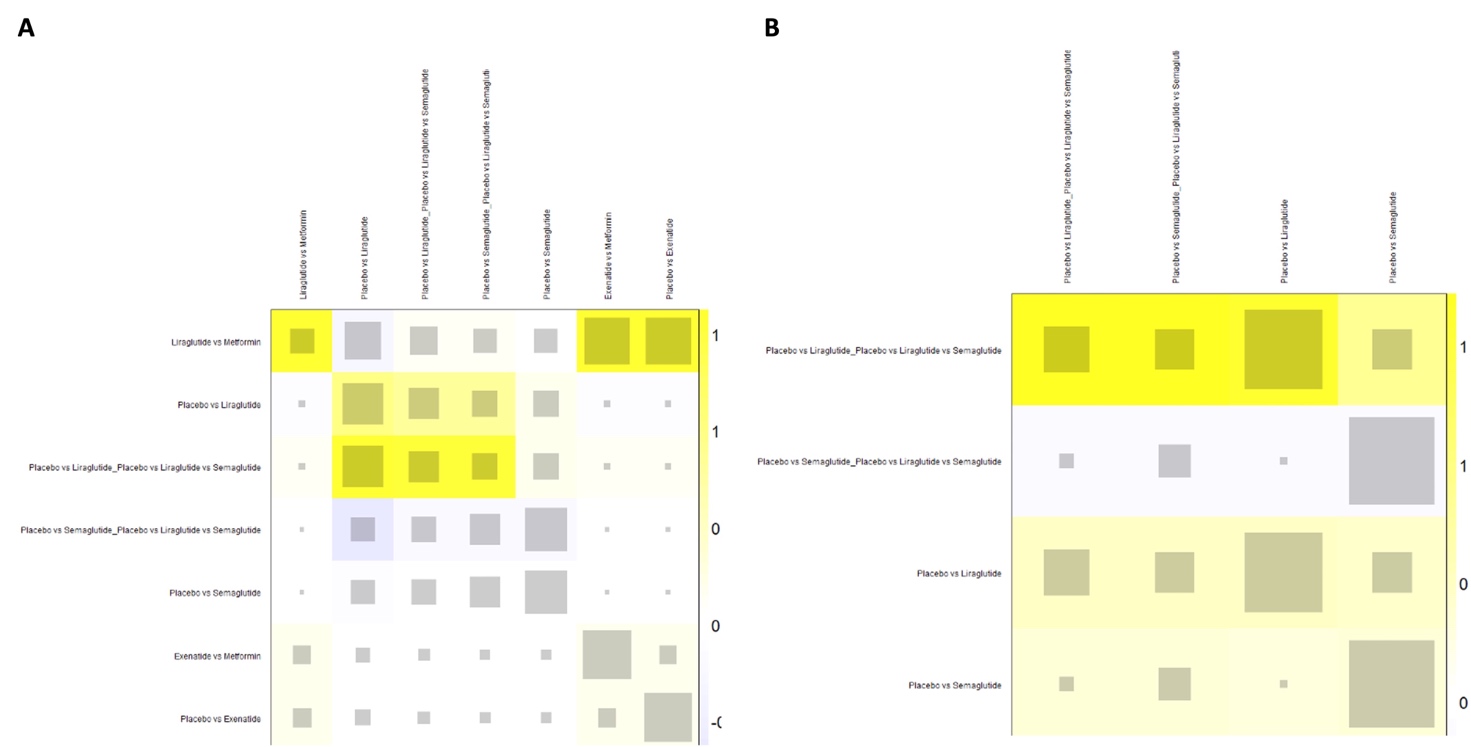


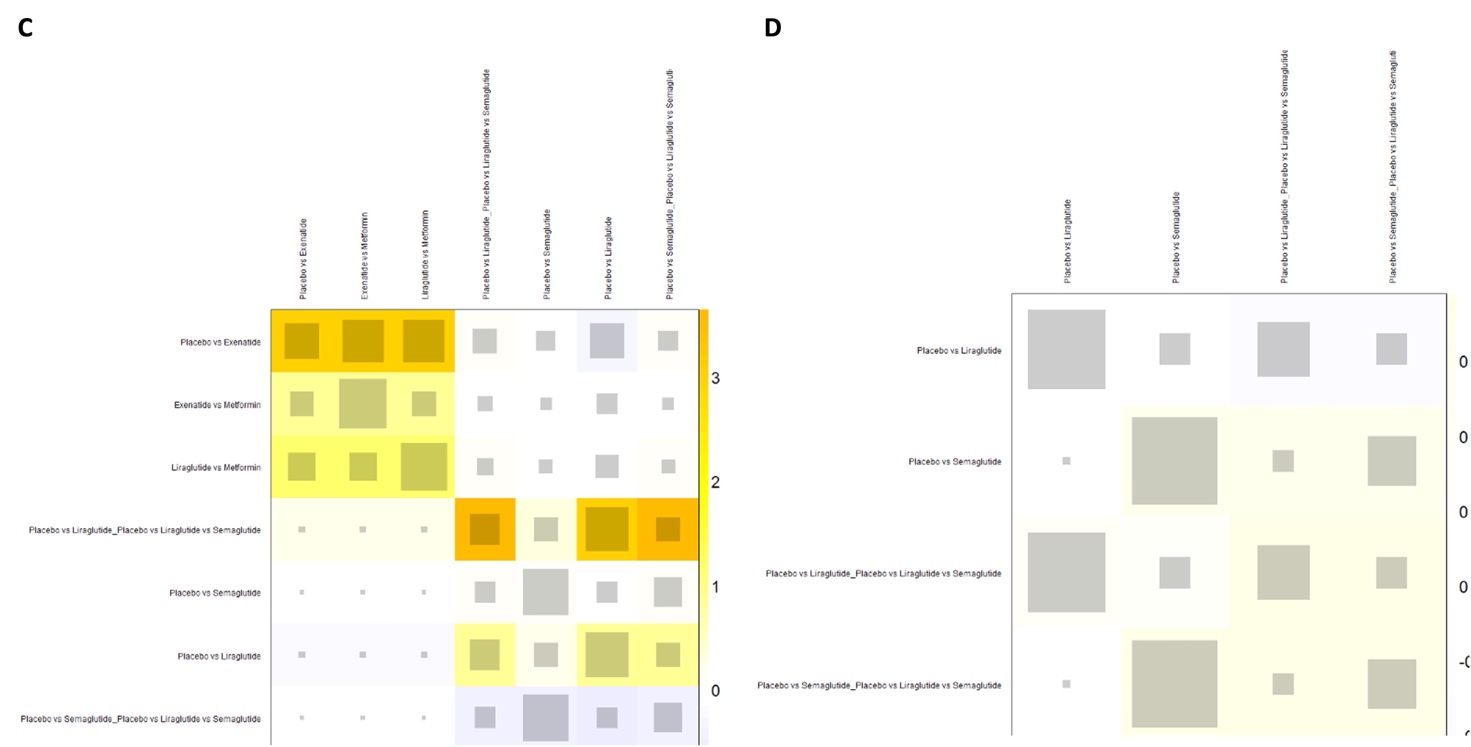


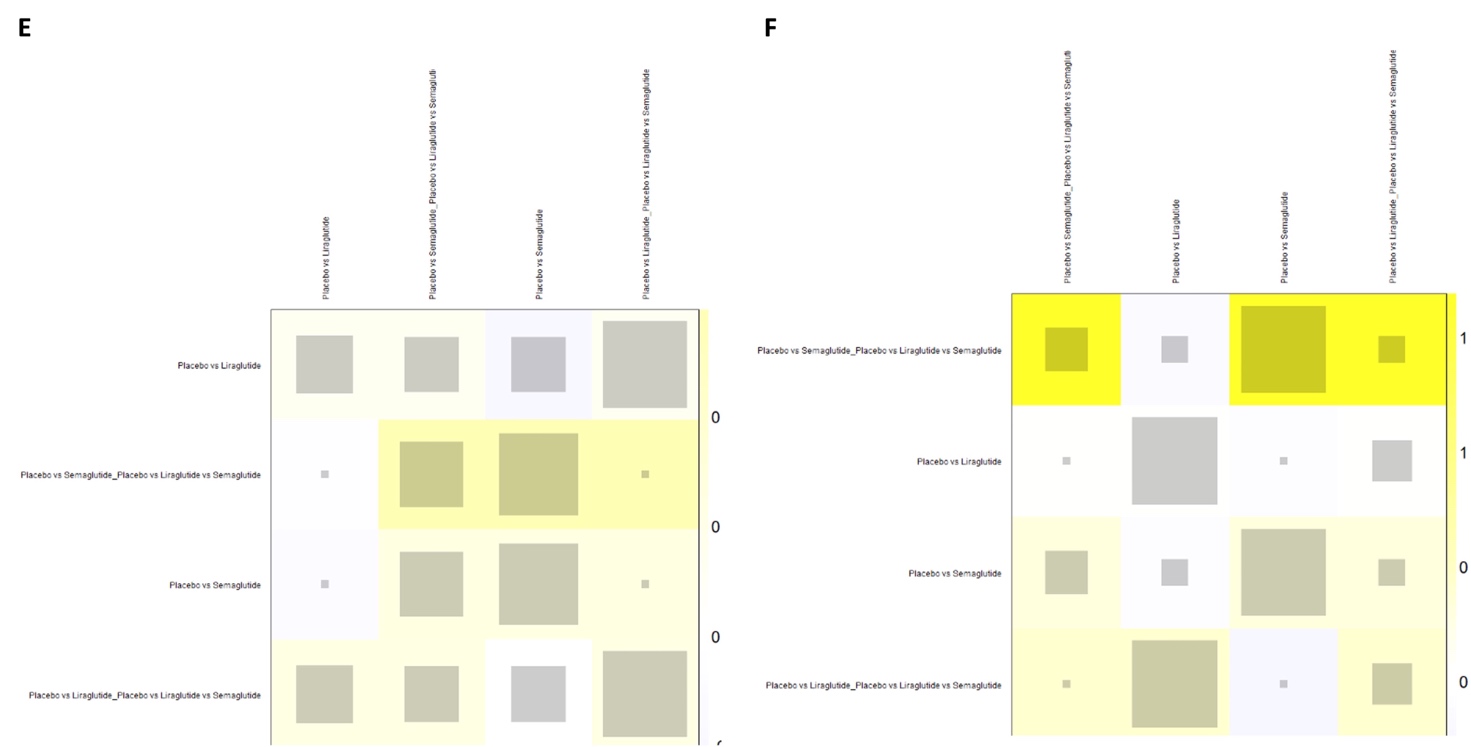


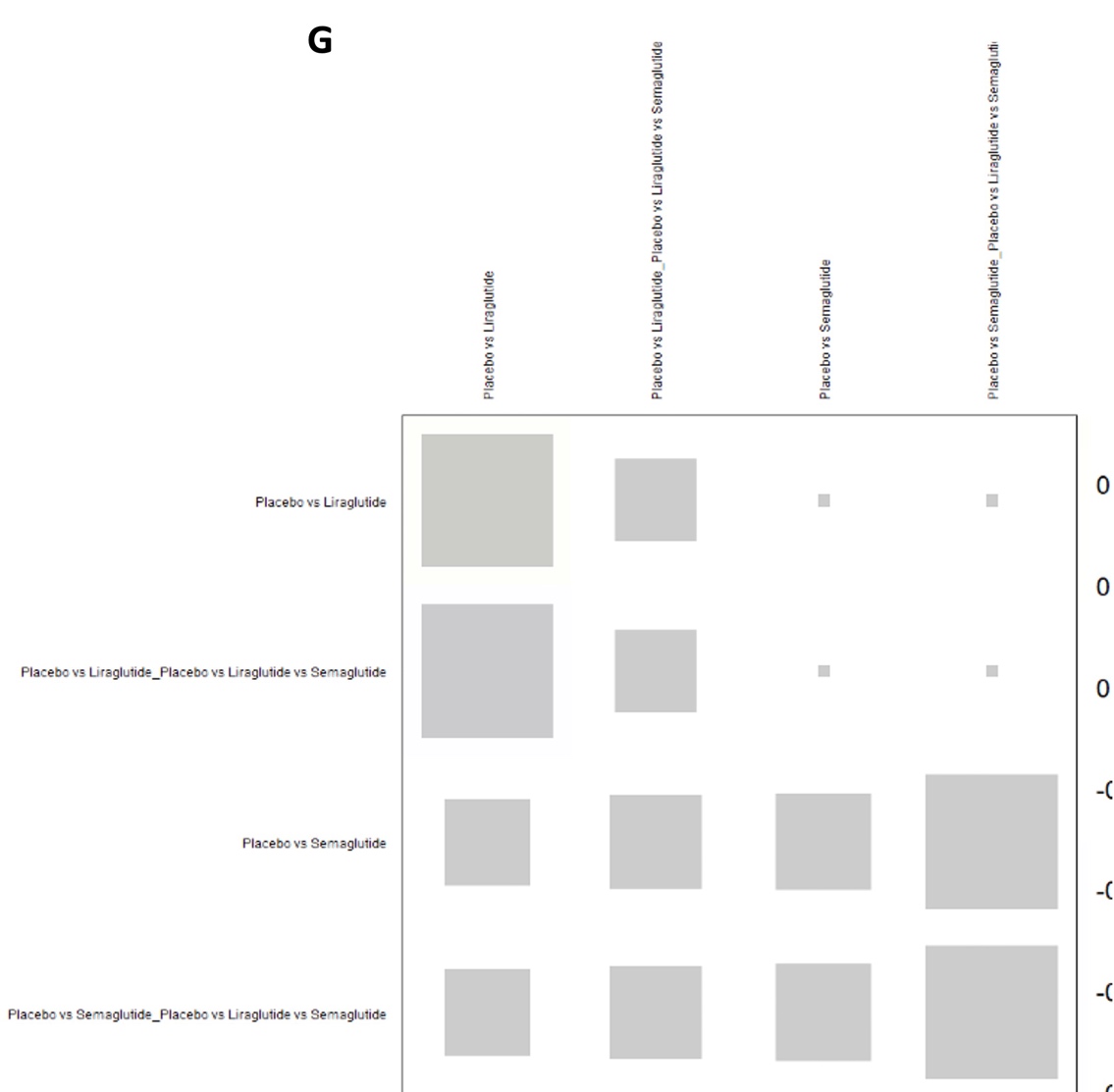


**Supplementary figure 10.** Heat plot for (A) nausea, (B) vomiting, (C) diarrhea, (D) constipation, (E) eructation, (F) cholelithiasis, and (G) decreased appetite.
